# A Waterborne Epoxy Vitrimer: Enabling Moisture‐Driven Actuation, Continuous Moist‐Electric Generation, and Water‐Assisted Degradation

**DOI:** 10.1002/advs.202513579

**Published:** 2025-10-08

**Authors:** Jianqiao Wu, Yan Zhang, Dongxu Pei, Yaning Ma, Zixuan Wang, Qiuyang Ma, Jianhua Tang, Ousheng Zhang, Jun Hu

**Affiliations:** ^1^ College of Materials and Chemical Engineering Chuzhou University Chuzhou Anhui Province 239000 China; ^2^ Beijing Advanced Innovation Center for Soft Matter Science and Engineering Beijing University of Chemical Technology Beijing 100029 China; ^3^ Sinopec (Shanghai) Petrochemical Research Institute Co., Ltd Shanghai 201208 China

**Keywords:** epoxy, moist‐electric generator, moisture‐driven actuation, waterborne vitrimer

## Abstract

The integration of multiple hydroxyl structures presents a robust strategy for designing epoxy vitrimers based on transesterification reactions (TERs), enabling the realization of optimal dynamic properties. Nevertheless, significant challenges persist in achieving eco‐friendly synthesis, hydrophilic‐related functionalities, and sustainable degradation. Here, a catalyst‐free waterborne epoxy vitrimer is developed by curing 1,4‐butanediol diglycidyl ether with natural *L*‐tartaric acid (*L*‐TA), effectively addressing these challenges simultaneously. Harnessing the synergistic effects of multiple hydroxyl groups and the neighboring group participation effect imparted by *L*‐TA, the resulting vitrimer exhibits rapid stress relaxation (*τ* ^*^ = 270 s at 190 °C) and undergoes complete degradation in 95 °C pure water within 6 h. Furthermore, the abundant hydroxyl groups on polymer chains facilitate its function as a moisture‐responsive actuator, achieving a crimping speed of 0.32 mm s^−1^. Building on this, a moist‐electric generator is engineered by utilizing the vitrimer as a matrix for ambient vapor collection and subsequent conversion into electrical energy. This device sustains a stable voltage output (0.42–0.50 V) under ambient conditions for 60 h, with capabilities for series amplification and capacitor charging. This study maximizes the potential of hydroxyl‐rich TERs‐based epoxy vitrimers, paving the way for the advancement and practical implementation of sustainable polymers.

## Graphical abstract

A catalyst‐free waterborne epoxy vitrimer has been developed through the curing of 1,4‐butanediol diglycidyl ether with L‐tartaric acid. The incorporation of abundant hydroxyl groups and the neighboring group participation effect enable this vitrimer to exhibit rapid stress relaxation and achieve complete degradation in hot water. Additionally, it demonstrates versatile functionality, serving as both a moisture‐driven actuator and a moist‐electric generator.

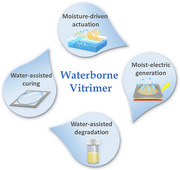

## 1 Introduction

Vitrimer, a typical dynamic cross‐linked polymer,^[^ ^1^ ^]^ behaves like thermosets at relatively low temperature, while maintaining thermoplasticity at elevated temperature due to the bond exchange reaction‐promoted topological network rearrangements. Among all reported vitrimers, epoxy vitrimers utilizing transesterification reactions (TERs) represent not only the earliest discovered class but also the most extensively studied system.^[^ ^1^, ^2^, ^3^, ^4^, ^5^ ^]^ Their widespread adoption stems from their compatibility with conventional epoxy curing processes, making them particularly suitable for industrial‐scale production. Meanwhile, the ester bonds in epoxy vitrimers provide a robust foundation for achieving enhanced mechanical and thermal properties. This unique characteristic has driven extensive research of TERs‐based epoxy vitrimers, leading to their successful implementation in diverse applications such as advanced adhesives,^[^ ^6^, ^7^ ^]^ liquid crystal elastomers,^[^ ^8^, ^9^ ^]^ shape memory materials,^[^ ^10^, ^11^ ^]^ and carbon fiber reinforced composites.^[^ ^12^ ^,^ ^13^ ^]^

Since the discovery of epoxy vitrimers, one of the biggest concerns has been the precise regulation of network rearrangement to optimize their dynamic properties. In their pioneering study in 2012, Leibler and colleagues^[^ ^14^ ^]^ demonstrated that external catalysts could effectively modulate the network rearrangement rate, revealing a positive correlation between catalyst concentration and TERs kinetics. However, this extrinsic regulation approach presents inherent limitations, including a restricted processing window and compromised thermal stability, which significantly constrain practical applications. Consequently, a series of intrinsic strategies emerge endlessly, such as enhancing the flexibility of polymer chains,^[^ ^15^ ^]^ optimizing cross‐linking density,^[^ ^16^ ^]^ designing dual‐dynamic covalent bond systems,^[^ ^17^ ^]^ incorporating neighboring group participation effects,^[^ ^18^, ^19^ ^]^ and modulating hydroxyl group concentration.^[^ ^20^, ^21^, ^22^, ^23^, ^24^ ^]^ Among these strategies, the incorporation of multiple hydroxyl structures has become a hot yet effective way. It avoids the safety concerns associated with an external catalyst, provides abundant reactive sites to ensure optimal cross‐linking density, and improves TERs rate without deteriorating the mechanical and thermal properties. These benefits have led to the development of numerous high‐performance epoxy vitrimers with accelerated TERs rates, such as ferulic acid‐based hyperbranched epoxy resin,^[^ ^22^ ^]^ lignin‐derived repairable coating,^[^ ^23^ ^]^ and fully biobased vitrimer engineered with tannic acid tailored.^[^ ^24^ ^]^ Despite these advancements in constructing hydroxyl structure‐rich epoxy vitrimers, three critical challenges persist. First, the hydrophobicity of these materials severely limits their applicability in hydrophilic environments, such as oil/water separation membranes, solar evaporators, and moisture‐driven actuators. Second, achieving a sustainable degradation remains elusive, as current methods often involve high temperatures, toxic solvents, and low efficiency. Third, the curing process is not environmentally friendly, typically requiring high temperatures and organic solvents, particularly when dissolving multi‐hydroxyl monomers in hydrophobic epoxy resins. Therefore, the creation of hydrophilic and sustainably degradable epoxy vitrimers continues to pose a big challenge.

**Figure 1:**
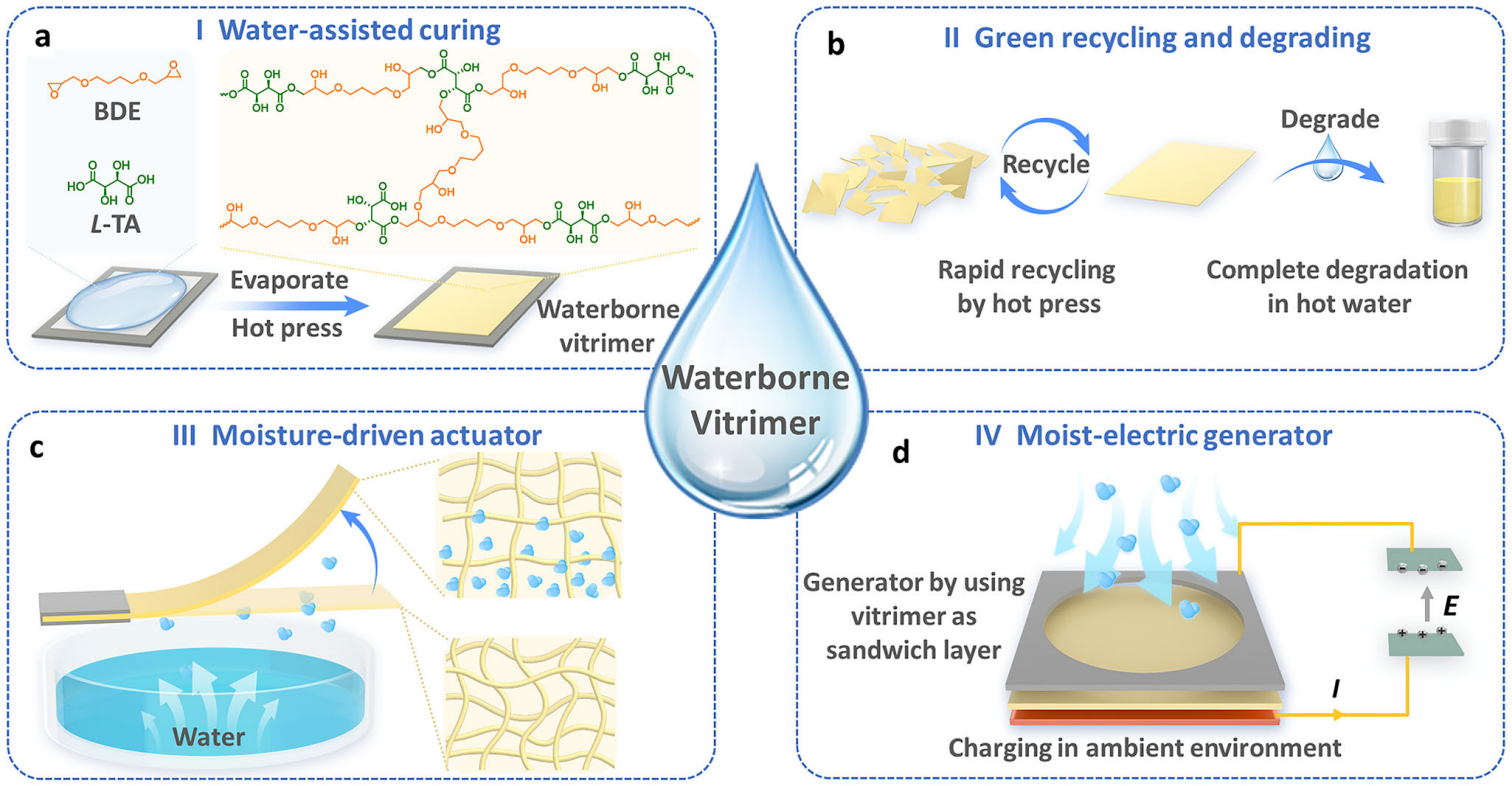
Schematic illustration of the preparation and properties of waterborne epoxy vitrimer. a) Preparation of waterborne vitrimer through the evaporation of BDE and *L*‐TA in pure water, followed by a hot‐pressing procedure. b) Demonstration of green recycling and degradation in hot water. c) Moisture‐responsive bending actuation under asymmetric moisture conditions. d) Moist‐electric generator employing the waterborne vitrimer as a sandwich layer for moisture absorption. The device is capable of harnessing water vapor under ambient conditions to charge a capacitor.

In this work, we have devised this kind of waterborne epoxy vitrimers through a green, effective strategy (**Figure** **1**, section I), where natural *L*‐tartaric acid (*L*‐TA) as curing agent was mixed with 1,4‐butanediol diglycidyl ether (BDE) in pure water, followed by water evaporation and hot curing. This approach effectively mitigated compatibility issues between different monomers, where BDE could be cured with *L*‐TA to result in a homogenous solution. Meanwhile, the flexibility of BDE ensured material for actuation. *L*‐TA, a biomass derivative that can be extracted from coarse tartar,^[^ ^25^ ^]^ features two carboxyl groups and two hydroxyl groups. These functional groups not only enriched the epoxy networks with abundant hydroxyl groups but also introduced the neighboring group participation (NGP) effect, thereby facilitating the TERs‐induced topological rearrangements. The resulting waterborne vitrimers demonstrated rapid stress relaxation at temperatures of 150–190 °C, and were capable of being reprocessed, recycled, and completely degraded in hot water (Figure 1, section II). Notably, the embedded hydroxyl groups enabled the waterborne vitrimers to function as a moisture‐driven actuator (Figure 1, section III) and a moist‐electric generator (Figure 1, section IV), capable of sustaining a voltage output of 0.42–0.50 V for 60 h and charging capacitors in ambient conditions. This work fully leverages the potential of multiple hydroxyl structures in TERs‐based vitrimers, offering a novel and streamlined strategy for the design and application of waterborne vitrimers.

## 2 Results and Discussion

### 2.1 Preparation, Thermal, and Mechanical Properties of Waterborne Vitrimers

**Figure 2:**
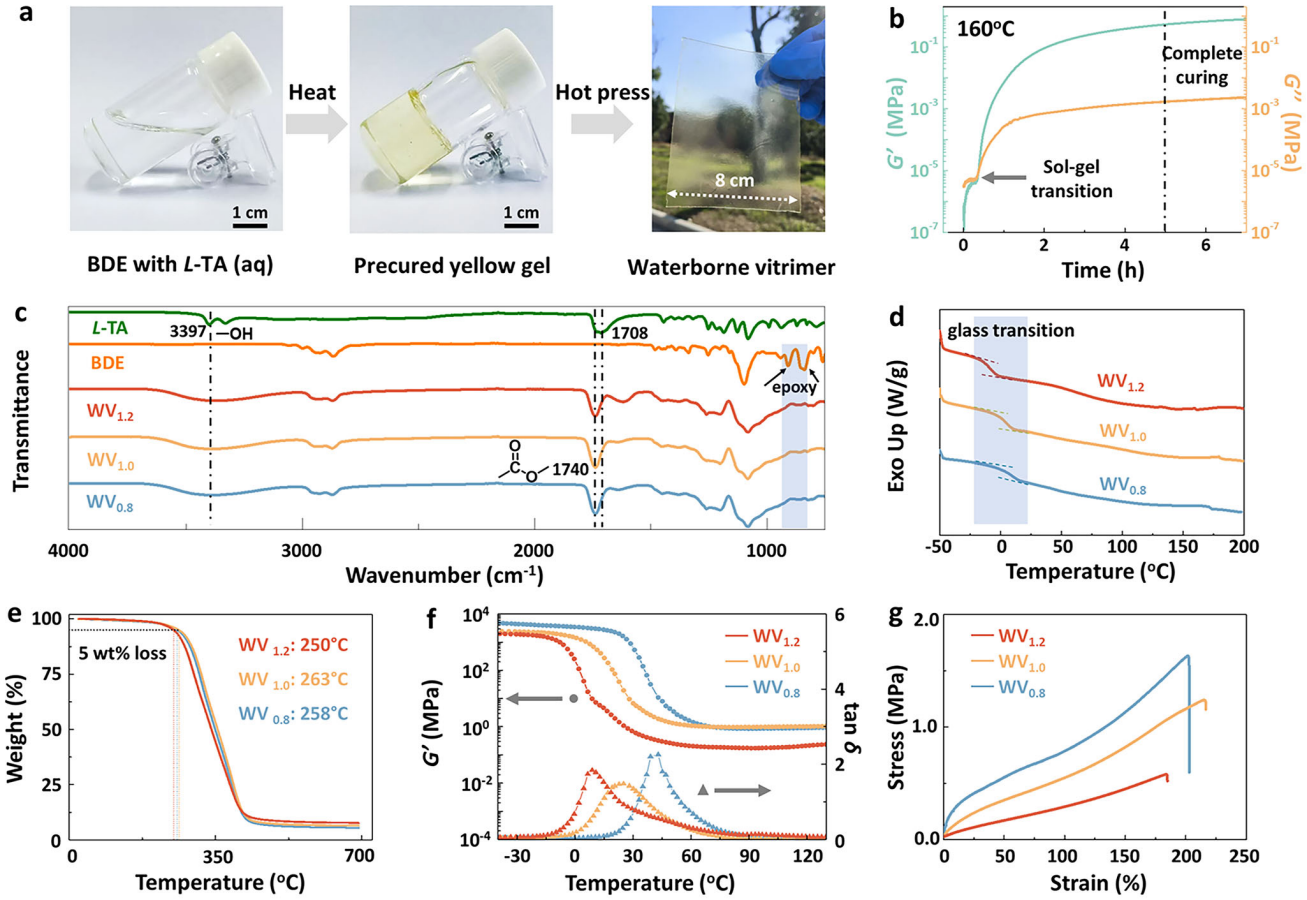
Preparation, thermal, and mechanical properties of waterborne vitrimers. a) Photographs illustrating the preparation process of waterborne vitrimers. b) Storage modulus (*G*′, green) and loss modulus (*G*′′, orange) of precured WV_1.0_ gel at 160 °C by using a rheometer. c) FTIR spectra of *L*‐TA, BDE, WV_1.2_, WV_1.0_, and WV_0.8_ from 4000 to 500 cm^−1^. d) DSC curves at a heating speed of 10 °C min^−1^. e) TGA curves under nitrogen at a heating rate of 10 °C min^−1^. f) Storage modulus (*G*′, circle) and tan *δ* (triangle) determined by DMA at heating rate of 5 °C min^−1^. g) Tensile stress‐strain curves at a stretching rate of 5 mm min^−1^.

As illustrated in **Figure** **2**a, waterborne epoxy vitrimers were prepared using deionized water as the solvent. Initially, *L*‐TA and BDE were dissolved in water to form a colorless solution. Upon removing most of the water at 120 °C, the solution transformed into a yellow gel that exhibited drawable properties at room temperature (Figure S1, Supporting Information). Finally, by placing the yellow gel in a mold on vulcanizer to achieve the high‐viscosity gel at 160 °C (Figure S2, Supporting Information) and hot‐pressing at 160 °C for 5 h, the *L*‐TA‐based waterborne vitrimers were successfully obtained (Figure S3, Supporting Information), which showed high transmittance in the visible range from 380 to 780 nm (Figure S4, Supporting Information). The stoichiometric ratios (*R*) of epoxy to carboxylic acid groups were judiciously adjusted from 1.2 to 1.0 and 0.8, respectively, yielding three waterborne vitrimers denoted as WV_1.2_, WV_1.0,_ and WV_0.8_ (Table S1, Supporting Information). Note that the above process was quite different from traditional epoxy preparation and reported eutectic hardeners,^[^ ^26^, ^27^ ^]^ whereas in this case, the BDE and *L*‐TA could be mixed well by water‐assistance to obtain a homogenous solution, followed by the formation of a yellow gel. This yellow gel behaved like a non‐toxic and storable waterborne polyurethane coating, which could be cured directly to form cross‐linked networks when needed. The method also exhibited broad applicability, as demonstrated by its successful implementation in two control networks: diglycidyl ester of aliphatic cyclo (DGEAC)/*L*‐TA and BDE/butanedioic acid (BA) (Figure S5, Supporting Information). These networks were intentionally established as reference standards to facilitate comprehensive comparison with WV_1.0_. To monitor the curing process, the differential scanning calorimetry (DSC) was applied for WV_1.0_ as a representative (Figure S6, Supporting Information). The BDE/*L*‐TA blend without water‐assistance exhibited a prominent exothermic peak with a maximum at 115 °C, confirming direct cross‐linking reactions between BDE and *L*‐TA. In contrast, the yellow gel exhibited no discernible peaks, suggesting it was in a pre‐cured state rather than a simple physical mixture. The rheological behavior of the yellow gel (WV_1.0_) was further analyzed as shown in Figure S7 (Supporting Information). As the temperature increased, the storage modulus (*G*′) of the yellow gel decreased rapidly before 80 °C but rose sharply after 140 °C, indicating the cross‐linking reactions between BDE and *L*‐TA. Additionally, isothermal rheological test revealed that the sol‐gel transition occurred at 0.43 h at 160 °C, with both storage modulus (*G*′) and loss modulus (*G*′′) stabilizing after 5 h (Figure 2b), indicative of the formation of cross‐linked networks. To further compare the curing behavior of these networks, yellow gel (WV_1.2_, WV_1.0_, WV_0.8_) and BDE/BA gel were analyzed at 120 °C by using a rheometer (Figure S8, Supporting Information). Remarkably, WV_0.8_ exhibited a gelation time of only 2.5 h, significantly shorter than those of WV_1.2_ (5.8 h) and WV_1.0_ (6.9 h), highlighting the acceleration of excess carboxyl groups in the curing reaction. Unlike the yellow gel of WV_1.0_, BDE/BA gel exhibited a distinct opaque white appearance and failed to achieve sol‐gel transition within 10 h. Furthermore, the BDE/BA gel remained in a flowable, uncured state with minimal *G*′ throughout 20‐h testing. This comparative analysis demonstrated that the hydroxyl groups presented in *L*‐TA significantly accelerate the cross‐linking reactions.

The formation of a cross‐linked network was investigated by Fourier transform infrared (FTIR) spectroscopy. As depicted in Figure 2c, the disappearance of characteristic peaks at 908/852 cm^−1^ confirmed the complete consumption of epoxy groups. Additionally, the signal at 1708 cm^−1^, corresponding to the C═O stretching vibration of carboxyl groups in *L*‐TA, shifted to 1740 cm^−1^, indicating the consumption of carboxylic acid and the generation of ester bonds. Notably, for WV_1.2_, the carboxyl groups provided by *L*‐TA were insufficient to fully react with epoxy. In this case, hydroxyl groups also participated in the reaction with epoxy groups, forming ether bonds to provide cross‐linking sites (Figure S9, Supporting Information).^[^ ^28^ ^]^ Meanwhile, the unreacted carboxyl groups remaining in these networks, particularly prominent in WV_0.8_, were exposed and potentially available for further interactions. The cross‐linked networks were further investigated by DSC, where no exothermic peak was observed over 140 °C, reflecting hardly any further reactions occurred after the curing process. Additionally, the glass transition temperature (*T*_g_) was −9, 5, and 9 °C for WV_1.2_, WV_1.0_, and WV_0.8_, respectively (Figure 2d; Table S1, Supporting Information). Gel content tests revealed that all networks maintained high gel contents exceeding 85% in aprotic solvents, validating their cross‐linked network structures (Figure S10 and Table S2, Supporting Information). Note that transesterification reactions (TERs) also occurred during the preparation of vitrimers (under high‐temperature conditions) and even under swelling conditions at room temperature,^[^ ^29^ ^]^ leading to the formation of soluble oligomeric diol and triol fraction in the epoxy vitrimers constructed from bifunctional epoxy and acid (Figure S11, Supporting Information).^[^ ^30^, ^31^ ^]^ To investigate this fraction, the acetone‐soluble extract of cured WV_1.0_ was analyzed by gel permeation chromatography (GPC). The GPC curve showed two distinct peaks with number‐average molecular weight (*M*_n_) of 0.4 and 1.7 kDa (Figure S12, Supporting Information), indicating that the sol fraction is composed of oligomeric species produced by TERs rather than unreacted monomers. Meanwhile, FTIR analysis revealed that the spectrum of the sol fraction closely resembled that of the cured WV_1.0_ network, confirming that these soluble oligomers share the same backbone structure (BDE and *L*‐TA linkages) as the network, albeit with much shorter chains (Figure S13, Supporting Information). Notably, the broad hydroxyl groups stretching band shifted from 3405 cm^−1^ in the network to 3380 cm^−1^ in the sol fraction, suggesting that these oligomers engage in stronger hydrogen bonding than the long polymer chains in the bulk network. Additional evidence was provided by nuclear magnetic resonance (NMR) spectroscopy, which enabled the identification of characteristic signals consistent with diol and triol oligomeric skeletons, as well as the formation of termini primary hydroxyl groups resulting from TERs (Figure S14, Supporting Information). In summary, the following cross‐linking and side reactions take place in these networks: 1) reactions of acid with epoxy to form linear esters; 2) nucleophilic attack of hydroxyl groups on epoxy leading to ether formation; and 3) TERs accompanied by the curing process to generate oligomeric diol, triol, and adjusted yet complicated structures.

The thermal and dynamic mechanical properties of waterborne vitrimers were evaluated through thermogravimetric analysis (TGA) and dynamic mechanical analysis (DMA). As displayed in Figure 2e and Table S1 (Supporting Information), the temperature at 5 wt.% loss (*T*_d5_) for WV_1.2_, WV_1.0_, and WV_0.8_ exceeded 250 °C, demonstrating their good thermal stability. In addition, the mass loss peaks in thermogravimetric derivative (DTG) curves ranged from 298 to 323 °C (Figure S15, Supporting Information), indicating similar structural robustness across the cross‐linked networks. Notably, the networks exhibited minimal water content, with a low weight loss ranging from 0.8% to 1.1% below 150 °C (Figure S16, Supporting Information). The *α* transition of all networks was observed in DMA curves (Figure 2f), while the relatively broad range was attributed to the complexity and heterogeneity of these networks as discussed above.^[^ ^32^ ^]^ The glass transition temperature (*T*_α_) in DMA curves was −6, 8, and 27 °C for WV_1.2_, WV_1.0,_ and WV_0.8_, respectively (Figure 2f and Table S1, Supporting Information), where the tendency was coincidence with *T*_g_ conducted by DSC. The increase in *T*_α_ from WV_1.2_ to WV_0.8_ was attributed to the reduction in flexible chains derived from BDE, as further supported by the rise in storage modulus from 2000 to 5200 MPa at the glass plateau region. Meanwhile, the cross‐linking density (*ν*_e_) of each network was calculated using the rubber‐elasticity theory (*G*′ = 3*ν*_e_ *RT*), yielding values of 38, 136, and 102 mol m^−3^ for WV_1.2_, WV_1.0_, and WV_0.8_, respectively. The WV_1.2_ exhibited the lowest *ν*_e_ due to its relatively low *L*‐TA content, resulting in a longer theoretical distance between cross‐linking sites. As the *L*‐TA content increased, *ν*_e_ rose by over four times from WV_1.2_ to WV_1.0_ but decreased from WV_1.0_ to WV_0.8_. This decline was because of the presence of unreacted excess carboxyl groups on *L*‐TA (Figure S9, Supporting Information), which reduced the number of effective cross‐linking sites. Nevertheless, both higher *ν*_e_ and more rigid chains contributed to the increase in *T*_g_ and *T*_α_. Although the *ν*_e_ of WV_0.8_ was lower than that of WV_1.0_, the augmentation of *L*‐TA dominated in WV_0.8_, causing higher *T*_g_ and *T*_α_. Mechanical properties of WV_1.2_, WV_1.0,_ and WV_0.8_ were further evaluated through tensile tests at room temperature. It should be noted that both the complex network structures and the temperature state within the glass transition range complicate a clear identification of the root cause behind the content variations. However, *L*‐TA demonstrated a highly significant contribution to performance. As the *L*‐TA content increased, the tensile strength improved from 0.60 (WV_1.2_) to 1.18 (WV_1.0_) and 1.68 MPa (WV_0.8_), accompanied by an increase in Young's modulus from 0.57 to 1.41 and 13.06 MPa (Figures 2g; S17, Table S1, Supporting Information). In addition, all systems exhibited elongations exceeding 150%, maintaining their elasticity and suitability for driving deformation. Accordingly, the fracture surfaces of these materials displayed typical ductile fracture characteristics, as evidenced by the dimpled morphology observed in Figure S18 (Supporting Information). By contrast, the rigid network DGEAC/*L*‐TA (*T*_g_ = 95 °C and *T*_α_ = 97 °C) maintained a high *G*′ at room temperature (Figure S19, Supporting Information), significantly constraining its bending deformation capacity. In consequence, the flexible chains introduced by BDE enabled waterborne vitrimers for soft actuator applications.

### 2.2 Dynamic Properties and Water‐Assisted Degradation of Waterborne Vitrimers

**Figure 3:**
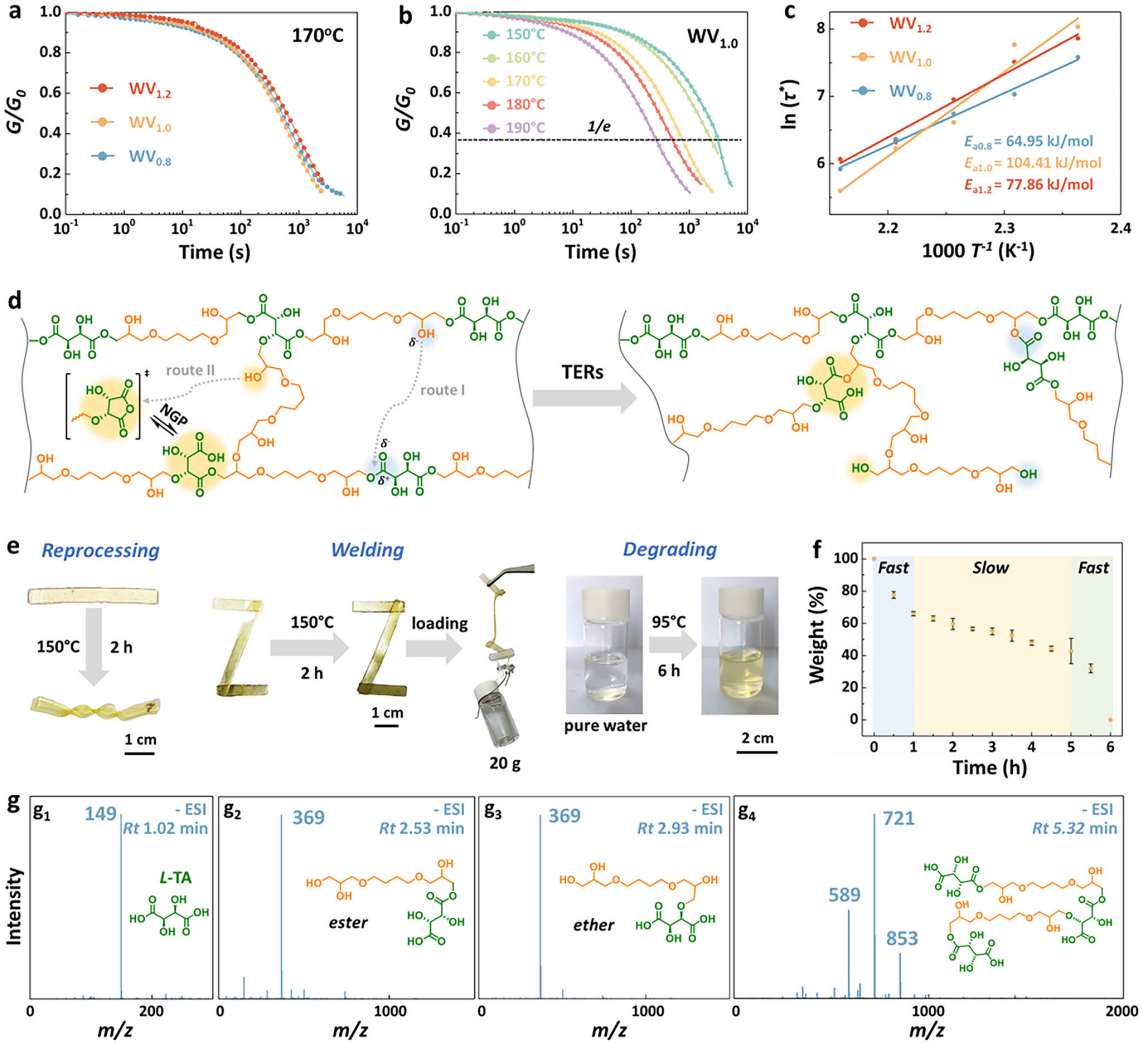
Dynamic properties and water‐assisted degradation of waterborne vitrimers. a) Stress relaxation of waterborne vitrimers at 170 °C by using a rheometer. b) Stress relaxation of WV_1.0_ at temperatures of 150, 160, 170, 180, and 190 °C by using a rheometer. c) Fitting of the experimental relaxation time (*τ* ^*^) to an Arrhenius‐type equation. d) Proposed mechanism for TERs‐induced topological network rearrangements. e) Digital images illustrating the reprocessing, welding, and degrading processes of WV_1.0_. f) Degradation of WV_1.0_ in pure water at 95 °C. Error bars represent the SD derived from triplicate measurements. g) HPLC‐MS spectra of degradation products, showing peaks at (g_1_) *m*/*z* 149 [M ‐ H]^−^, (g_2_) *m*/*z* 369 [M ‐ H]^−^, (g_3_) *m*/*z* 369 [M ‐ H]^−^, and (g_4_) *m*/*z* 853 [M ‐ H]^−^.

To verify the TERs in waterborne vitrimers, we conducted stress relaxation studies on WV_1.2_, WV_1.0_, and WV_0.8_ using a rheometer in shear mode. As illustrated in **Figure** **3**a, all networks demonstrated rapid stress relaxation at 170 °C. For instance, WV_1.0_ exhibited relaxation times (*τ* ^*^) of 3085 s at 150 °C, 2365 s at 160 °C, 746 s at 170 °C, 507 s at 180 °C, and 270 s at 190 °C, where *τ* ^*^ was defined as the time required for the sample to relax to 1/*e* of its initial modulus (*G*/*G*_0_ = 1/*e*) (Figure 3b). The stress relaxation behavior of the material was well‐described by a Maxwell model, which was fitted using an Arrhenius‐type equation (*RTlnτ* ^*^ = *E*_a_ *‐RTlnA*). In this equation, *E*_a_ denotes the activation energy of bond exchange, *T* represents the relaxation temperature, and *R* is the universal gas constant. The calculated *E*_a_ for WV_1.0_ was 104.41 kJ mol^−1^ (Figure 3c, yellow line). Similarly, WV_1.2_ and WV_0.8_ displayed temperature‐dependent stress relaxation behaviors (Figures S20 and S21, Supporting Information), with *E*_a_ values of 77.85 and 64.95 kJ mol^−1^, respectively (Figure 3c, red and blue lines). These *E*_a_ values aligned well with those reported for typical TERs‐based vitrimers (65–150 kJ mol^−1^).^[^ ^1^, ^2^, ^16^, ^20^, ^21^, ^22^, ^23^, ^24^ ^]^ Additionally, the topology freezing temperature (*T*_v_) derived from the stress relaxation was 32, 71, and 49 °C for WV_1.2_, WV_1.0,_ and WV_0.8_, respectively (Figure S22, Supporting Information).^[^ ^14^ ^]^ To elucidate the above stress relaxation behavior, the relaxation induced by slippage of polymer chains was excluded initially, since these waterborne vitrimers displayed negligible creep at 50 °C and exhibited purely elastic deformation with complete recovery (Figure S23, Supporting Information). The rapid stress relaxation observed in waterborne vitrimers can be attributed to the dual pathways of TERs that facilitated topological network rearrangements. As depicted in Figure 3d, both classical TERs (route I) and neighboring group participation (NGP) effect‐assisted TERs (route II) coexisted within the networks. In route I, the abundant hydroxyl groups provided by *L*‐TA attacked ester bonds, generating new hydroxyl and ester groups, thereby promoting network rearrangements. Concurrently, the unique double carboxyl structure in *L*‐TA formed a five‐membered ring anhydride intermediate, which provided the NGP effect for TERs, making them more prone to occur (route II).^[^ ^33^ ^]^ To further demonstrate the unique contribution of *L*‐TA, we replaced it with butanedioic acid (BA) in WV_1.0_ and studied the stress relaxation behavior. As expected, the BDE/BA exhibited a lower glass transition temperature (*T*_g_ = −18 °C and *T*_α_ = −25 °C) compared to WV_1.0_ (*T*_g_ = 5 °C and *T*_α_ = 8 °C), which could facilitate fast stress relaxation (Figure S24, Supporting Information). However, its stress relaxation time (*τ* ^*^) at 170 °C was 3250 s, over four times longer than that of WV_1.0_ (746 s) (Figure S25, Supporting Information). This result confirmed the critical role of *L*‐TA in accelerating stress relaxation, consistent with the proposed mechanism discussed above. Notably, the *L*‐TA‐constructed rigid network (DGEAC/*L*‐TA) also exhibited stress relaxation, with a characteristic *τ* ^*^ of 2078 s, longer than that of WV_1.0_ (Figure S26, Supporting Information). This suggested that the presence of flexible chains contributed to accelerating stress relaxation. Strikingly, despite the DGEAC/*L*‐TA exhibited much higher glass transition temperature (*T*_g_ = 95 °C and *T*_α_ = 97 °C) than that of BDE/BA (*T*_g_ = −18 °C and *T*_α_ = −25 °C), the relatively faster stress relaxation was observed, further underscoring the irreplaceable function of *L*‐TA for network rearrangements. In brief, the multiple hydroxyl groups and NGP effect contributed to the rapid topological rearrangements of waterborne vitrimers. Compared with other reported TERs‐based vitrimers, our waterborne vitrimers exhibited shorter relaxation time than most catalyst‐free TERs‐based vitrimers, and were comparable to some classic vitrimers that utilized external catalysts (Figure S27 and Table S3, Supporting Information),^[^ ^20^, ^22^, ^24^, ^34^, ^35^, ^36^, ^37^, ^38^ ^]^ highlighting the unique contribution of *L*‐TA structure in facilitating fast network rearrangements.

Leveraging the topological network rearrangements, these waterborne vitrimers were able to be easily reprocessed, welded, and degraded. Using WV_1.0_ as an example, a flat sample was successfully reprocessed into a ribbon shape after heating at 150 °C for 2 h (Figure 3e). For welding tests, three flat films were seamlessly fused into an integral “Z” shape under the same conditions, with the new structure capable of supporting a 20 g weight. More importantly, the waterborne vitrimer exhibited rapid and eco‐friendly degradation, completely breaking down in pure water at 95 °C within 6 h, a significant improvement over most TERs‐based vitrimers (Table S4, Supporting Information).^[^ ^22^, ^34^, ^36^, ^39^, ^40^, ^41^, ^42^, ^43^, ^44^, ^45^ ^]^ This green, efficient degradation was attributed to the NGP effect, combined with the high hydrophilicity imparted by *L*‐TA, which facilitated quick swelling and high water absorption. As illustrated in Figure 3f, the degradation exhibited a trend as follows: rapid in the initial 0–1 h, relatively slow between 1 and 4 h, and accelerated from 5 to 6 h. In the initial stage, the vitrimer swelled fleetly as water molecules penetrated the networks. The fragments of linear oligomers dissolved in water and the dangling chains with ester bonds detached from the network by TERs, leading to swift degradation. The degradation rate, dependent on the concentration of linear oligomers and dangling chains with ester bonds, results in a weight loss of 65% at 1 h and 55% at 3 h. Subsequently, the reduction in cross‐linking density and steric hindrance further accelerated degradation. Simultaneously, a large amount of ester bonds on polymer chains were hydrolyzed, and lower molecular weight fragments (linear oligomers) dissolved directly in water. Consequently, the increasing concentration of these oligomers significantly boosted the degradation rate during the final stage (5‐6 h). The degradation products were analyzed using high‐performance liquid chromatography‐mass spectrometry (HPLC‐MS). As shown in Figures 3g, S28 and S29, and Table S5 (Supporting Information), distinct molecular ion peaks were observed at *m*/*z* 149 [M ‐ H]^−^, 369 [M ‐ H]^−^, 853 [M ‐ H]^−^ and 1205 [M ‐ H]^−^corresponding to the molecular formula of C_4_H_6_O_6_, C_14_H_26_O_11_, C_32_H_54_O_26_ and C_46_H_78_O_36_, respectively, which were identified as *L*‐TA and its linear oligomers (Figures 3g_1_–g_4_; S29, Supporting Information). Interestingly, two adjacent peaks (g_2_ and g_3_ in Figure S28, Supporting Information) with the same molecular ion peak (*m*/*z* 369 [M ‐ H]^−^) were detected, representing a pair of isomers linked by *L*‐TA and BDE fragment via ester bond and ether bond, respectively (Figure 3g_2_,g_3_). It should be noted that the hydrolysis and TERs in these vitrimers also occurred at room temperature in water and other protic solvents, with the gel content dropping to as low as 68% in water (Figure S30 and Table S6, Supporting Information). This behavior confirmed that such waterborne vitrimers are suitable for use as disposable degradable materials, thereby reducing the risk of long‐term environmental persistence.

### 2.3 Water‐ and Moisture‐Driven Actuation of Waterborne Vitrimers

**Figure 4:**
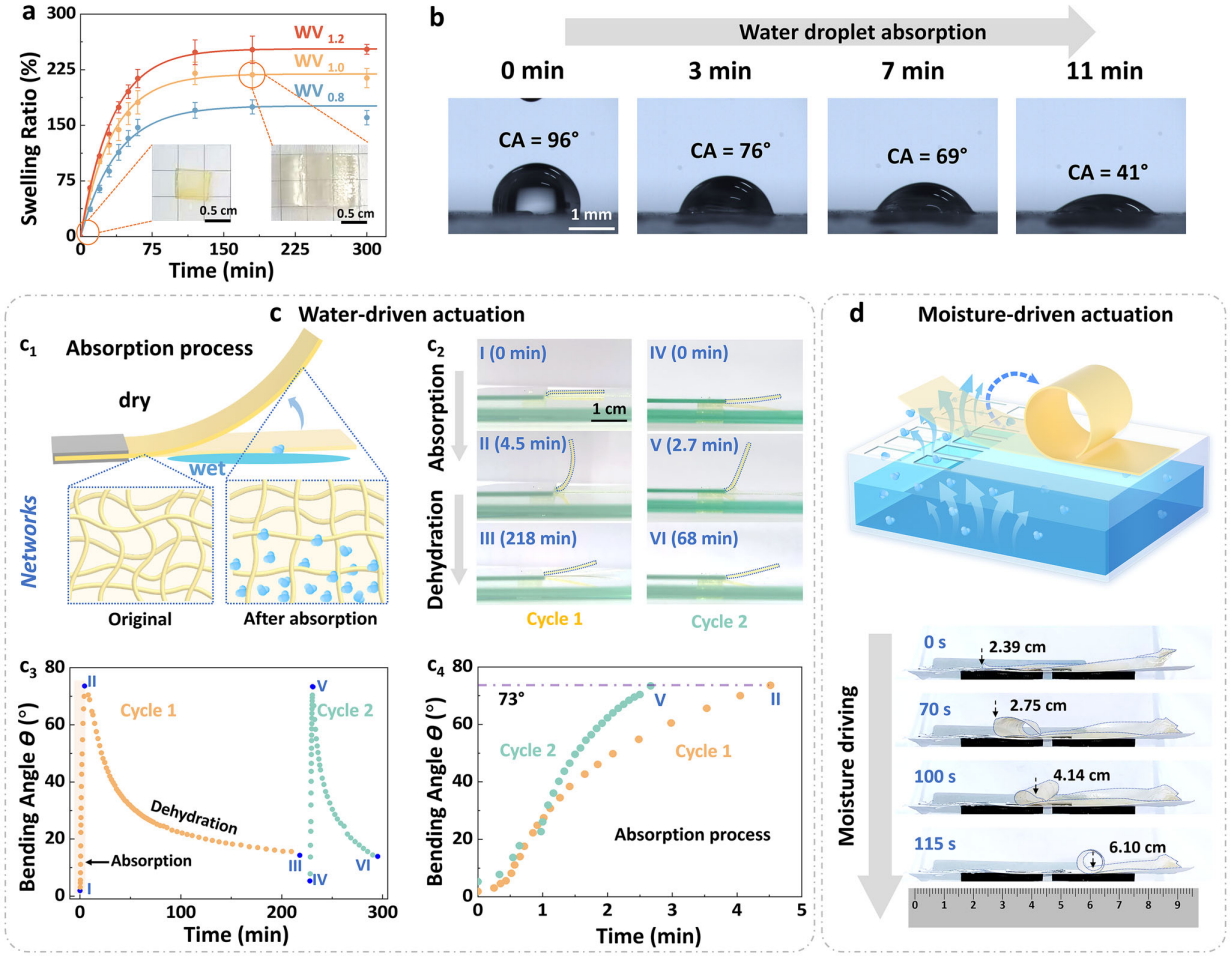
Water‐ and moisture‐driven actuation of waterborne vitrimers. a) Swelling ratio of waterborne vitrimers in water at room temperature. The curves are fitted using the function *R*_t_ = *R*_max_ (1−*e^−A t^*), where *R*_t_ and *R*_max_ represent the real‐time swelling ratio and the maximal swelling ratio, respectively. Inset photos showing WV_1.0_ before and after swelling for 3 h. Error bars represent the SD derived from triplicate measurements. b) Photographs of a water droplet on the surface of WV_1.0_ at different time intervals. c) Water‐driven actuation of a WV_1.0_ film. (c_1_) Schematic illustration of the water absorption process of a WV_1.0_ film placed upon a wet filter paper. (c_2_) Photographs demonstrating the reversible water‐driven behavior of the WV_1.0_ film upon alternating exposure to wet and dry filter papers. (c_3_) Variation of bending angle (𝜃) of the WV_1.0_ film during absorption and dehydration. Points I to II and IV to V correspond to the absorption process (on a wet filter paper), while points II to III and V to VI represent the dehydration process (on a dry filter paper). (c_4_) Comparison of the absorption process between the first and second cycles. d) Schematic diagram and photographs illustrating the moisture‐driven actuation of a WV_1.0_ film. A ruler is included below the photos to measure the movement distance.

The abundant hydroxyl groups embedded in the cross‐linked network endowed the waterborne vitrimers with good hydrophilicity. All of them exhibited swelling behavior in water, following a first‐order kinetic model, achieving high swelling ratios of 248%, 220%, and 170% at 120 min for WV_1.2_, WV_1.0,_ and WV_0.8_, respectively (**Figure** **4**a). The observed decrease in swelling ratio from WV_1.2_ to WV_0.8_ was attributed to the increasing content of *L*‐TA, indicating that the concentration of flexible chains predominantly governed the swelling behavior of these waterborne vitrimers. To elucidate the individual contributions of BDE and *L*‐TA in the observed high water absorption behavior, comparative studies were performed using BDE/BA and DGEAC/*L*‐TA (Figure S31, Supporting Information). The BDE/BA exhibited a relatively low swelling ratio (43%), while the rigid DGEAC/*L*‐TA network demonstrated an even lower swelling ratio (27%), corresponding to an eight‐fold reduction compared with WV_1.0_. These results demonstrated that both structural components played vital but distinct roles for water absorption: (1) abundant hydroxyl groups derived from *L*‐TA substantially enhanced material hydrophilicity, and (2) the flexible aliphatic chains from BDE promoted network expansion through increased free volume and enhanced chain mobility. To further visualize the water absorption process, the contact angle (CA) of a water droplet on the vitrimer surface was measured. For instance, the CA of WV_1.0_ decreased from 96° to 41° within 11 min (Figure 4b), underscoring its good hydrophility and strong water absorption capacity. Capitalizing on these properties, the reversible water‐driven behavior of WV_1.0_ was investigated. As illustrated in Figure 4c_1_, the left side of a WV_1.0_ film was fixed, while the remaining portion was placed upon a wet filter paper to induce water‐driven bending. Once the bending angle 𝜃 reached its maximum, the wet filter paper was replaced with a dry one to facilitate dehydration until the bending angle 𝜃 stabilized. During the water absoprtion process, the bottom of the film swelled rapidly due to water uptake, while the upper part absorbed relatively fewer water molecules, resulting in asymmetric volume expansion and film bending (photo I to II in Figure 4c_2_). In the subsequent dehydration process, water molecules desorbed from the film, and the networks gradually equilibrated through water diffusion (Figure S32, Supporting Information), leading to film recovery (photo III in Figure 4c_2_). This actuation behavior was fully reversible and could be repeated in a second cycle (photos IV to VI in Figure 4c_2_). The bending angle 𝜃 was quantitatively measured to characterize the process (Figure 4c_3_,c_4_). In the first cycle, the 𝜃 of WV_1.0_ increased sharply from 0° to 73° within 4.3 min, demonstrating its good water‐driven responsiveness. However, due to the inherent hydrophilicity of WV_1.0_, the dehydration process was prolonged, with the 𝜃 recovering to 14° after 3 h. Notably, complete desorption of water molecules was not achieved by the end of the first cycle, which in turn resulted in a faster response both in absorption (2.7 min) and dehydration (65 min) in the second cycle (points IV to VI), while maintaining the same maximum 𝜃 of 73° as the first cycle.

Interestingly, similar water‐driven behaviors have been observed in natural plants, where species such as wheat awn, orchid tree seedpods, and pinecones harness daily humidity to promote movement for seed dispersal.^[^ ^46^, ^47^ ^]^ As shown in Figure S33 (Supporting Information), pinecones closed their scales upon hydration and reopened them upon dehydration. Inspired by these natural mechanisms, the reversible water‐driven behavior of WV_1.0_ was successfully replicated to mimic the closing of a flower in a wet environment and its blooming in a dry setting (Figure S34 and Movie S1, Supporting Information). Furthermore, a thin WV_1.0_ film (0.04 mm thickness) was employed to demonstrate actuation under asymmetric moisture conditions. As shown in Figure 4d and Movie S2 (Supporting Information), the film was set on a platform with several holes on the left side, beneath which a water‐filled box provided moisture. The asymmetric moisture distribution induced rapid film crimping within 70 s, followed by a progressive curling that reached 37 mm from left to right within 115 s, demonstrating an average crimp rate of 0.32 mm s^−1^. Despite the response rate of WV_1.0_ may not be competitive compared to that of traditional moisture‐driven actuators (Table S7, Supporting Information), its combination of intrinsic moisture actuation properties, a green yet facile preparation process, and recyclability established it as a unique and promising candidate for sustainable and disposable systems. In summary, leveraging their inherent hydrophilicity, waterborne vitrimers exhibited remarkable water‐ and moisture‐driven actuation capabilities, which open up new possibilities for applications in responsive and adaptive materials.

### 2.4 Moist‐Electric Generation of Waterborne Vitrimers

**Figure 5:**
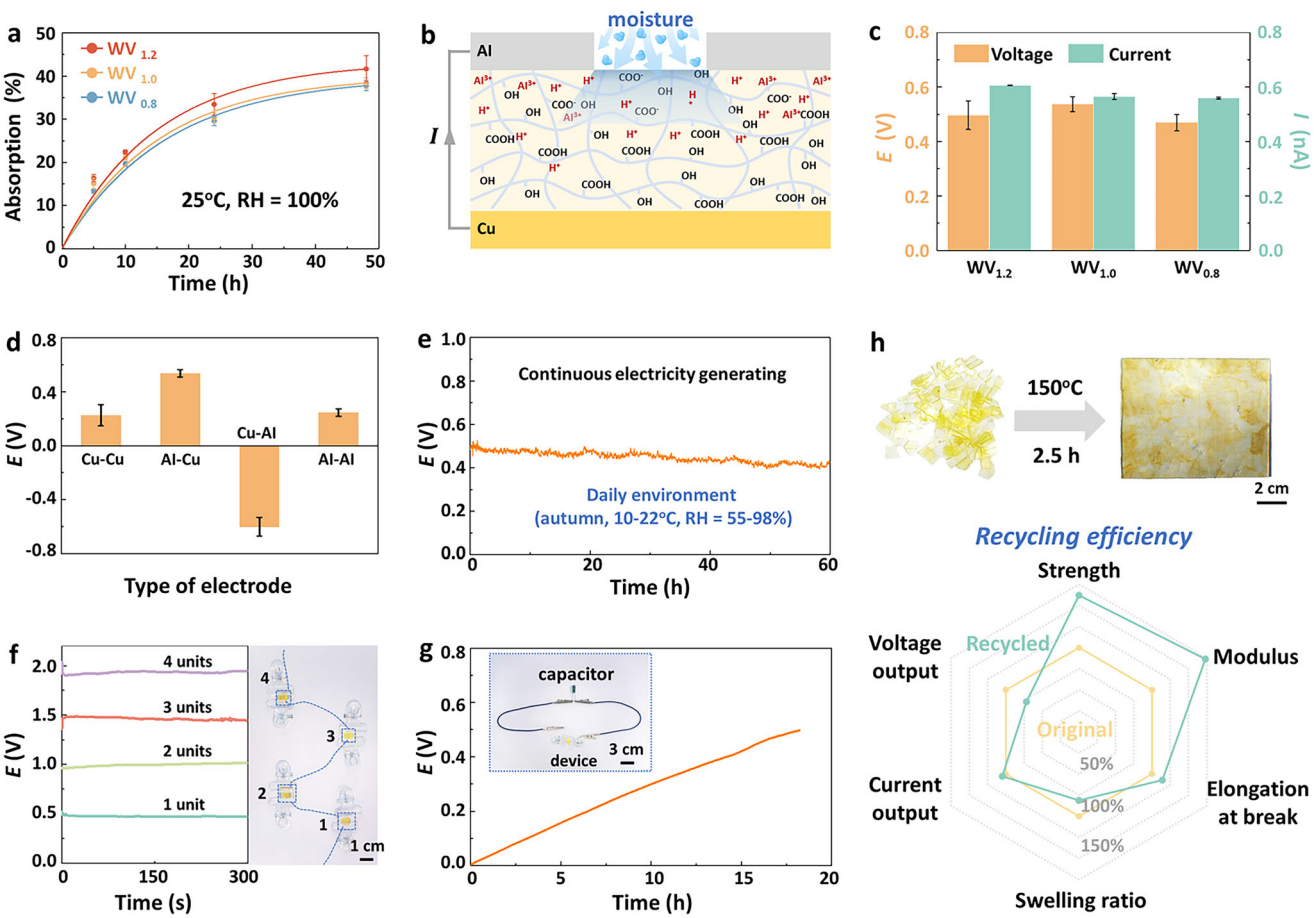
Moist‐electric generation using waterborne vitrimer as a matrix. a) Moisture absorption of waterborne vitrimer at 25 °C under 100% relative humidity (RH). The curves were fitted using the function *R*_t_ = *R*_max_ (1−*e^−A t^*), where *R*_t_ and *R*_max_ represent the real‐time swelling ratio and the maximal swelling ratio, respectively. b) Schematic illustration of the vitrimer‐based moist‐electric generator (WV‐MEG) utilizing Al‐Cu as electrodes. c) Voltage output and current of WV‐MEG under ambient conditions (10 °C, RH = 60%). d) Comparison of voltage outputs of WV_1.0_‐MEG using different electrode configurations. e) Continuous voltage output of WV_1.0_‐MEG in a surrounding environment (10–22 °C and RH = 55–98%) over 60 h. f) Voltage output of the WV_1.0_‐MEG device with 1 to 4 units connected in series. The right panel shows a photograph of four WV_1.0_‐MEG units connected in series, with dotted lines indicating the device and connecting wires. g) Charging of a capacitor using WV_1.0_‐MEG in a surrounding environment (10–16 °C, RH = 60–90%). h) Recycling of WV_1.0_ for reuse as a moist‐electric generator. The top panel showcases digital photographs illustrating the vitrimer recycling process. Bottom panel provides a comparative analysis of the performance between original and recycled waterborne vitrimers, evaluating strength, modulus, elongation at break, swelling ratio, as well as the current and voltage output of their derived MEGs. Error bars in (a, c, d) represent the SD derived from triplicate measurements.

Benefiting from their excellent hydrophility, waterborne vitrimers can continuously absorb moisture at 25 °C under 100% relative humidity (RH), achieving a water content exceeding 37 wt.% of their original mass (**Figure** **5**a). Leveraging this property, a waterborne vitrimer‐based moist‐electric generator (WV‐MEG) was designed, as illustrated in Figure 5b. The device consisted of a WV film (10 mm × 10 mm × 1 mm) sandwiched between a top aluminum (Al) electrode and a bottom copper (Cu) electrode, with a 6.5 mm diameter hole in the top electrode to allow for moisture access. The working mechanism of the WV‐MEG was as follows: Initially, the abundant hydroxyl and carboxyl groups on the surface of the waterborne vitrimer absorbed water molecules, releasing a large number of positively charged H^+^ ions and negatively charged functional groups (─COO^−^) attached to polymer chains. The generated H^+^ ions further reacted with the Al electrode to produce Al^3+^ ions. Subsequently, the accumulation of H⁺ and Al^3^⁺ ions at the top of the vitrimer created a charge concentration gradient, generating a voltage. Finally, as H⁺ and Al^3^⁺ ions migrated from regions of high concentration to low concentration, an electric current was successfully produced.^[^ ^48^, ^49^ ^]^ Experimental results demonstrated that WV_1.2_‐MEG, WV_1.0_‐MEG, and WV_0.8_‐MEG achieved voltage outputs of 0.50, 0.54, and 0.47 V, respectively, along with current outputs of 0.60, 0.56, and 0.56 nA under an ambient environment (10 °C, RH = 60%) (Figure 5c), clearly confirming their moist‐electric generation capabilities. Moreover, the temperature difference (10–22 °C) had minimal impact on voltage output (Figure S35, Supporting Information), indicating that these MEGs can provide stable and continuous output in ambient environments. Notably, the relatively low current output can be attributed to the high electrical resistance of the vitrimer, where the densely cross‐linked insulating networks restricted the free movement of ions. This contrasted sharply with most reported MEGs based on electroactive polymers,^[^ ^50^, ^51^ ^]^ carbon‐based materials,^[^ ^52^, ^53^, ^54^ ^]^ and hydrogels.^[^ ^55^, ^56^, ^57^, ^58^ ^]^ Especially for hydrogel‐derived MEGs, they typically exhibited higher current outputs due to their open‐pore structures and higher water content, facilitating more efficient ion transport.

To investigate the effect of electrode type on voltage output, four electrode configurations were assembled using WV_1.0_ as a representative matrix (Figure S36, Supporting Information). The WV_1.0_‐MEG with an Al‐Cu electrode configuration exhibited the highest voltage (0.63 V), which was three times that of the Cu‐Cu configuration (0.22 V) and four times that of the Al‐Al configuration (0.16 V) (Figure 5d). Interestingly, the Cu‐Al configuration produced a voltage of −0.60 V, further underscoring the crucial role of Al^3^⁺ ions in improving the performance of WV_1.0_‐MEG. The specific arrangement of electrodes significantly influenced the electrochemical reaction interface and ion‐transfer kinetics. When an Al‐Cu electrode combination was used, the disparity in the standard electrode potentials of aluminum and copper promoted more efficient electron transfer, thereby enhancing the voltage output.^[^ ^48^ ^]^ The long‐term moist‐electric generation performance of WV_1.0_‐MEG in ambient conditions was investigated. As shown in Figure 5e, WV_1.0_‐MEG demonstrated a stable and continuous voltage output of 0.5 V over 60 h in a daily environment (autumn, 10–22 °C, RH = 55–98%). Due to the continuous movement of water molecules and positively charged ions from the top to the bottom of the device, the voltage output experienced a slight decline from an initial 0.50 to 0.42 V. The voltage output of the WV‐MEG was comparable with that of MEGs derived from electroactive polymers (0.95 V),^[^ ^51^ ^]^ carbon‐based materials (≈0.50 V),^[^ ^52^, ^53^, ^54^ ^]^ and hydrogels (0.30–0.81 V).^[^ ^56^, ^57^, ^58^ ^]^ Notably, the voltage output could be amplified by connecting multiple WV_1.0_‐MEG in series. The output increased from 0.48 V for a single unit to 1.94 V for four units (Figure 5f). The output of the series‐connected WV_1.0_‐MEG system followed a linear fitting with excellent correlation (Figure S37, Supporting Information), exhibiting a Pearson's coefficient of 0.9998. Furthermore, the generated voltage output was successfully collected and used to charge a capacitor. After 18 h of charging, the capacitor voltage reached 0.49 V (Figure 5g), demonstrating the practical potential of WV_1.0_‐MEG as a power source for an energy storage device. Owing to the inherent flexibility and moisture‐responsive behavior of this vitrimer, it may find important applications as self‐powered soft sensors^[^ ^59^, ^60^, ^61^ ^]^ and iontophoresis patches^[^ ^62^ ^]^ in emerging technologies.

A key advantage of the waterborne vitrimer was its recyclability, allowing it to be reused for MEG applications. As shown in Figure 5h, fragmented waterborne vitrimer could be easily recycled to an integral film through hot pressing at 150 °C for 2.5 h. Tensile tests of the recycled vitrimer demonstrated that the recovery efficiency of strength, Young's modulus, and elongation at break was 162%, 173%, and 114%, respectively (Figures 5h; S38, Supporting Information), highlighting the robust mechanical properties that underpinned its suitability for MEG applications. When the recycled vitrimer was applied for moist‐electric generation, the output voltage decreased from 0.54 to 0.39 V, corresponding to a recovery efficiency of 72%, while the current remained nearly constant (Figures 5h; S39, Supporting Information). This phenomenon may be attributed to post‐curing reactions occurring in WV_1.0_ at high temperatures, which increased the cross‐linking density. While this enhanced the strength and modulus, it also reduced the moisture absorption capacity. Supporting evidence for this can also be found in swelling tests (Figures 5h; S40, Supporting Information), where the swelling ratio of the recycled vitrimer was lower than that of the original material, particularly in the initial stages of swelling (10 min and 20 min). In brief, the multiple hydroxyl groups on the polymer chains of waterborne vitrimers enabled their effective use in a recyclable continuous moist‐electric generator.

## 3 Conclusion

In summary, we have developed a catalyst‐free waterborne vitrimer by curing 1,4‐butanediol diglycidyl ether (BDE) with natural *L*‐tartaric acid (*L*‐TA) through a water‐assisted process. As the *L*‐TA content increased, the strength, Young's modulus, and glass transition temperature (*T*_α_) of the waterborne vitrimer improved significantly, ranging from 0.60 to 1.68 MPa, 0.57 to 13.06 MPa, and −6 to 27 °C, respectively. The incorporation of multiple hydroxyl groups and the neighboring group participation (NGP) effect imparted by *L*‐TA enabled rapid transesterification reactions without an external catalyst, resulting in short stress relaxation times at temperatures of 150–190 °C and allowing reprocessing, welding, and complete degradation in hot water. More importantly, the inherent hydrophilicity facilitated the use of the waterborne vitrimer as a moisture‐driven actuator, capable of curling up to 37 mm within 115 s under asymmetric moisture conditions. Additionally, a moist‐electric generator was fabricated using the waterborne vitrimer as the sandwich layer, which continuously generated a voltage output of 0.42–0.50 V for 60 h and successfully charged a capacitor under ambient conditions. Furthermore, the waterborne vitrimer can be recycled and reused for moist‐electric generation, retaining 72% recovery efficiency in voltage output after recycling. This work presents a green and straightforward strategy for the development and application of hydrophilic epoxy vitrimers, highlighting their potential in sustainable material design and energy harvesting technologies. Considering the high water absorption and water‐degradable properties, these materials might be used as disposable moist‐electric generators for emergency applications, thereby avoiding the instability associated with their use in long‐term hygrothermal environments.

## 4 Experimental Section

### Statistical Analysis

All data were obtained from at least three independent samples and expressed as the mean ± standard deviation (*n* = 3 for polymer characterization; *n* = 5 for mechanical studies).

## Conflict of Interest

The authors declare no conflict of interest.

## Supporting information



Supporting Information

Supplemental Movie 1

Supplemental Movie 2

## Data Availability

The data that support the findings of this study are available from the corresponding author upon reasonable request.
