# Supplementary material for: A Waterborne Epoxy Vitrimer: Enabling Moisture‐Driven Actuation, Continuous Moist‐Electric Generation, and Water‐Assisted Degradation
Source: Adv Sci (Weinh). 2025 Oct 8;13(2):e13579. doi: 10.1002/advs.202513579 (PMC12786341; doi:10.1002/advs.202513579)
Supplement: Supplementary file 1 — Supporting Information [file ADVS-13-e13579-s001.docx]

Supporting Information

A Waterborne Epoxy Vitrimer: Enabling Moisture-Driven Actuation, Continuous Moist-Electric Generation, and Water-Assisted Degradation

*Jianqiao Wu, Yan Zhang, Dongxu Pei, Yaning Ma, Zixuan Wang, Qiuyang Ma, Jianhua Tang, Ousheng Zhang* and Jun Hu**

J. Wu, Y. Zhang, Z. Wang, Q. Ma

College of Materials and Chemical Engineering, Chuzhou University,

Chuzhou, Anhui Province, 239000, China

D. Pei, Y. Ma, J. Hu

Beijing Advanced Innovation Center for Soft Matter Science and Engineering, Beijing University of Chemical Technology

Beijing 100029, China

E-mail: jhu@mail.buct.edu.cn

J. Tang, O. Zhang

Sinopec (Shanghai) Petrochemical Research Institute Co., Ltd

Shanghai 201208, China

E-mail: LtdZhangos.sshy@sinopec.com

Table of contents

**1. Experimental Section**

**2. Supporting Figures**

**Figure S1** Digital photograph captured the precured yellow gel (WV_1.0_) under room temperature conditions.

**Figure S2.** Digital photograph captured the high-viscosity gel (WV_1.0_) on vulcanizer at 160℃ before hot press.

**Figure S3.** Macroscopic photographs of WV_1.2_ (a), WV_1.0_ (b) and WV_0.8_ (c).

**Figure S4.** The optical transmittance of WV_1.2_, WV_1.0_ and WV_0.8_ from 190 to 780 nm.

**Figure S5.** Design and preparation of DGEAC/*L*-TA and BDE/BA.

**Figure S6.** DSC curves of BDE/*L*-TA blend, yellow gel of WV_1.0_, and pure *L*-TA at a heating speed of 5^o^C min^-1^.

**Figure S7.** Storage modulus (*G*′) of WV_1.0_ precursor from 50 to 170℃ by using rheometer at a heating rate of 5℃ min^-1^.

**Figure S8.** Storage modulus (*G*′, green) and loss modulus (*G*′′, orange) of precured gel WV_1.2_ (a), WV_1.0_ (b), WV_0.8_ (c) and BDE/BA gel (d) at 120^o^C by using rheometer.

**Figure S9.** Mechanism of cross-linked networks formation.

**Figure S10.** Gel content test of WV_1.0_ in different aprotic solvents.

**Figure S11.** Proposed mechanism for the generation of soluble oligomers through transesterification reactions (TERs) under high-temperature or swelling conditions.

**Figure S12.** Gel permeation chromatography (GPC) of the sol fraction extracted from WV_1.0_ after immersion in acetone.

**Figure S13.** FTIR spectra of WV_1.0_ network and the sol fraction of WV_1.0_.

**Figure S14.** ^1^H NMR (a) and ^13^C NMR (b) spectra of the sol fraction of WV_1.0_.

**Figure S15.** DTG curves of WV_1.2_, WV_1.0_, and WV_0.8_.

**Figure S16.** TGA curves of waterborne vitrimers ranging from 30 to 150^o^C from Figure 2e.

**Figure S17.** Tensile strength (green), modulus (yellow), and elongation at break (red) of WV_1.2_, WV_1.0_, and WV_0.8_.

**Figure S18.** Scanning electron microscope (SEM) images of WV_1.2_, WV_1.0_ and WV_0.8_.

**Figure S19.** DSC curve (a) and dynamic mechanical properties (b) of DGEAC/*L*-TA.

**Figure S20.** Stress relaxation of WV_1.2_ at 150, 160, 170, 180 and 190℃ by using rheometer.

**Figure S21.** Stress relaxation of WV_0.8_ at 150, 160, 170, 180 and 190℃ by using rheometer.

**Figure S22.** Determination of the topology freezing transition temperature (*T*_v_) of WV_1.2_ (a), WV_1.0_ (b) and WV_0.8_ (c).

**Figure S23.** The creep-recovery of WV_1.2_, WV_1.0_, and WV_0.8_ at 50^o^C.

**Figure S24.** DSC curve (a) and dynamic mechanical properties (b) of BDE/BA.

**Figure S25.** Stress relaxation of DGEAC/*L*-TA at 170^o^C by using rheometer.

**Figure S26.** Stress relaxation of DGEAC/*L*-TA at 170^o^C by using rheometer.

**Figure S27.** The relaxation time of waterborne vitrimers and other TERs-based vitrimers.

**Figure S28.** HPLC of the degradation products include g_1_, g_2_, g_3_, g_4_, and S29 at the retention time of 1.02, 2.53, 2.93, 5.32 and 5.90 min.

**Figure S29.** Peak S29 showed [M - H]^-^ at *m*/*z* 1206 (left), and the proposed chemical structures (right).

**Figure S30.** Gel content tests of WV_1.0_ in water and other protic solvents.

**Figure S31.** Comparison of swelling ratio of DGEAC/*L*-TA, BDE/BA and waterborne vitrimers in water at room temperature for 3 h.

**Figure S32.** Schematic diagram of the reversible water-driven behavior of the WV_1.0_ film upon a dry filter paper.

**Figure S33.** Pinecones placed in dry (left) and wet (right) environments.

**Figure S34.** Photographs of WV_1.0_ film with a thickness of 1 mm upon a wet and dry filter paper from Movie S1.

**Figure S35.** Voltage output of WV-MEG at 10℃ and 22℃ (RH = 60%).

**Figure S36.** Schematic of vitrimer derived moist-electric generator (WV-MEG) by using different electrodes.

**Figure S37.** Linear fitting of the voltage output of WV_1.0_-MEG from Figure 5f versus number connected in series.

**Figure S38.** Comparison of mechanical properties between original and recycled waterborne vitrimers.

**Figure S39.** Comparison of moist-electric generation performance between original and recycled waterborne vitrimers.

**Figure S40.** Swelling ratio of WV_1.0_ before and after recycling.

**3. Supporting Tables**

**Table S1** Constitution, thermal and mechanical properties of WV_1.2_, WV_1.0_ and WV_0.8_

**Table S2** Gel content of WV_1.2_, WV_1.0_ and WV_0.8_ in different aprotic solvents

**Table S3** The relaxation time of waterborne vitrimers and other TERs-based vitrimers

**Table S4** Comparison of the chemical degrading of WV_1.0_ with other reported vitrimers

**Table S5** Proposed chemical structures of potential degradation products and their corresponding fragments in the mass spectrometry (MS) spectrum

**Table S6.** Gel content of WV_1.2_, WV_1.0_ and WV_0.8_ in water and other protic solvents

**Table S7.** Comparison of the of WV_1.0_ with other traditional polymer moisture-driven actuators

**4. Supporting Moives**

**Moive S1** Water-driven actuation of a flower shaped WV_1.0_ film

**Moive S2** Moisture-driven actuation of a WV_1.0_ film

**5. Supporting References**

**1. Experimental section**

**1.1 Chemicals and Materials**

*L*-tartaric acid (*L*-TA, 98%, Energy Chemistry), 1,4-butylene glycol diglycidyl ether (BDE, 98%, Energy Chemical), diglycidyl ester of aliphatic cyclo (DGEAC, Hansort), butanedioic acid (BA, also named as succinic acid, 99.5%, Aladdin Reagent) were used directly without further purifying.

**1.2 Preparation of waterborne vitrimers and controlled epoxy**

*Preparation of waterbone vitrimers:* *L*-TA (20.00 g) was first dissolved in 40 mL deionized water at 80°C, and then BDE was introduced into the solution, with the epoxy/carboxyl group stoichiometric ratios adjusted to 1.2, 1.0, and 0.8 for WV_1.2_, WV_1.0_, and WV_0.8_, respectively. The resulting mixture was heated to 120°C under continuous stirring for 7 h, yielding a light yellow, transparent, and homogeneous precursor solution (Figure S1). This precursor was subsequently transferred into a 100 mm × 80 mm × 1 mm mold maintained at 120°C on a press vulcanizer, followed by heating to 160°C to facilitate water removal and precuring, obtaining a high-viscosity gel (Figure S2). Finally, the mold was cured at 160°C under a pressure of 10 MPa for 5 h, ultimately producing the desired waterborne vitrimers.

*Preparation of DGEAC/L-TA:* *L*-TA (13.00 g) was first dissolved in 20 mL deionized water, and then DGEAC was introduced into the solution, with the epoxy/carboxyl group stoichiometric ratios of 1.0. The resulting mixture was heated to 130°C under continuous stirring for 2 h, yielding a light, transparent, and homogeneous precursor solution with high viscosity. This precursor was subsequently transferred into an oven at 130°C for 10 h to remove water. Then it was placed at a 100 mm × 80 mm × 1 mm mold maintained at 180°C on a press vulcanizer and cured at 180°C for 5 h, obtaining DGEAC/*L*-TA fragments. Finally, the DGEAC/*L*-TA fragments were heated at 180°C for another 2 h and followed with 200°C for 2 h without mold, obtaining DGEAC/*L*-TA materials.

*Preparation of BDE/BA:* BA (15.00 g) was first dissolved in 35 mL deionized water, and then BDE was introduced into the solution at 80°C, with the epoxy/carboxyl group stoichiometric ratios of 1.0. The resulting mixture was heated to 140°C under continuous stirring for 7 h, yielding a light yellow, transparent, and homogeneous precursor solution. This precursor was subsequently transferred into a 100 mm × 80 mm × 1 mm mold maintained at 180°C on a press vulcanizer, followed by heating for 2 h to facilitate water removal and precuring. Finally, the mold was cured at 180°C under a pressure of 10 tons for 4 h, obtaining BDE/BA.

**1.3 Fabrication of waterborne vitrimer moist-electric generator (WV-MEG)**

The waterborne vitrimer film was precisely sectioned into 10 mm × 10 mm × 1 mm specimens using a precision cutting tool. These specimens were then carefully sandwiched between two metallic electrodes. To facilitate moisture exposure, a 6.5 mm diameter aperture was precisely machined into each upper electrode using a laser cutting system. The complete assembly was securely fastened using two insulated plastic clips to ensure proper electrical contact and mechanical stability during operation.

**1.4 Morphology observation of waterborne vitrimer**

The fracture surfaces of WV_1.2_, WV_1.0_, and WV_0.8_ were examined using a scanning electron microscope (SEM, JEOL, JSM-6510LV, Japan) at an acceleration voltage of 20 kV.

**1.5 Optical transmittance test**

The optical transmittance of WV_1.2_, WV_1.0_ and WV_0.8_ samples was characterized using a Cary UV-Vis spectrophotometer (Agilent Technologies, formerly Varian, USA) across the wavelength range of 190-780 nm. With a consistent sample thickness of 1 mm, each specimen was directly mounted on the cuvette's exterior surface, utilizing its inherent adhesive characteristics for optical measurement. testing.

**1.6 Differential scanning calorimeter (DSC) test**

A differential scanning calorimeter (DSC3, Mettler Toledo, Switzerland) was applied for all the samples with nitrogen purge of 50 mL min^-1^. For BDE/*L*-TA blend, yellow gel of WV_1.0_, and pure *L*-TA, temperature range from RT to 200°C, and heating rate of 5°C min^-1^. The BDE/*L*-TA blend was prepared by mixing BDE with *L*-TA directly without water-assistance process. The stoichiometric ratio of epoxy/carboxyl groups was 1.0. For the cured WV_1.2_, WV_1.0_, WV_0.8_ materials, temperature range from -50-200°C, and heating rate of 10°C min^-1^. For the DGEAC/*L*-TA and BDE/BA materials, the started temperature was set at RT and -70°C, respectively. The samples were approximately 5~7 mg and sealed in aluminum sample crucibles.

**1.7 Rheological test**

For rheological analysis, disc-shaped specimens with a standardized diameter of 25 mm were prepared. The rheological properties of the precured yellow gel were investigated using a Discovery Hybrid Rheometer-2 (DHR-2, TA Instruments, USA) under dynamic temperature sweep conditions. The temperature was systematically increased from 50°C to 160°C at a controlled heating rate of 5°C min⁻¹. Isothermal rheological measurements were subsequently conducted by maintaining the precured gel at 160°C for 7 h to evaluate its time-dependent viscoelastic behavior. For the comparison of WV_1.2_, WV_1.0_, WV_0.8_, and BDE/BA precured gel, all the samples were stirred and preheated at 120°C by using water-assisted process for 7 h before tests, then measuring at 120°C to detect the sol-gel transition.

**1.8 Fourier transform infrared spectroscopy (FTIR) analysis**

FTIR spectra were conducted using a Nicolet 6700 FTIR spectrophotometer (Thermo Fisher Scientific, USA). For sample preparation, approximately 100 mg of each powdered sample was homogenously mixed with spectroscopic-grade potassium bromide (KBr) at a ratio of 1:100 (sample:KBr) in an agate mortar. The homogeneous mixture was then compressed under vacuum to form transparent pellets. Spectral acquisition was performed in transmission mode over the wavenumber range of 4000-400 cm^-1^.

**1.9 Gel content test**

The solvent in gel content tests was divided to aprotic solvent and protic solvent. Dichloromethane (DCM), ethyl acetate (EtOAc), tetrahydrofuran (THF), acetone (ACE), acetonitrile, N-vinyl-2-pyrrolidone (NVP) and dimethyl sulfoxide (DMSO) were selected as aprotic solvent. Water, methanol (MeOH), acetic acid (HAc), and ethanol (EtOH) were selected as protic solvent.

The dry sample (~30 mg, *m*_0_) was immersed for 25 h in different solvent. After 25 h, the samples were dried in a vacuum oven until the solvent removing and then weighted (*m*_1_). The dry condition was 80°C/15 h for all the samples, while additional evacuating process by using an oil pump was applied for NVP and DMSO removal. All samples were dried to constant mass. The gel content was calculated according to the following equation (S1):

$Gel contents (\%)=\frac{m_{1}}{m_{0}} \times100\%$ (S1)

The sol fraction of WV_1.0_ was collected after immersing WV_1.0_ materials in acetone for 25 h. Subsequently, the sol fraction was evaporated to dryness by rotary evaporation, resulting light yellow viscous gel-like liquid. The resulted yellow liquid was applied for further study as following:

*Gel permeation chromatography (GPC) analysis for sol fraction of WV_1.0_*: GPC analysis was performed using an Agilent 1260 Infinity II (Agilent Technologies, USA). Approximately 17 mg (yellow liquid) of the sol fraction was dissolved in THF (3 mL) for measurement. An injection volume of 40 μL was introduced *via* a 20 μL quantitative loop. The mobile phase consisted of THF, delivered at a constant flow rate of 1.0 mL‧min^-1^. Separation was carried out using a 300 mm column maintained at 40 °C. A differential refractive index (DRI) detector, also kept at 40 °C, was used for signal detection. Molecular weight calibration was performed using two polystyrene standards: a high molecular weight standard (*M*_w_ = 119,082 g‧mol^-1^; retention time = 6.75 min) and a low molecular weight standard (*M*_w_ = 1,759 g‧mol^-1^; retention time = 8.33 min).

*Attenuated total reflectance Fourier transform infrared spectroscopy (ATR-FTIR) analysis for sol fraction of WV_1.0_*: ATR-FTIR analysis was performed utilizing the SHIMADZU IRXross instrument, which samples were analyzed directly by placing them on the diamond crystal of the ATR accessory. For WV_1.0_ network, the sample sized as 20 mm × 20 mm × 1 mm.

*Nuclear magnetic resonance (NMR) analysis for* *sol fraction of WV_1.0_*: ¹H NMR and ¹³C NMR spectrum were recorded on a Bruker 600 MHz spectrometer (Bruker, Germany) at room temperature. The sol fraction of WV_1.0_ was dissolved in DMSO-d₆. Given the highly hygroscopic nature of the sample, the sample was heated at 100°C with the cap open for 30 min prior to data acquisition to remove residual moisture. The ¹H NMR spectrum was acquired using standard parameters, while the ^13^C NMR spectrum was acquired with a prolonged acquisition time of 1.5 h to ensure an acceptable signal-to-noise ratio.

**1.10 Thermogravimetric analysis (TGA)**

The thermal stability was measured by using a thermogravimetric analyzer (SDT-Q600, TA Instruments, USA) under nitrogen from room temperature to 700°C at a heating rate of 10°C min^-1^.

**1.11 Tensile test**

Tensile stress-strain curves were obtained by using a universal testing machine (CMT4304, MTS Systems Corporation, USA) according to ASTM D882 standard. Dog-bone samples were used for the test. The crosshead speed was 5 mm min^-1^, and the gauge length was 17 ± 2 mm. The average value with error bar of all mechanical data was obtained after 5 times tests for each sample. Mechanical properties were determined through tensile testing of a minimum of five replicate specimens. The results were presented as mean values with corresponding standard deviations to ensure statistical reliability.

**1.12 Dynamic mechanical analysis (DMA)**

Dynamic mechanical properties were measured by using a dynamic mechanical analyzer (DMA1, Mettler Toledo, Switzerland) in tension mode. The sample with a dimension of 30 mm × 5 mm × 1 mm at a heating rate of 5°C min^-1^. The scanning range for waterborne vitrimers was from -35 to 150°C, for BDE/BA was -40 to 150°C, and for DGEAC/*L*-TA was RT to 200°C. The amplitude was set at 10 μm and the frequency was 1 Hz. The crosslinking density (*ν*_e_) of cross-linked vitrimers was calculated using equation (S2):

$G'=3\nu_{e}RT$ (S2)

where *ν*_e_ represents the cross-linking density of the material, *G*′ denotes the storage modulus at temperature *T*, *T* is a temperature of *T*_α_ + 50°C, and *R* is the universal gas constant (8.314 J mol^-1^ K^-1^).^[S1]^ Both of the *G*′ and *T*_α_ can be obtained from DMA tests (Figure 2f). The value of *G*′ was 0.3031, 1.1198 and 0.8931 MPa for WV_1.2_, WV_1.0_ and WV_0.8_, respectively, while the *T*_α_ was -6, 8, and 27℃ for WV_1.2_, WV_1.0_ and WV_0.8_, respectively. Consequently, *ν*_e_ was 38, 136 and 102 mol m^-3^ for WV_1.2_, WV_1.0_ and WV_0.8_, respectively.

**1.13 Creep-recovery test**

Creep-recovery was measured by using a dynamic mechanical analyzer (DMA1, Mettler Toledo, Switzerland) in tension mode at 50°C. The sample dimensions were 30 mm × 5 mm × 1 mm, with an effective gauge length of 10 mm. To ensure clear observation of the initial response, the sample was held at a stress-free state for an equilibration period (30-300 s) prior to testing. Subsequently, a constant stress of 0.2 MPa was applied for 30 min (creep phase), followed by stress removal for an additional 30 min (recovery phase).

**1.14 Stress relaxation test**

Stress relaxation was measured by using a Discovery Hybrid Rheometer-2 (DHR-2, TA Instruments, USA). For the tests by rheometer, the sample size was a 25 mm diameter disc. Samples were clamped at 50°C and heated to the ordered temperature. After that, an instantaneous strain of 5% was forced to the material. The stress and modulus were detected until it achieved equilibrium. The fitting lines in Figure 3c was obtained from Arrhenius law as followed equations (S3, S4 and S5):

For WV_1.2_

$y=7.81x-10.92$ (*r* = 0.9923) (S3)

For WV_1.0_

$y=12.56x-21.52$ (*r* = 0.9827) (S4)

For WV_0.8_

$y=9.37x-14.21$ (*r* = 0.9961) (S5)

The Maxwell relation (equation S6) below is used to calculate *T*_v_ which is defined as the point at the viscosity *ƞ* = 10^12^ Pa s^-1^.^[S2,S3]^ The shear modulus *G* was estimated from storage modulus (*G'*) as measured by DMA with the relation. *G'* of the sample was calculated as the average modulus at temperature ranging from 150 to 180°C from the modulus-temperature curves (Figure 2f). The average plateau modulus of the sample is 0.20, 1.02 and 0.90 MPa for WV_1.2_, WV_1.0_, and WV_0.8_, respectively. The Poisson's ratio ν is chosen as the value 0.5, which is usually used for rubbers. The calculation of shear modulus *G* was shown as following equation S7:

$ƞ=G\tau^{*}$ (S6)

$G=\frac{E'}{2(1+\nu)}$ (S7)

Consequently, the *T*_v_ of WV_1.2_, WV_1.0_, and WV_0.8_ was 32, 71, and 49°C, respectively (**Figure S18**).

**1.15 Reprocessing test**

A flat sample (WV_1.0_) was coiled into a spiral configuration with both ends fixed. Subsequently, the fixed spiral sample was placed in an oven at 150°C for 2 h. The constrained spiral sample was then subjected to thermal treatment in an oven at 150°C for 2 h. Following the heat treatment, the constraints were removed, and the resultant spiral morphology was documented using digital imaging.

**1.16 Welding test**

Three individual components (WV_1.0_) were meticulously arranged into a distinct “Z” configuration. This assembled structure was subsequently subjected to thermal processing on a plate vulcanizer maintained at 150°C for 2 h. During this process, the top plate of the vulcanizer was in direct contact with the structure to ensure uniform heating. Following the thermal treatment, the resulting welded assembly was documented through digital imaging. To assess the welding integrity, a 20 g mass was applied as a test load.

**1.17 Chemical degradation test**

Initially, pure water was brought to boiling. Approximately 70 mg of the rectangular sample (WV_1.0_) was then immersed in 10 mL of the water (maintained at 95°C) within a sample bottle. The bottle was subsequently transferred immediately to a preheated oven stabilized at 95°C to maintain constant temperature conditions. By weighting the dry sample at different time, the degradation behavior were obtained. All data was obtained through three replicate specimens. The results were presented as mean values with corresponding standard deviations to ensure statistical reliability.

**1.18 High-performance liquid chromatography-mass spectrometry (HPLC-MS)**

The HPLC-MS analysis was performed using an Agilent 1200 series system coupled with a 6540 Accurate-Mass Q-TOF mass spectrometer (Agilent Technologies, USA). Mass spectrometric detection was conducted in negative ionization mode with an electrospray ionization (ESI) source. For sample preparation, the degradation products were dissolved in acetonitrile to achieve a final concentration of 10 mg/mL. Chromatographic separation was achieved using an Agilent Eclipse XDB-C18 column (4.6 × 250 mm, 5 μm particle size) maintained at room temperature. The mobile phase consisted of (A) ultrapure water and (B) formic acid, delivered at a constant flow rate of 0.3 mL min^-1^. The gradient elution program was as follows: 0-2 min, 5% B; 2-20 min, linear increase from 5% to 100% B. The mass spectrometry parameters were optimized as follows: drying gas temperature, 300°C; drying gas flow rate, 11 L/min; nebulizer pressure, 35 psig; capillary voltage, 3500 V; fragmentor voltage, 125 V. Full scan mass spectra were acquired in the range of *m*/*z* 0-2000.

**1.19 Swelling and mositure absorption test**

The swelling ratio was measured by immerging the sample (~100 mg, *m*_0_) in water at room temperature. When the time reached 10, 20, 30, 40, 50, 60, 120, 180 and 300 min, the sample was taken out of the solution. The excessive surface solvent was removed with a filter paper, and the swollen sample was weighted (*m*_t_). To enable direct comparison, swelling photographs taken before and after tests selected sample area of approximately 0.5 mm × 0.5 mm. Similarly, the moisture absorption test was obtained by placing the sample (10 mm × 10 mm × 1 mm, *m*_0_) in 100% RH at 25°C and measuring the sample mass (*m*_t_) at different time periods. The swelling ratio and moisture absorption ratio was calculated according to the following equation (S8):

$Swelling ratio (\%)=\frac{m_{t}}{m_{o}}\times100\%$ (S8)

The lines in Figure 4a and Figure 5a are the fit as the following equation (S9):

$R_{t}=R_{max} (1-e^{-A t})$ (S9)

where *R*_t_ is the real-time swelling ratio of vitrimer, *t* (min) is the swelling time, *R*_max_ is the maximum swelling ratio of each vitrimer, *A* is the constant of swelling rate. All data was obtained through three replicate specimens. The results were presented as mean values with corresponding standard deviations to ensure statistical reliability.

Consequently, the swelling ratio (**Figure 4a**) of each vitrimer followed equation S10 to S12:

For WV_1.2_

$R_{t}=176.28\% (1-e^{-\frac{t}{37.91}})$ (S10)

For WV_1.0_

$R_{t}=219.21\% (1-e^{-\frac{t}{33.57}})$ (S11)

For WV_0.8_

$R_{t}=253.10\% (1-e^{-\frac{t}{33.37}})$ (S12)

Similarly, the moisture absorption (**Figure 5a**) of each vitrimer followed equation S13 to S15:

For WV_1.2_

$R_{t}=39.88\% (1-e^{-\frac{t}{16.25}})$ (S13)

For WV_1.0_

$R_{t}=40.09\% (1-e^{-\frac{t}{15.22}})$ (S14)

For WV_0.8_

$R_{t}=43.36\% (1-e^{-\frac{t}{10.28}})$ (S15)

**1.20 Contact angle tests**

The specimen was precisely sectioned into 1 cm × 1 cm square pieces. The prepared sample was then mounted on the contact angle measurement stage. A precisely calibrated 50 μL microsyringe was employed to dispense deionized water droplets onto the sample surface. The droplet profile was captured using a high-resolution digital imaging system for subsequent contact angle analysis.

**1.21 Water-driven actuation test**

A WV_1.0_ film (50 mm × 5 mm × 1 mm) was fixed at its left end and placed in contact with a wet filter paper substrate. The film underwent hydration-induced bending until reaching its maximum bending angle (𝜃_max_). Subsequently, the wet filter paper was replaced with a dry counterpart to initiate the dehydration recovery process, during which the bending angle gradually decreased until reaching a stable equilibrium state. The complete hydration-dehydration actuation cycle was digitally recorded using a camera for subsequent kinematic analysis. The temporal evolution of the bending angle was quantitatively analyzed using motion analysis software (Tracker).

In **Movie S1**, the specimen with a thickness of 1 mm was cut into a floral morphology. The shaped sample was then positioned on a moisture-saturated filter paper substrate to initiate the hydration process. Following complete hydration, the saturated filter paper was systematically replaced with a desiccated counterpart to facilitate controlled dehydration, enabling the observation of reversible shape transformation.

**1.22 Moisture-driven actuation test**

A thin film with a thickness of 40 μm was initially fabricated. The prepared film was then mounted on a perforated platform without additional fixation, where the left section was positioned above a water reservoir to establish a controlled humidity gradient. The moisture-responsive actuation process was digitally captured using a camera, as documented in **Movie S2**.

**1.23 WV-MEG electrical performance test**

Conductive wires were connected to the WV-MEG and potentiostat (CHI660E, China). Select either the OCPT mode or the i-t mode on the electrochemical workstation and carry out the tests in an environment with stable temperature and humidity. The environments for this experiment are 10℃, RH = 60% and 22℃, RH = 60%. The relevant test results are given by the potentiostat. To avoid any interference from static electricity, all samples were short-circuited before testing. All data was obtained through a minimum of three replicate specimens. The results were presented as mean values with corresponding standard deviations to ensure statistical reliability.

**1.24 Generator series connection test**

The WV_1.0_-MEG was connected in series configurations (ranging from one to four units) by using copper wires, interconnects before interfacing with the electrochemical workstation. For multiple unit measurements, the electrical connections were sequentially established from the top aluminum (Al) electrode of one unit to the bottom copper (Cu) electrode of the adjacent unit, repeating this pattern to create a continuous series circuit for voltage amplification. The voltage was measured by using an electrochemical workstation in the open circuit potential-time (OCPT) mode. Prior to testing, the circuit was short-circuited to ensure proper initialization. The sampling interval was fixed at 0.1 s throughout all measurements. Ambient environment temperature was 22℃ and the RH = 60%.

**1.25 Capacitor charging test**

A 220 μF capacitor (maximum voltage of 10 V) was connected to the WV_1.0_-MEG (inset photo in Figure 5g). Meanwhile, the electrodes of both ends of the capacitor were connected to an electrochemical workstation in parallel configurations. The voltage of capacitor was measured by using the electrochemical workstation in OCPT mode. Prior to testing, the circuit was short-circuited to ensure proper initialization. The sampling interval was fixed at 10 s throughout all measurements. Ambient environment temperature was 10-16℃ and the RH = 60-90%.

**1.26 Physical recycling**

The sample pieces (WV_1.0_) were gathered and put into a mold on a press vulcanizer at 150°C for 2.5 h at 10 tons pressure to form a new film. The new film was used to prepare dumbbell-shaped samples and quadrate sample with similar size to original ones for tensile test, swelling test and MEG preparation. All the experiments for recycled WV_1.0_ was performed at the same condition with the original WV_1.0_. The data of tensile tests was obtained through five replicate specimens, while the data of swelling and MEG tests was performed through three replicate specimens. The results were presented as mean values with corresponding standard deviations to ensure statistical reliability. The recovery efficiency was calculated as follows:

$R_{e}=\frac{X_{r}}{X_{o}}$ $\times100\%$ (S16)

where *R*_e_ represents the recovery efficiency, *X*_r_ denotes the test value of recycled sample, and *X*_o_ corresponds to the test value of original one. Both values are averaged to ensure accuracy. For the swelling tests, the final *R*_e_ is calculated as the average of the recovery effeciency measured at 10, 20, 30, 40, 50 and 60 min.

**S2. Supporting Figures**


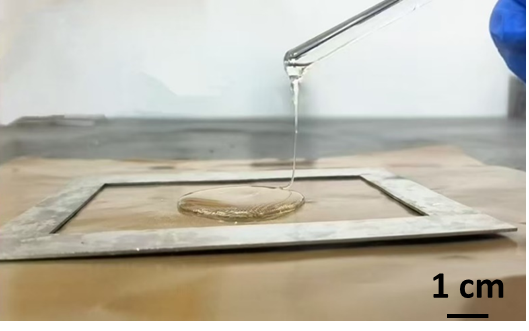


**Figure S1.** Digital photograph captured the precured yellow gel (WV_1.0_) under room temperature conditions. The gel, exhibiting a sticky state, was positioned on a release paper, and a glass rod was employed to dip the gel.


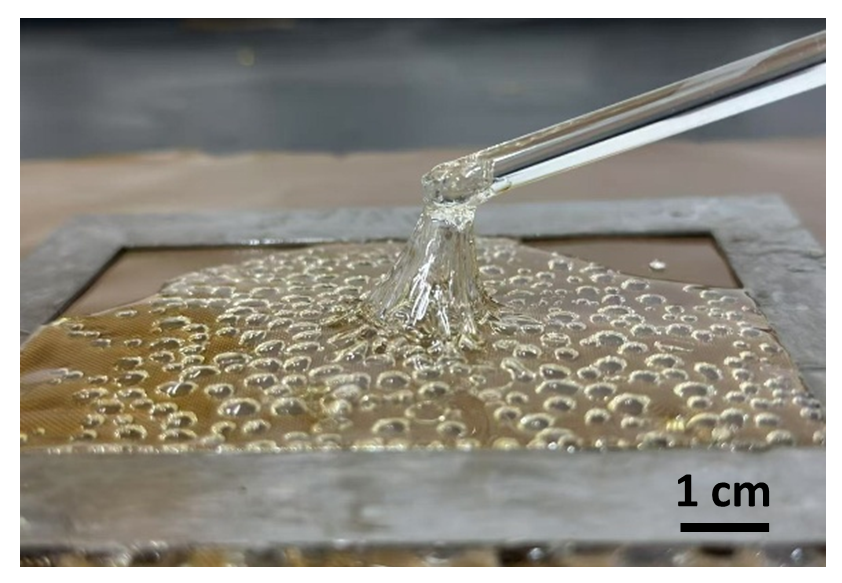


**Figure S2.** Digital photograph captured the high-viscosity gel (WV_1.0_) on vulcanizer at 160℃ before hot press. The viscosity of gel is so high that the glass rod can hardly pick it up.


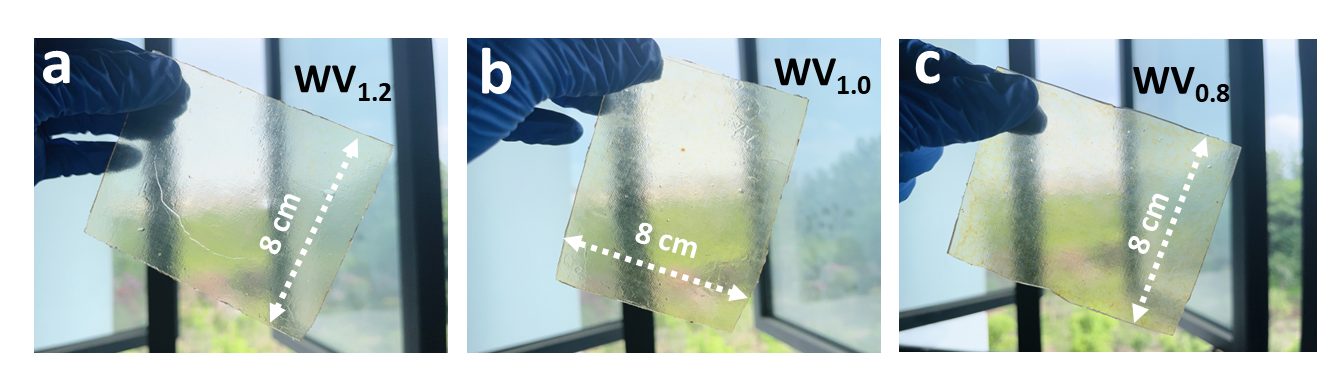


**Figure S3.** Macroscopic photographs of WV_1.2_ (a), WV_1.0_ (b) and WV_0.8_ (c). The photographs confirmed the absence of microstructural defects, including voids or bubbles, across all compositions.


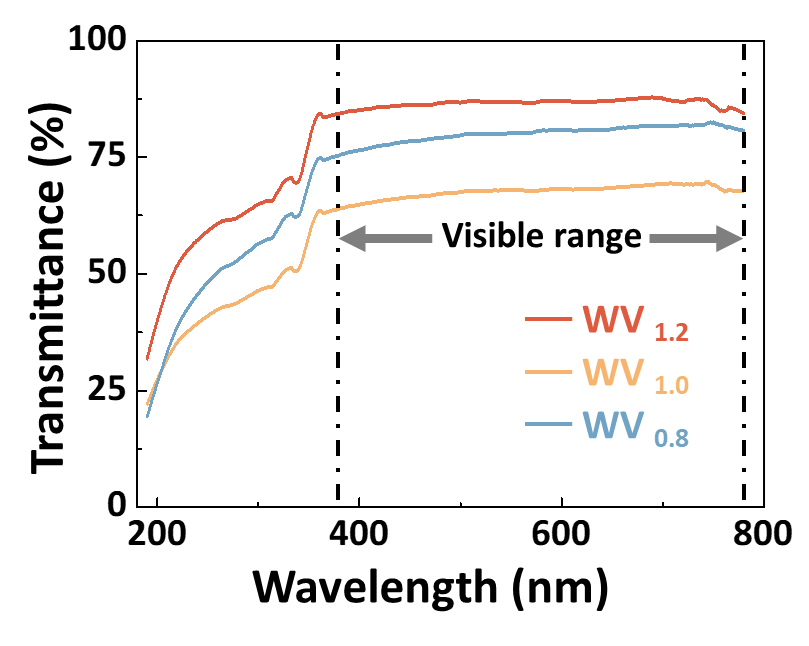


**Figure S4.** The optical transmittance of WV_1.2_, WV_1.0_ and WV_0.8_ from 190 to 780 nm. The average transmittance of WV_1.2_, WV_1.0_ and WV_0.8_ from visible range (380 to 780 nm) was 87%, 68% and 80%, respectively. The sample thickness was 1 mm.


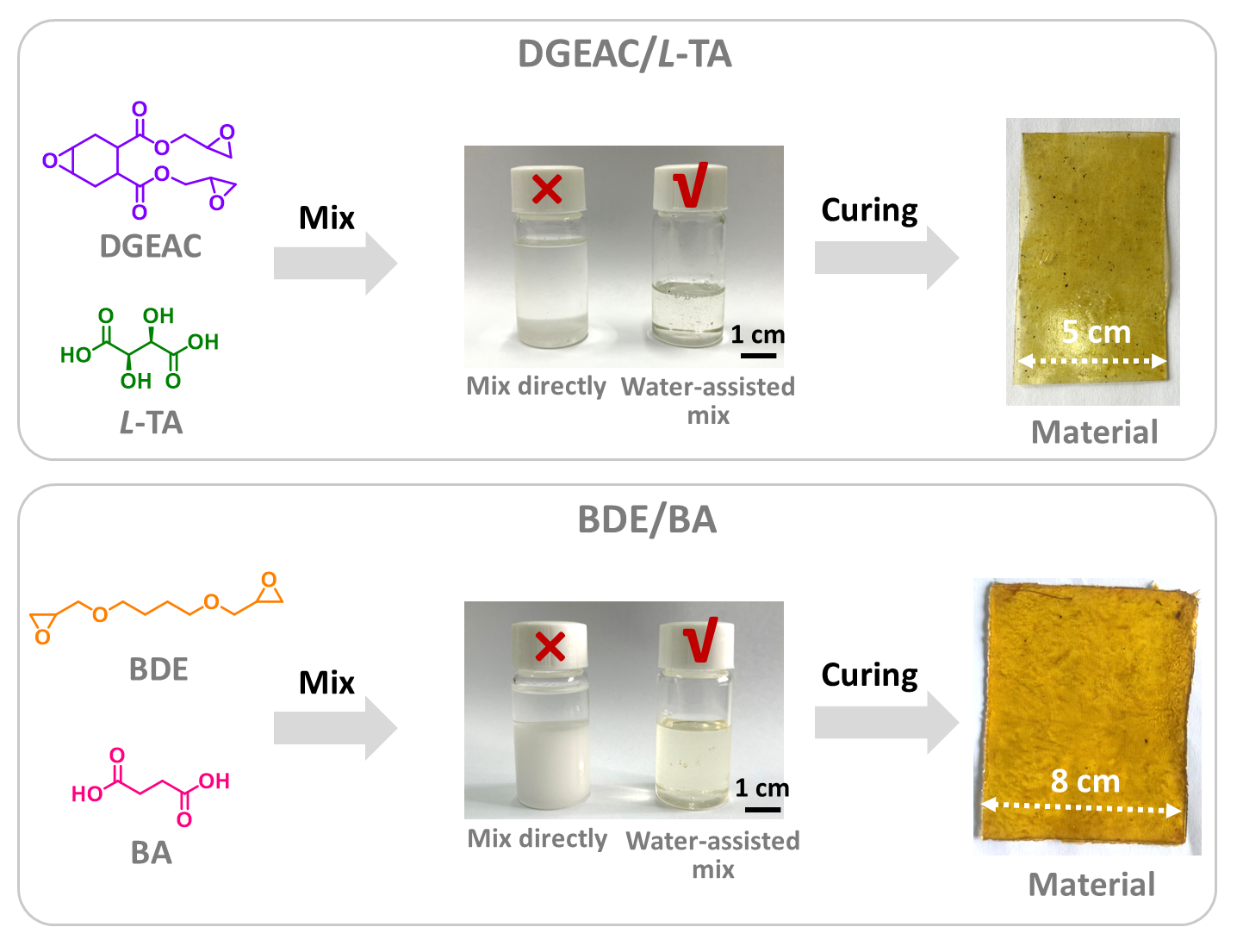


**Figure S5.** Design and preparation of DGEAC/*L*-TA and BDE/BA. Water-assisted mixing process similar to waterborne vitrimers preparation was used for fabricating DGEAC/*L*-TA and BDE/BA, obtaining transparent materials.


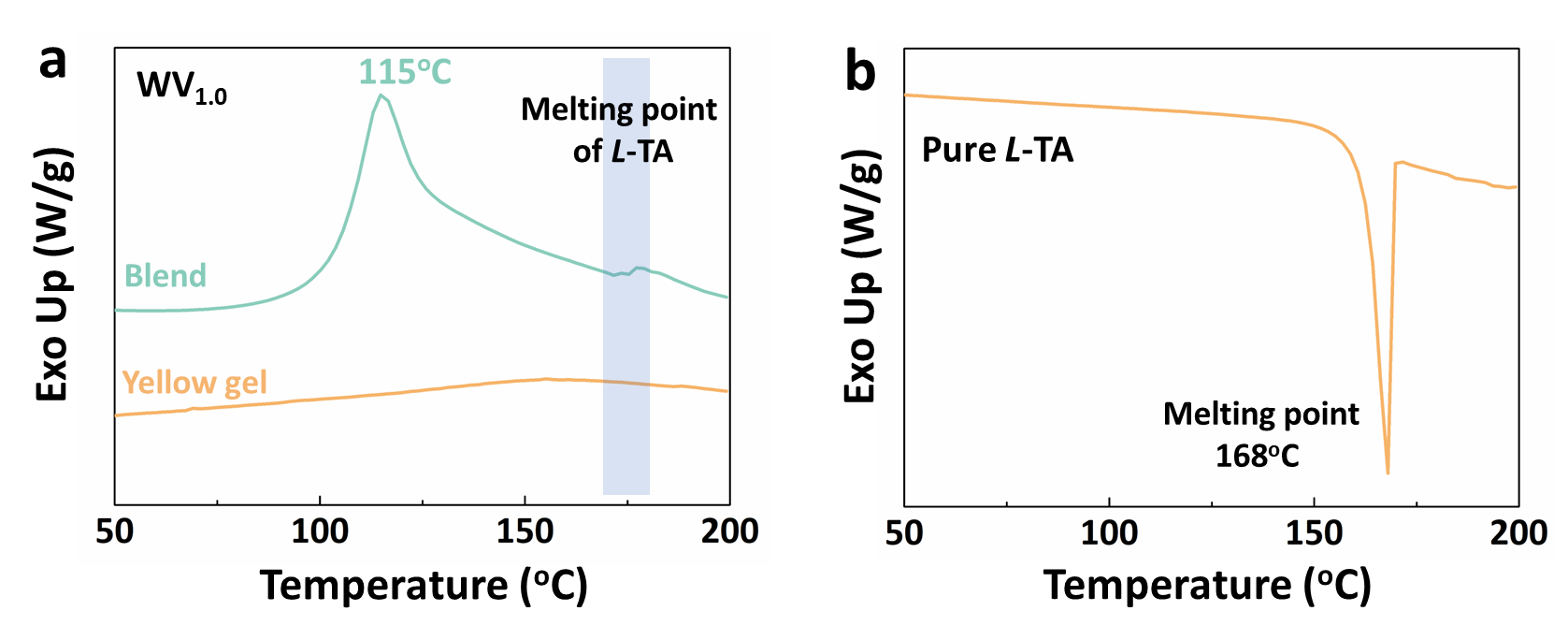


**Figure S6.** DSC curves of BDE/*L*-TA blend, yellow gel of WV_1.0_, and pure *L*-TA at a heating speed of 5℃ min^-1^. (a) DSC curves of BDE/*L*-TA blend prepared without water-assistance (green) and yellow gel of WV_1.0_ (yellow). The stoichiometric ratio of epoxy/carboxyl groups was 1.0. The peak at 115℃ refered to the cross-linking reaction, while the minor fluctuation near 170℃ was attributed to uncured monomer *L*-TA, consistent with the melting point (168℃) of *L*-TA as seen in (b).


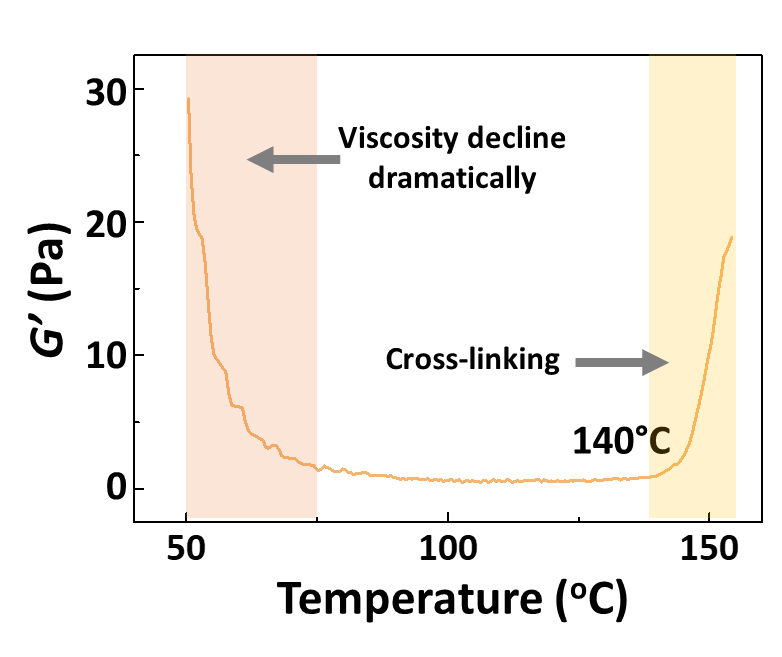


**Figure S7.** Storage modulus (*G*′) of yellow gel of WV_1.0_ from 50 to 170℃ by using rheometer at a heating rate of 5℃ min^-1^. The storage modulus of WV_1.0_ exhibited a significant decrease between 50 to 75℃, followed by a sharp increase beyond 140℃ which attributed to the formation of cross-linked networks.


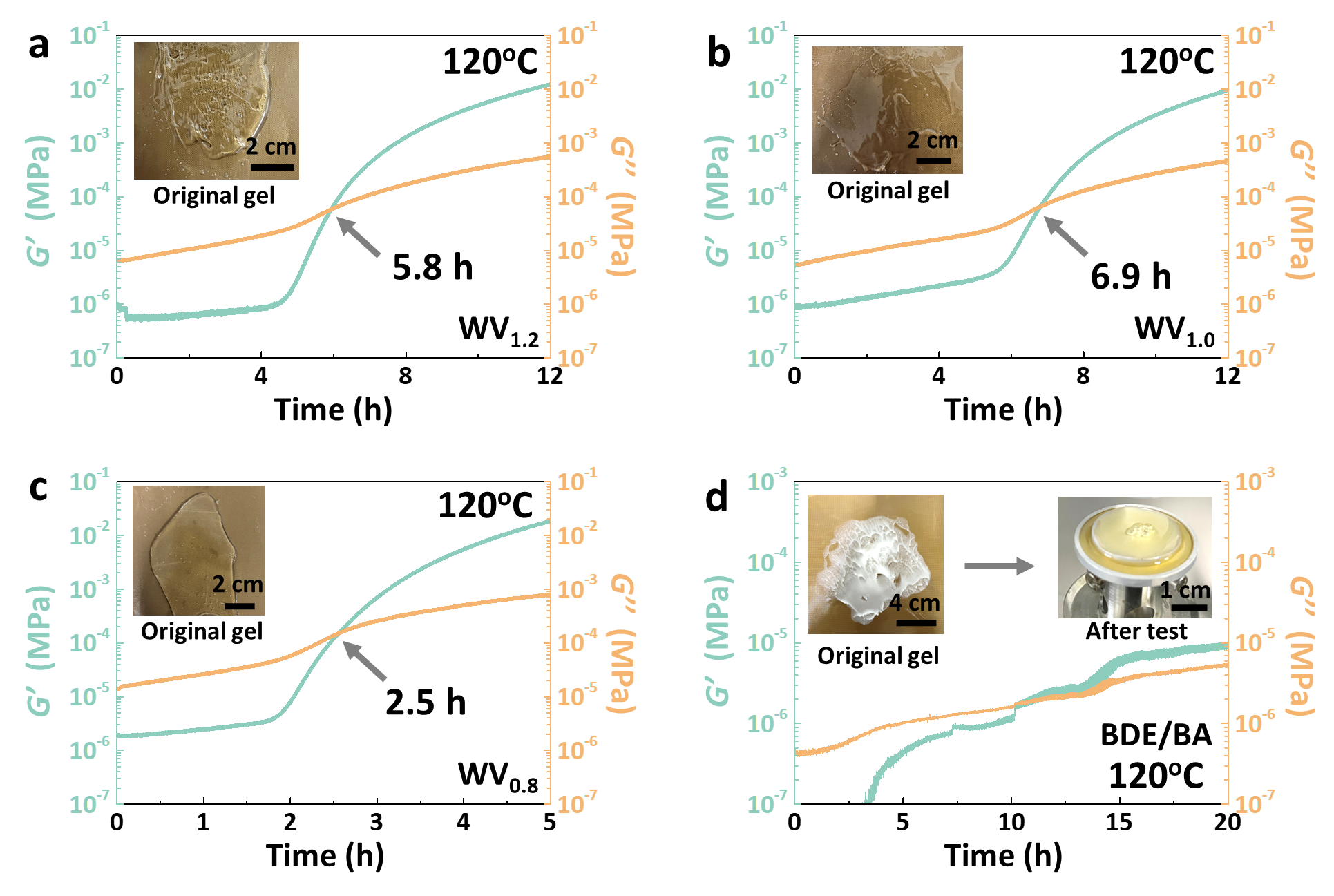


**Figure S8.** Storage modulus (*G*′, green) and loss modulus (*G*′′, orange) of yellow gels of WV_1.2_ (a), WV_1.0_ (b), WV_0.8_ (c), and BDE/BA gel (d) at 120^o^C by using rheometer. All the sample was stirred to precure at 120^o^C by using water-assistance process for 7 h before tests, obtaining gel as seen in inset photographs. The gel point for WV_1.2_, WV_1.0_, WV_0.8_ was 5.8, 6.9, and 2.5 h, respectively. The BDE/BA gel showed no gelation before 10 h, maintaining a flowable uncured state with minimal *G*′ throughout 20-hour testing.


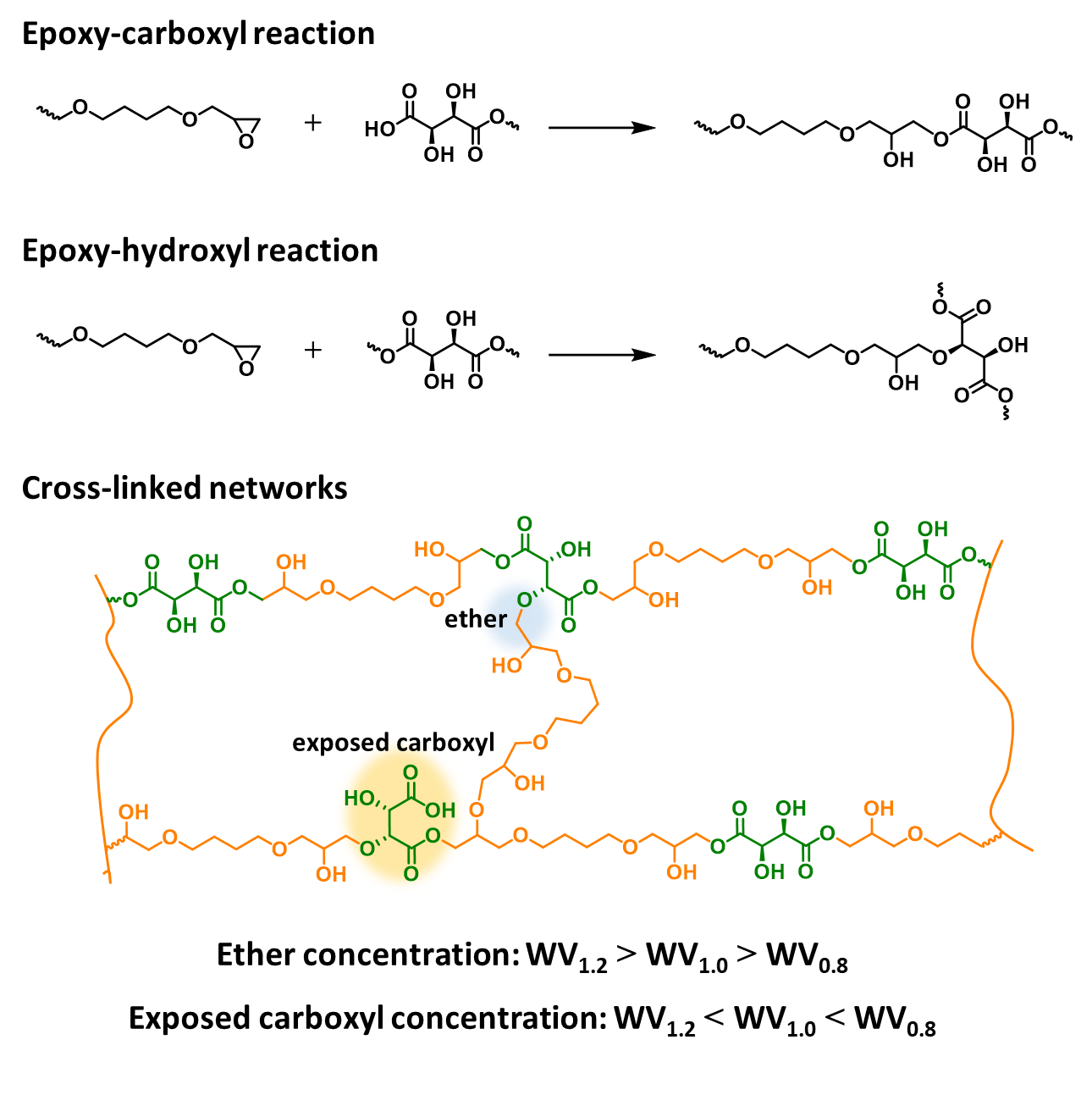


**Figure S9.** Mechanism of cross-linked networks formation. The epoxy groups undergo a reaction with the carboxyl groups (-COOH) provided by *L*-TA, forming ester bonds while simultaneously generating hydroxyl groups (-OH). Meanwhile, these newly formed hydroxyl groups (or those originally present in *L*-TA) also react with remaining epoxy groups, leading to the formation of ether linkages. As a result, cross-linked networks were formed through a combination of ester and ether bonds, with a fraction of carboxyl groups remaining unreacted.


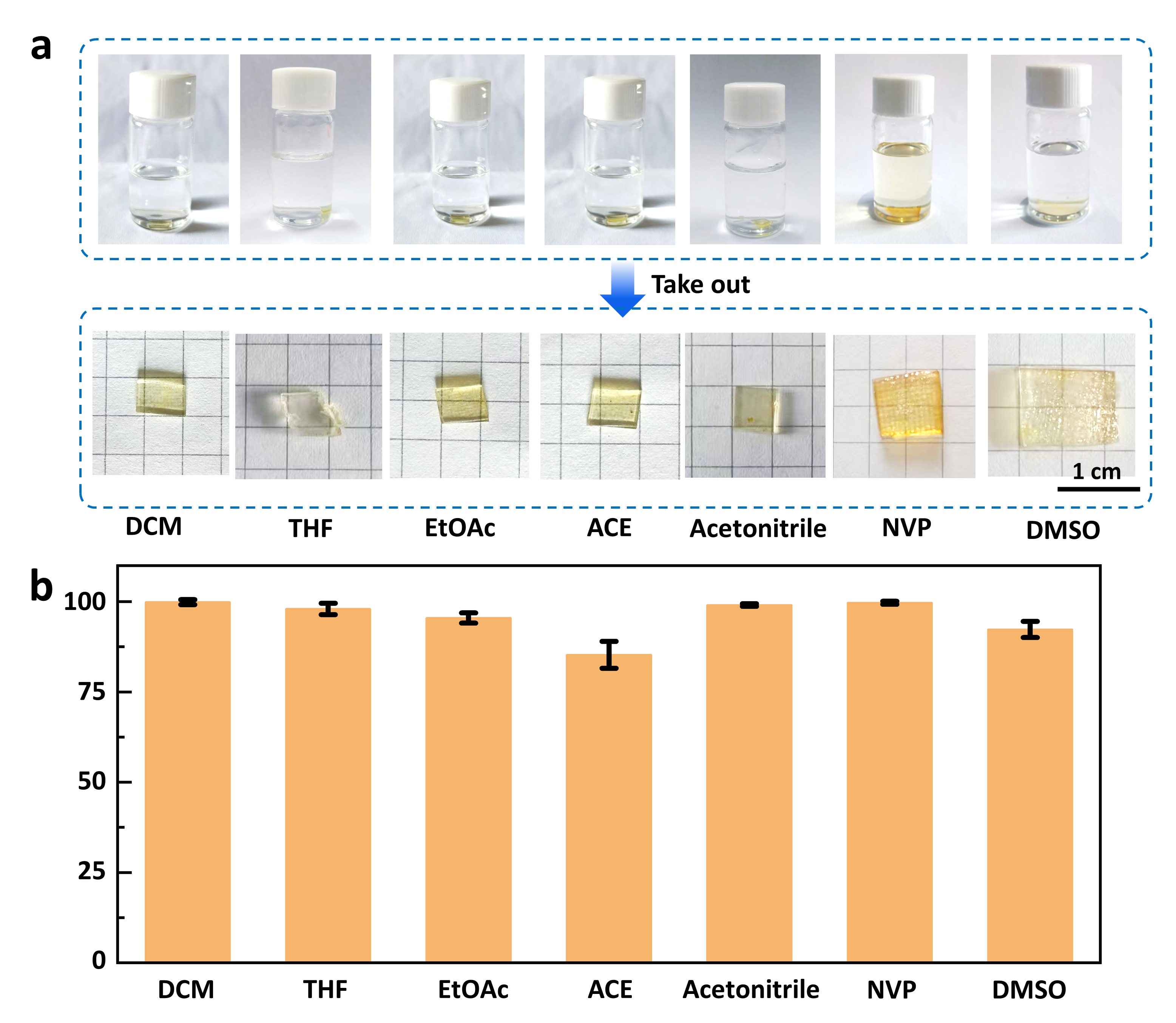


**Figure S10.** Gel content test of WV_1.0_ in different aprotic solvents. (a) Photographs of the WV_1.0_ sample (area 5 mm × 5 mm) in dichloromethane (DCM), tetrahydrofuran (THF), ethyl acetate (EtOAc), acetone, acetonitrile, N-vinyl-2-pyrrolidone (NVP) and dimethyl sulfoxide (DMSO) after gel content test. The sample was taken out and set on the quadrille paper. (b) Gel content values of WV_1.0_ in different solvents for 25 h at room temperature. Error bars represent the SD derived from triplicate measurements.


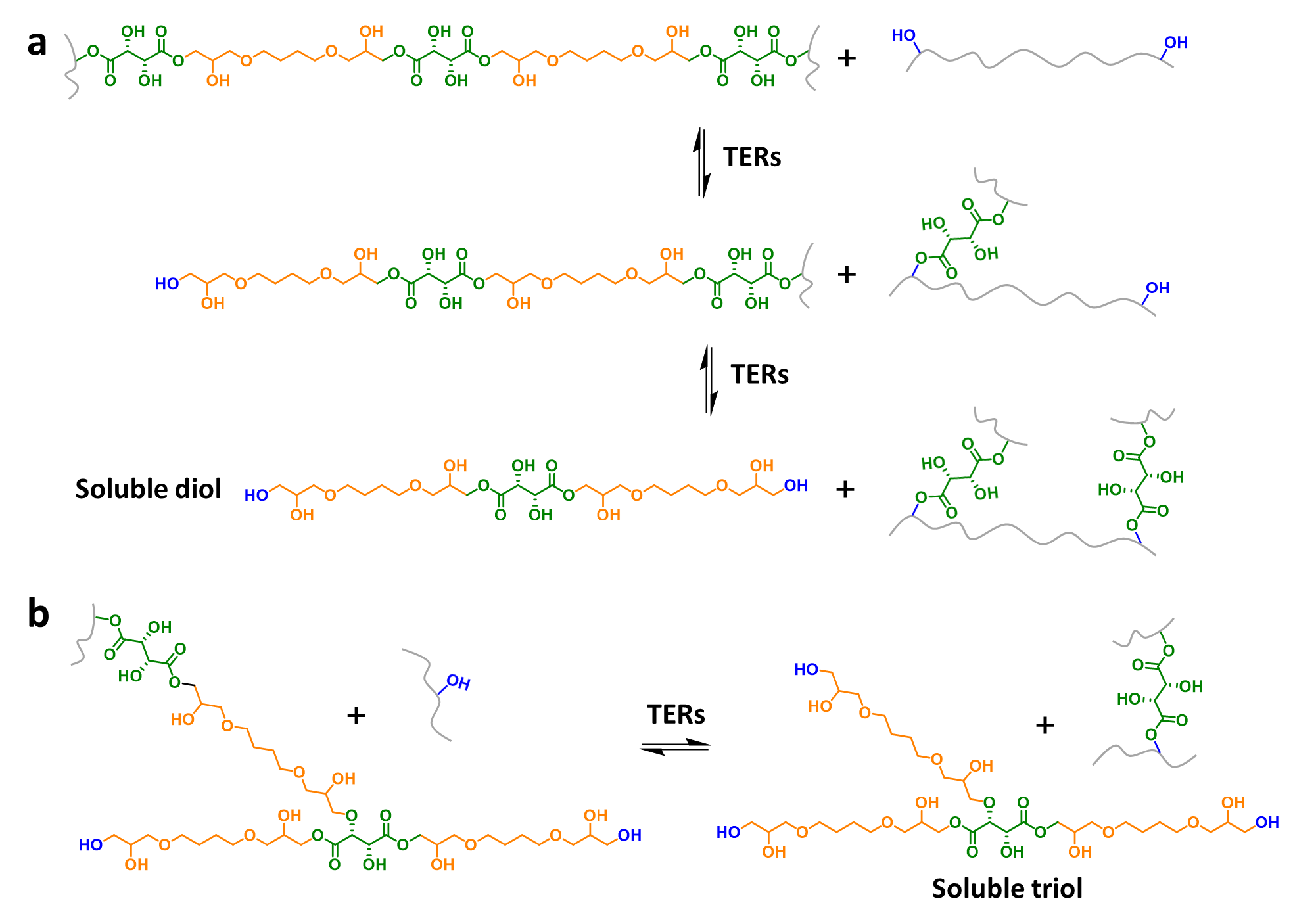


**Figure S11.** Proposed mechanism for the generation of soluble oligomers through transesterification reactions (TERs) under high-temperature or swelling conditions. Diol (a) and triol (b) oligomers could generate from cross-linked networks.


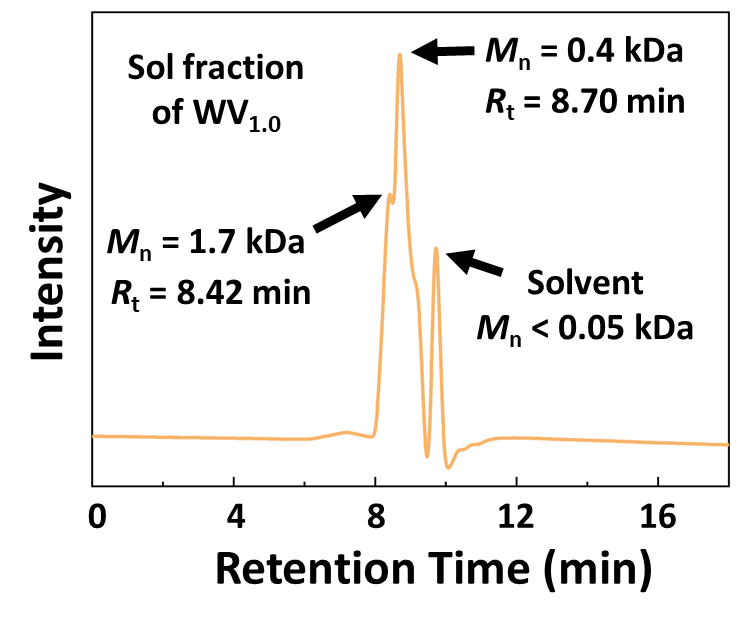


**Figure S12.** Gel permeation chromatography (GPC) of the sol fraction extracted from WV_1.0_ after immersion in acetone. The number-average molecular weight (*M*ₙ) of the sol fraction corresponded to two major oligomeric species: 0.4 kDa (retention time *R*ₜ = 8.70 min) and 1.7 kDa (*R*ₜ = 8.42 min). The peak at *R*ₜ = 9.72 min was attributed to the solvent (*M*ₙ < 0.05 kDa). These results confirmed that the sol fraction consisted primarily of oligomers rather than unreacted monomers.


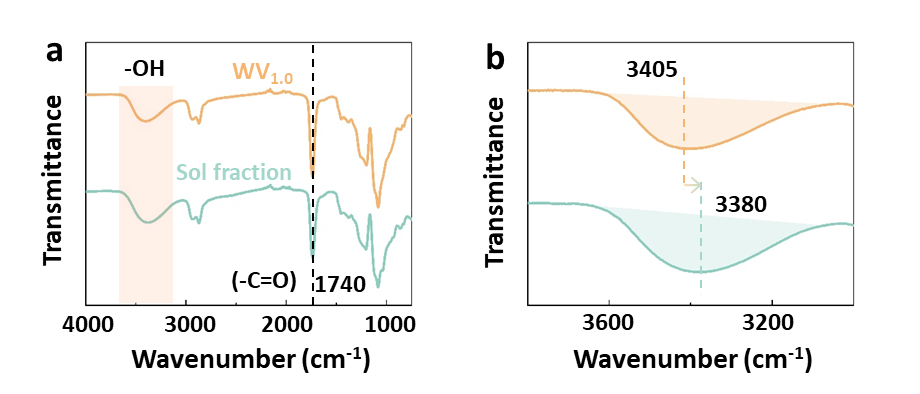


**Figure S13.** FTIR spectra of WV_1.0_ network and the sol fraction of WV_1.0_. The wavenumbers from 4000 to 500 cm^-1^ (a) and 3800 to 3000 cm^-1^ (b). The sol fraction of WV_1.0_ exhibited a chemical structure largely similar to that of the original WV_1.0_ networks, confirming that the sol fraction consisted of oligomers connected by BDE and *L*-TA. Meanwhile, the hydroxyl peak at 3405 cm^-1^ shifted to 3380 cm^-1^, which could be attributed to the easier formation of hydrogen bonds between oligomers compared to those within macromolecular chains.


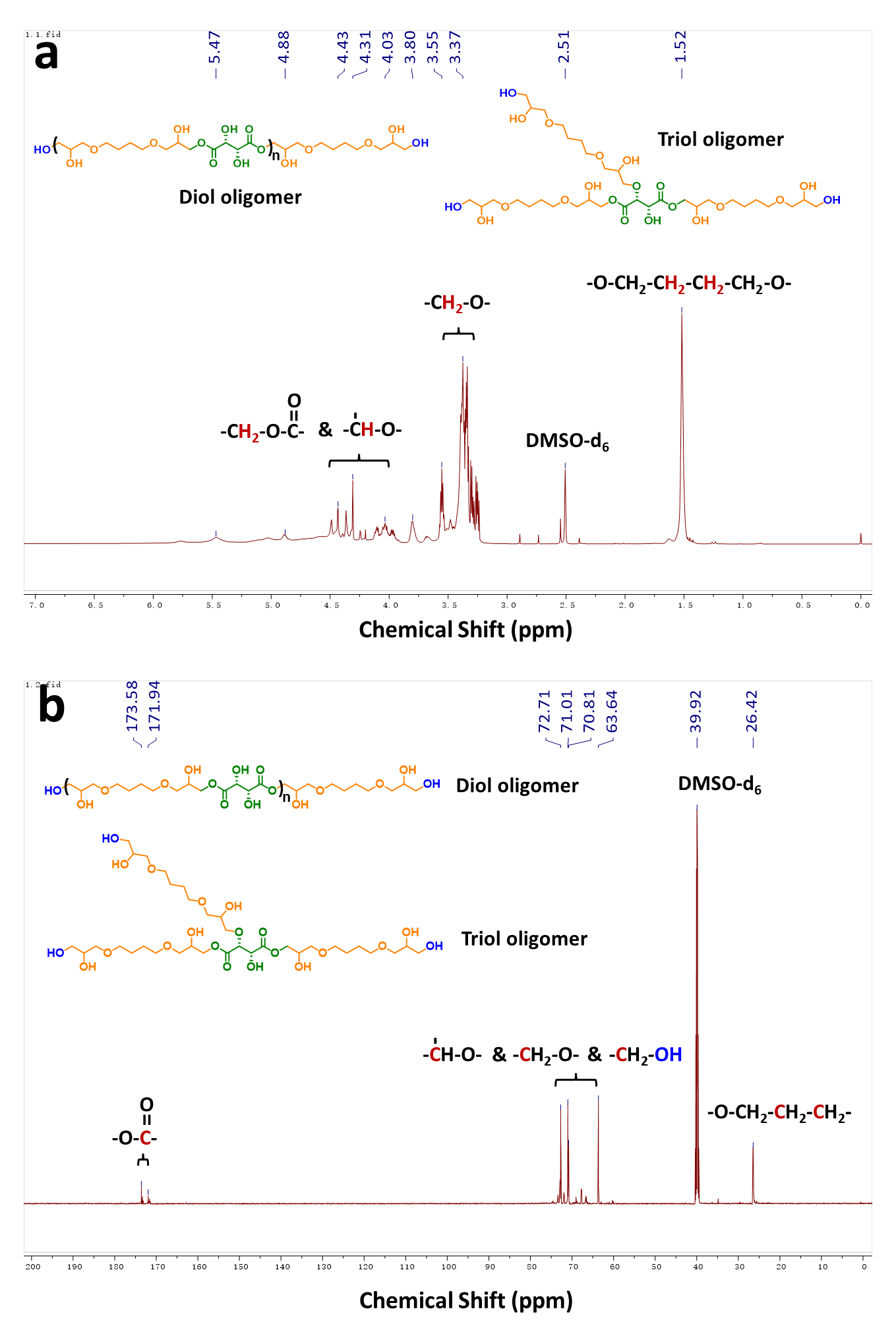


**Figure S14**. ^1^H NMR (a) and ^13^C NMR (b) spectra of the sol fraction of WV_1.0_ in DMSO-d_6_. The presence of the proposed diol and triol oligomers could be deduced from transesterification reactions (TERs) as illustrated in Figure S11. The signals corroborated the oligomeric structures in ^1^H NMR: the multiplet at 1.52 ppm was assigned to methylene protons adjacent to other methylene units (-O-CH_2_-C*H_2_*-C*H_2_*-CH_2_-O-), while the resonances between 3.37-3.55 ppm were attributed to methylene groups linked to both ether and hydroxyl functionalities (-C*H_2_*-O-). The region between 4.08-4.33 ppm included signals from methylene protons adjacent to ester groups, as well as methine protons. Corresponding carbon signals are observed in the ^13^C NMR spectrum: the peak at 26.42 ppm was assigned to aliphatic methylene carbon, and the cluster at 63.64-72.71 ppm confirmed the presence of carbons bonded to oxygen in termini primary hydroxyl, ether and other hydroxyl groups. Meanwhile, carbonyl carbon signals appeared at 171.94 ppm and 173.85 ppm in ^13^C NMR spectrum. The presence of two separate groups of peaks suggested different chemical environments for these carbonyl groups originating from *L*-TA, indicating that partial ether bond formation occurred at the hydroxyl groups attached to the adjacent chiral carbon centers. These results demonstrated that diol and triol oligomers generated by TERs were present in the soluble fraction.


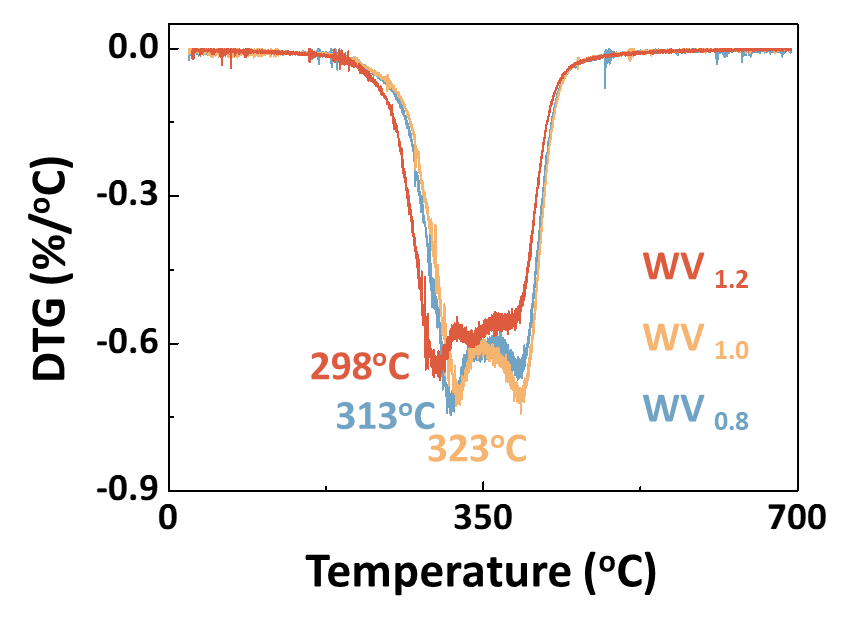


**Figure S15.** DTG curves of WV_1.2_, WV_1.0_, and WV_0.8_. The mass loss peaks of WV_1.2_, WV_1.0_ and WV_0.8_ was 298, 323 and 313℃, respectively.


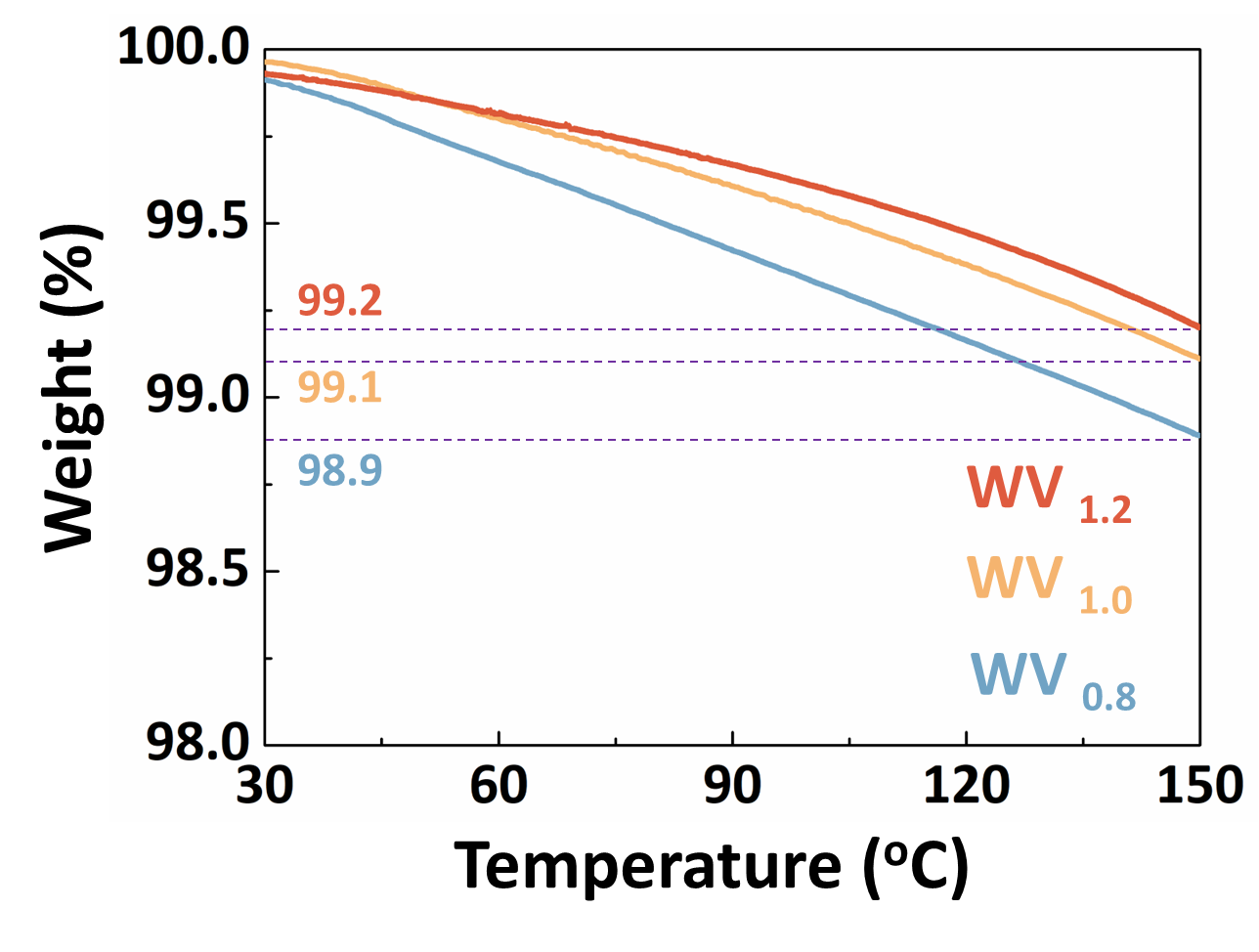


**Figure S16.** TGA curves of waterborne vitrimers ranging from 30 to 150^o^C from Figure 2e. The weight loss was 0.8%, 0.9% and 1.1% for WV_1.2_, WV_1.0_, and WV_0.8_, respectively.


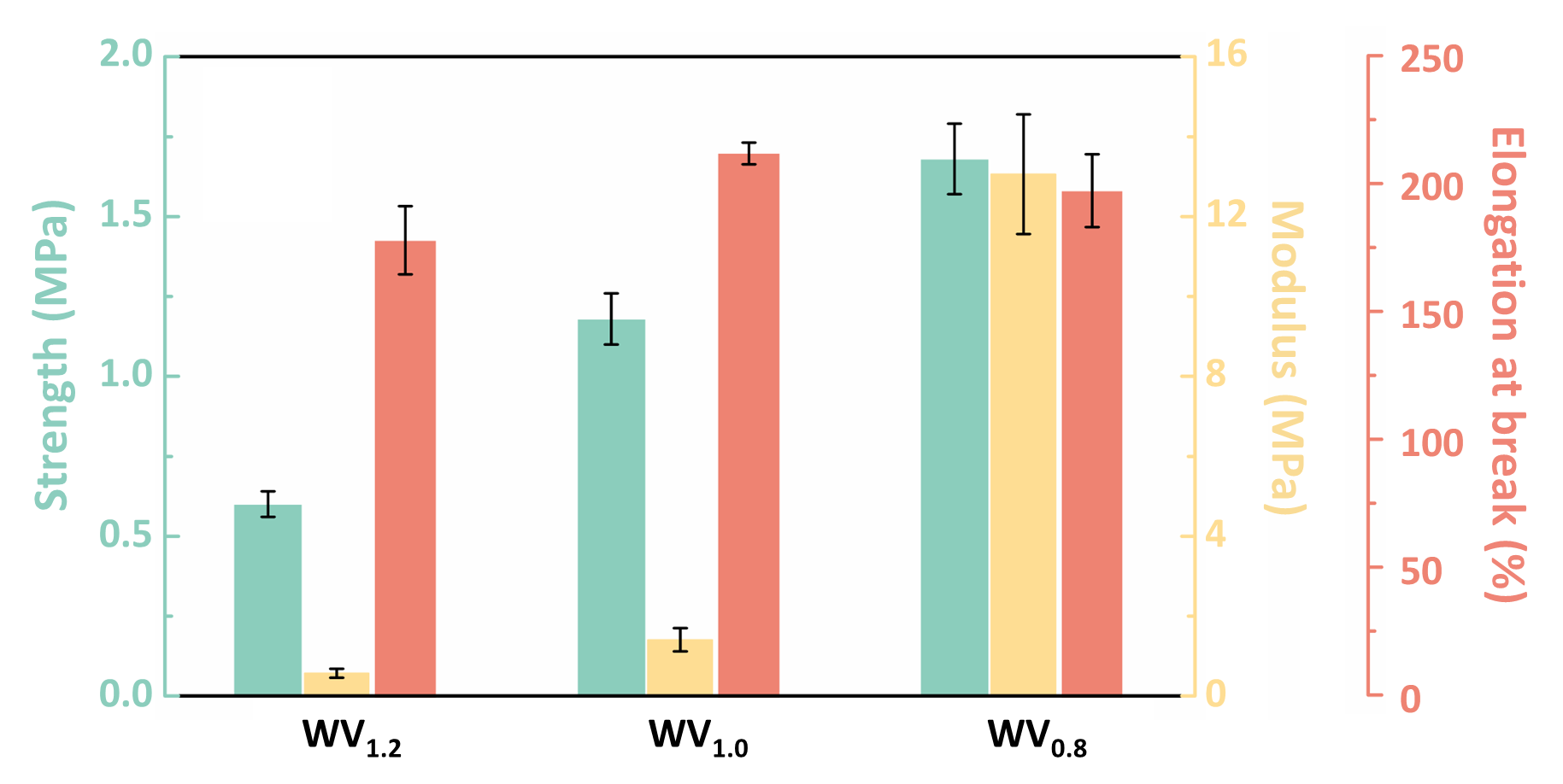


**Figure S17.** Tensile strength (green), Young’s modulus (yellow), and elongation at break (red) of WV_1.2_, WV_1.0_, and WV_0.8_. Error bars represent the SD derived from quintuplicate measurements.


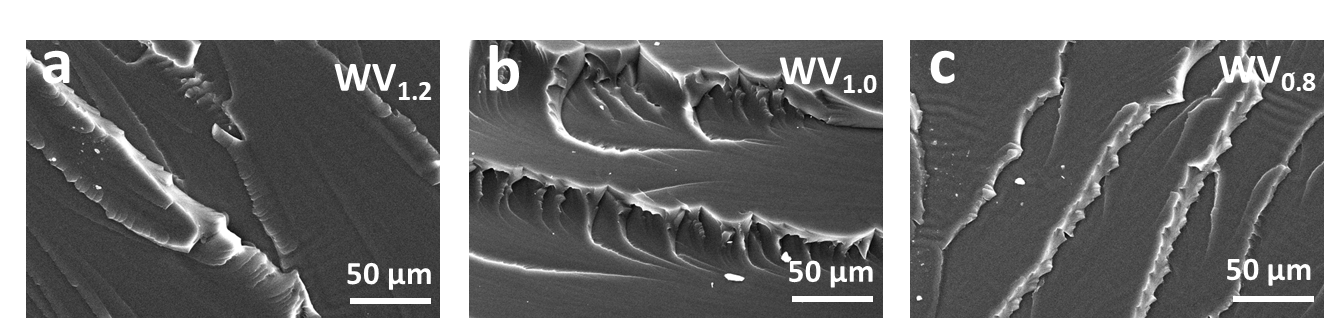


**Figure S18**. Scanning electron microscope (SEM) images of WV_1.2_ (a), WV_1.0_ (b) and WV_0.8_ (c). The SEM images were the fracture surface of rectangular samples, exhibiting characteristic ductile fracture morphology. The microscopic analyses also indicated the absence of microstructural defects in all systems.


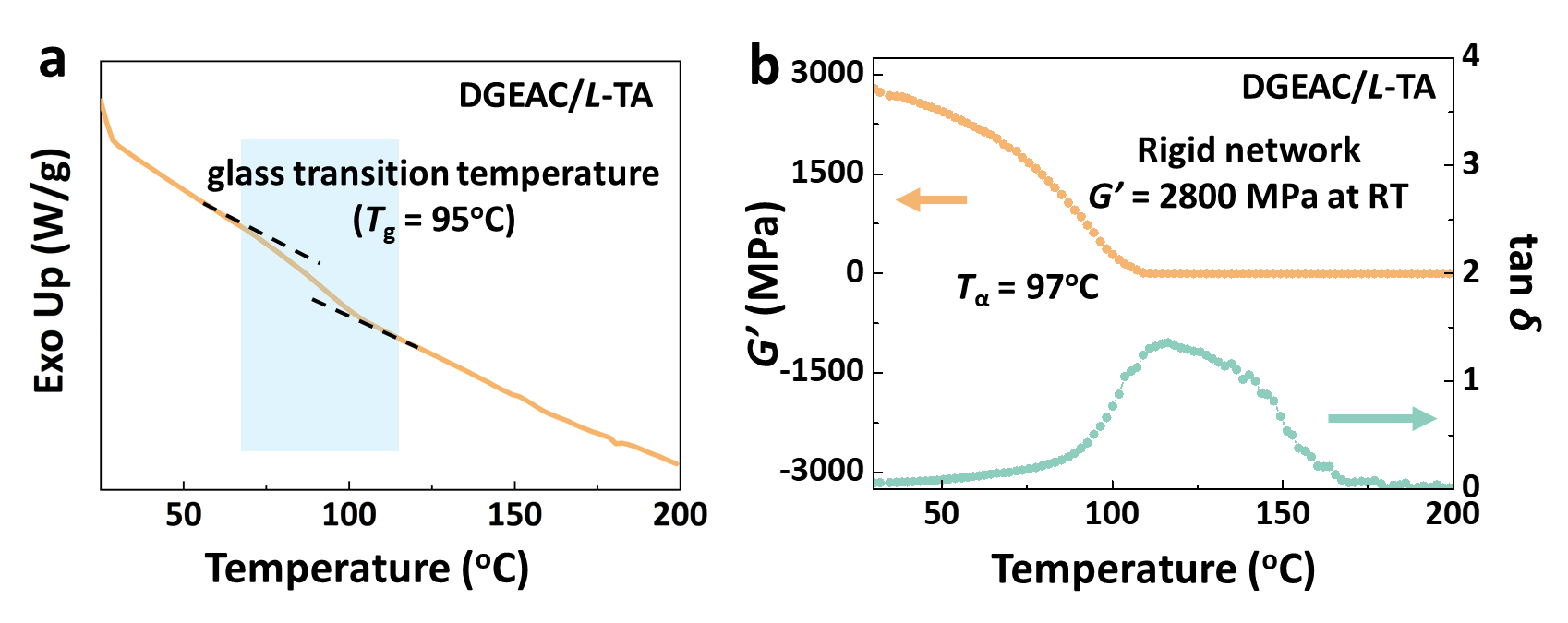


**Figure S19**. DSC curve (a) and dynamic mechanical properties (b) of DGEAC/*L*-TA. Heating speed of DSC and DMA was 10^o^C min^-1^ and 5^o^C min^-1^, respectively. The glass transition temperature (*T*_g_ = 95^o^C and *T*_α_ = 97^o^C) of DGEAC/*L*-TA was concluded from the point corresponding to the maximum negative slope (inflection point) in the decreasing segment of the DSC and storage modulus curve, respectively. The *G*′ was 2800 MPa at room temperature.


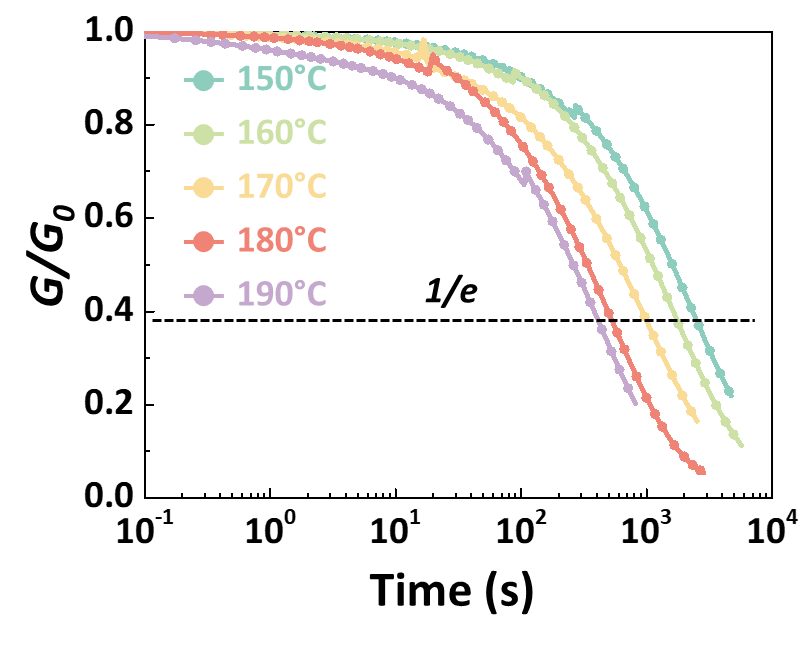


**Figure S20.** Stress relaxation of WV_1.2_ at 150, 160, 170, 180 and 190℃ by using rheometer.


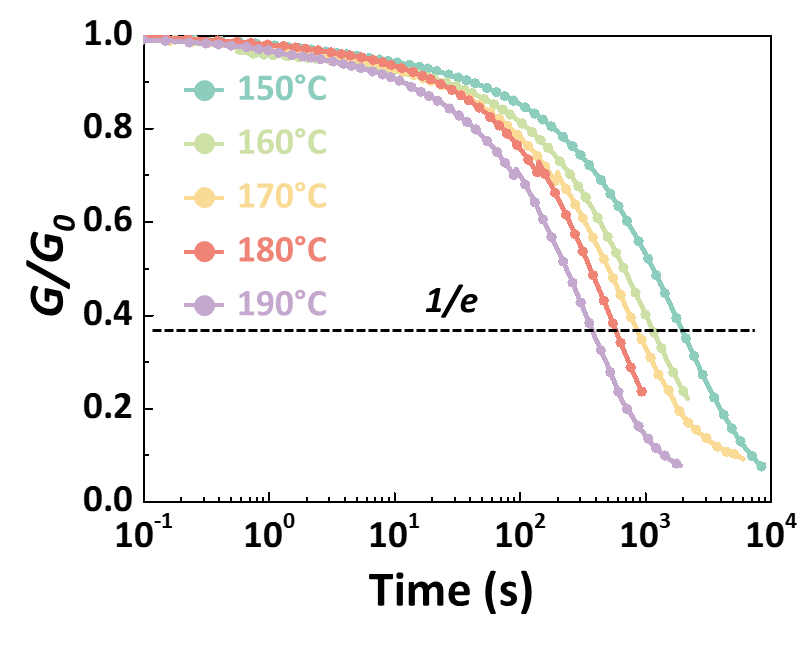


**Figure S21.** Stress relaxation of WV_0.8_ at 150, 160, 170, 180 and 190℃ by using rheometer.


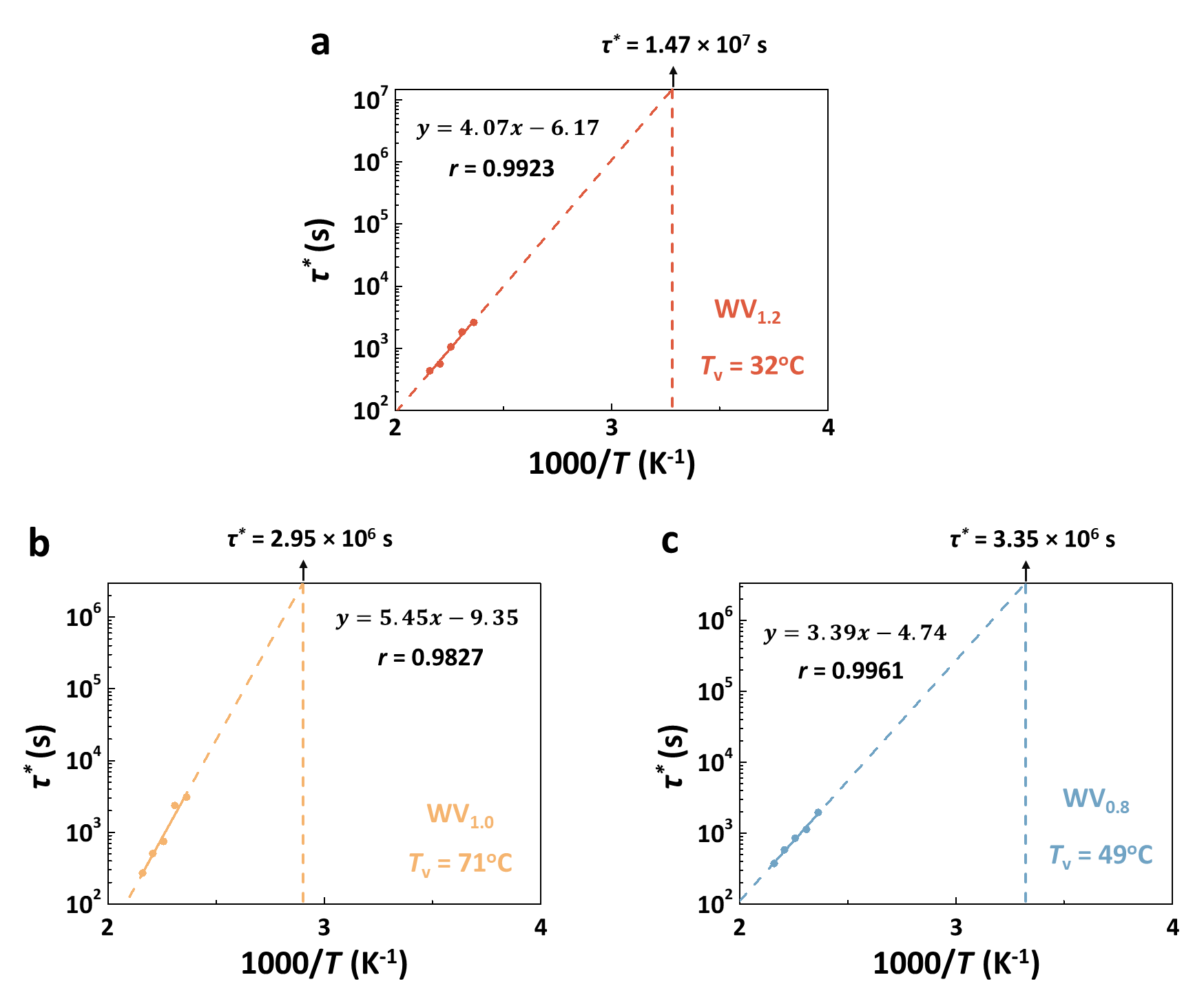


**Figure S22.** Determination of the topology freezing transition temperature (*T*_v_) of WV_1.2_ (a), WV_1.0_ (b) and WV_0.8_ (c). The fitting lines, adhering to the Arrhenius law, were extrapolated to ascertain the value of *τ** at a viscosity of *ƞ* = 10^12^ Pa s^-1^.


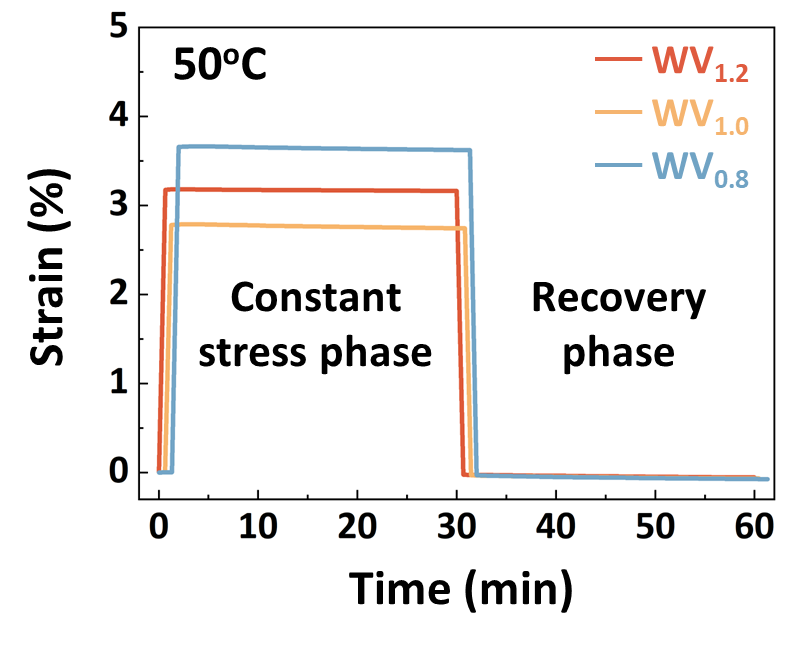


**Figure S23.** The creep-recovery of WV_1.2_, WV_1.0_, and WV_0.8_ at 50^o^C. Constant stress of 0.2 MPa was applied before 30 min, followed by stress removal from 30 to 60 min. To make the curve clearer under the instantaneous stress, the samples were maintained at no stress condition for the first few seconds. All samples exhibited purely elastic deformation with complete recovery (~100%), demonstrating negligible creep due to their cross-linked network structures.


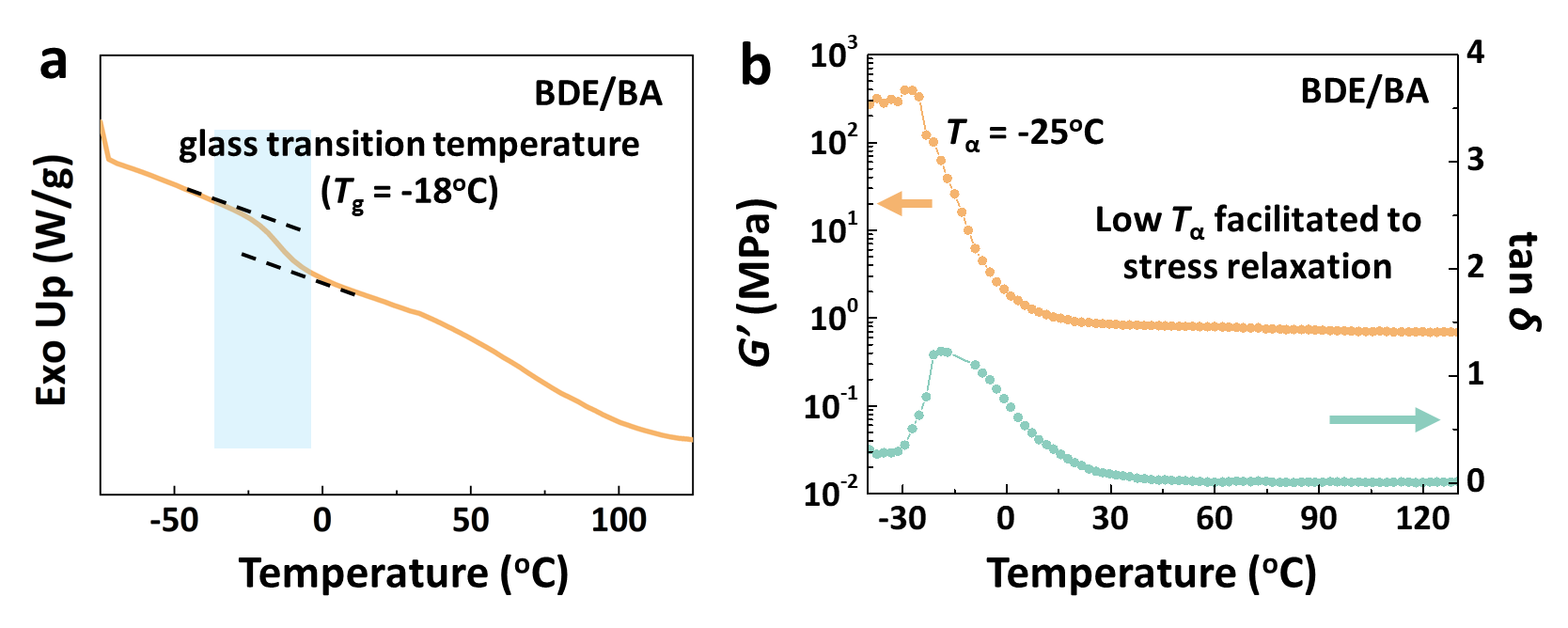


**Figure S24.** DSC curve (a) and dynamic mechanical properties (b) of BDE/BA. Heating speed of DSC and DMA was 10^o^C min^-1^ and 5^o^C min^-1^, respectively. The glass transition temperature (*T*_g_ = -18^o^C and *T*_α_ = -25^o^C) of DGEAC/*L*-TA was concluded from the point corresponding to the maximum negative slope (inflection point) in the decreasing segment of the DSC and storage modulus curve, respectively.


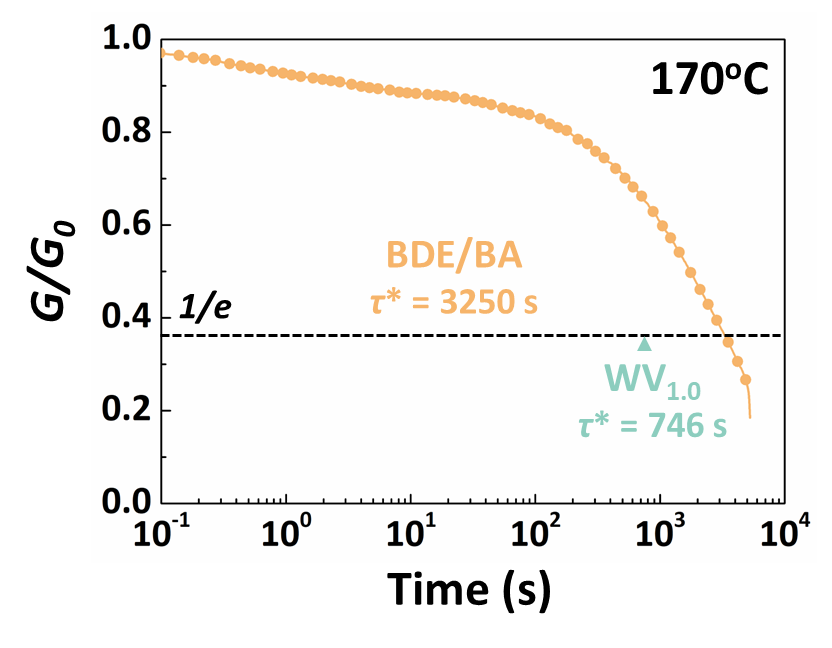


**Figure S25.** Stress relaxation of BDE/BA at 170^o^C by using rheometer. The relaxation time (*τ**) of BDE/BA was 3250 s, much longer than that of WV_1.0_ (746 s, also see Figure 3a).


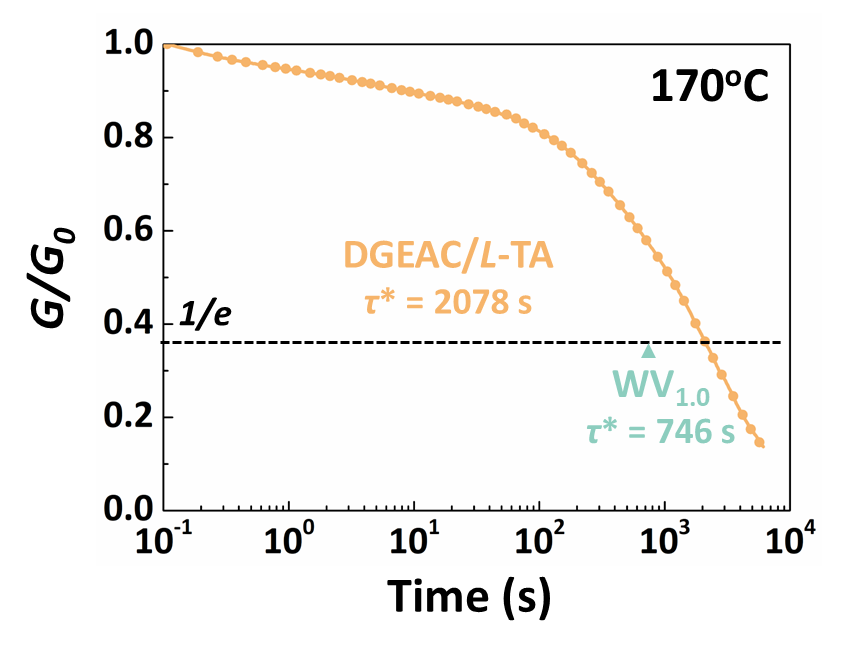


**Figure S26**. Stress relaxation of DGEAC/*L*-TA at 170^o^C by using rheometer. The relaxation time (*τ**) of BDE/BA was 2078 s, much longer than that of WV_1.0_ (746 s, also see Figure 3a).


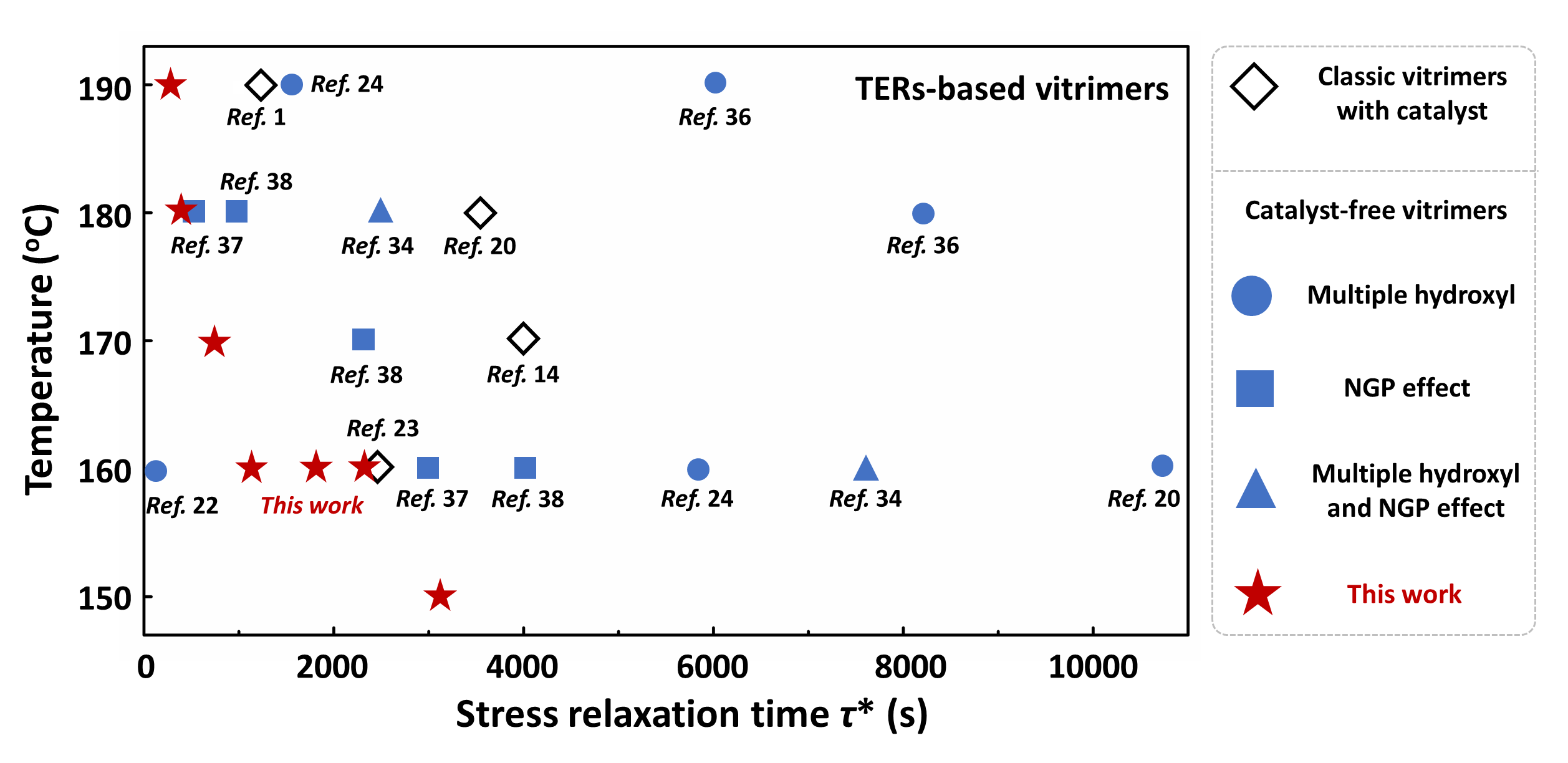


**Figure S27.** The relaxation time of waterborne vitrimers and other TERs-based vitrimers. The hollow diamond symbol denotes classic vitrimers with catalysts. The circle, square, and triangle symbols represent catalyst-free vitrimers, which are modulated by multiple hydroxyl groups, NGP and the combined effect of muitiple hydroxyl groups and NGP, respectively. The star symbol signifies vitrimers developed in this study. All reference numbers are consistent with those in main text.


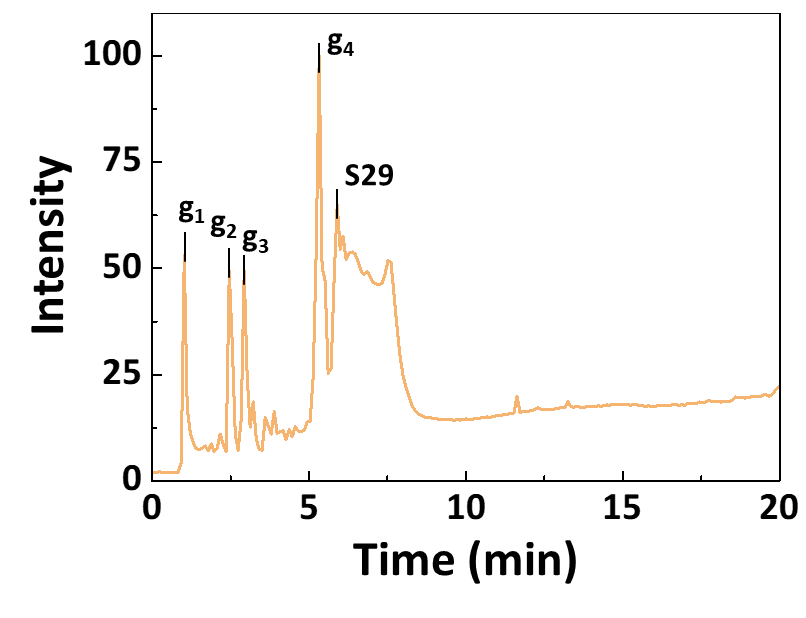


**Figure S28.** HPLC of the degradation products include g_1_, g_2_, g_3_, g_4_, and S29 at the retention time of 1.02, 2.53, 2.93, 5.32 and 5.90 min. The corresponding mass analysis of g_1_, g_2_, g_3_, g_4_, and S29 was shown in Figure 3g, Figure S29 and Table S5.


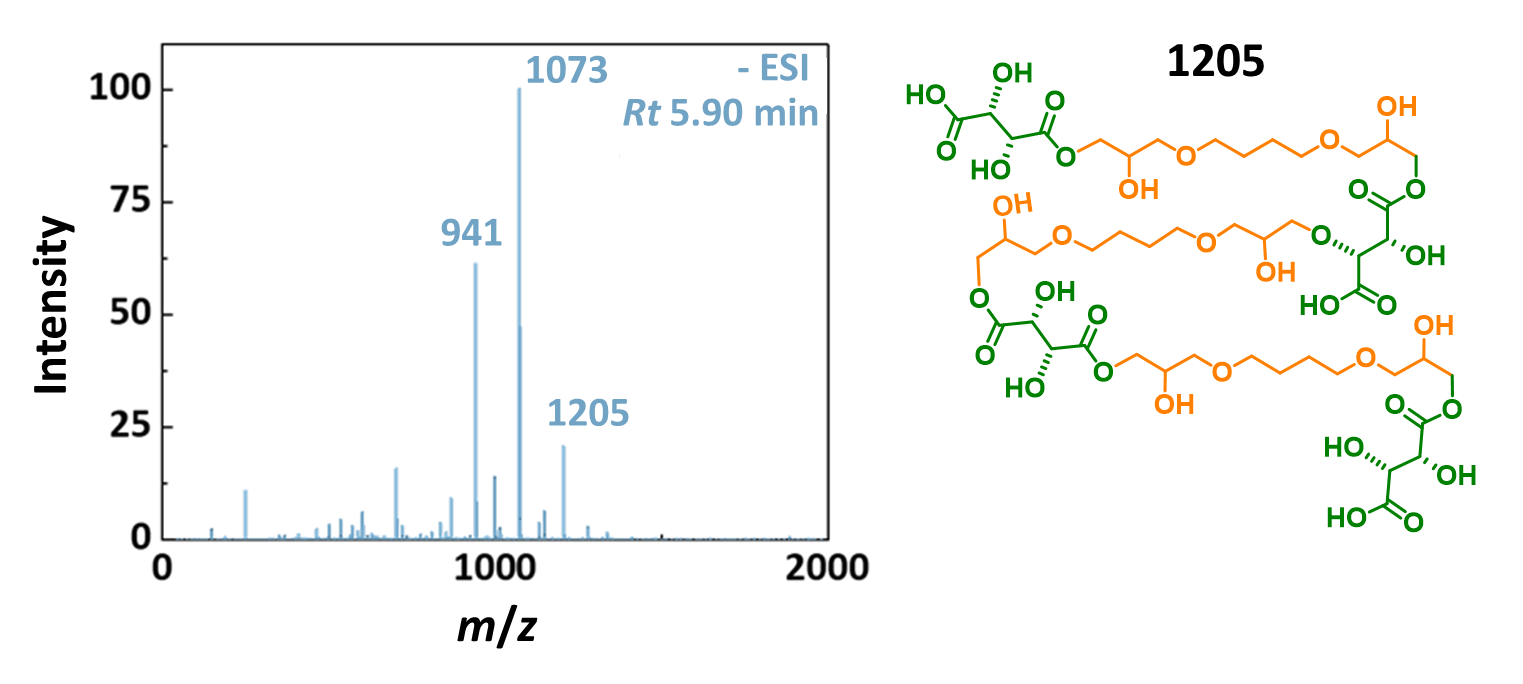


**Figure S29.** Peak S29 showed [M - H]^-^ at *m*/*z* 1205 (left), and the proposed chemical structures (right). The proposed chemical structures of potential corresponding fragments in the mass spectrometry (MS) spectrum was shown in Table S5, including [M - H]^-^ at *m*/*z* 941 and 1073.


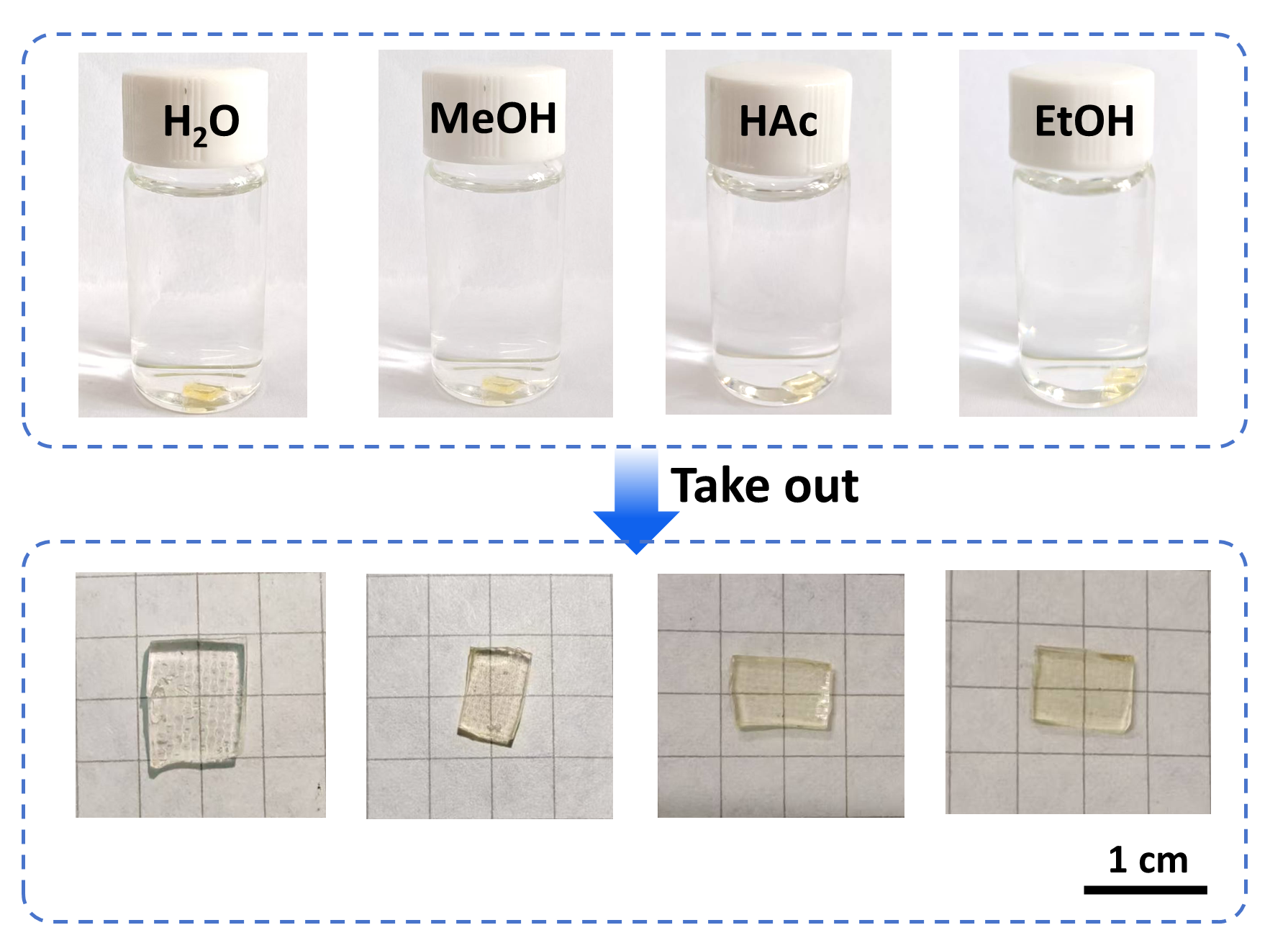


**Figure S30.** Gel content tests of WV_1.0_ in water and other protic solvents. Photographs illustrated the WV_1.0_ sample (area 5 mm × 5 mm) in water, methanol (MeOH), acetic acid (HAc), and ethanol (EtOH) after gel content tests.


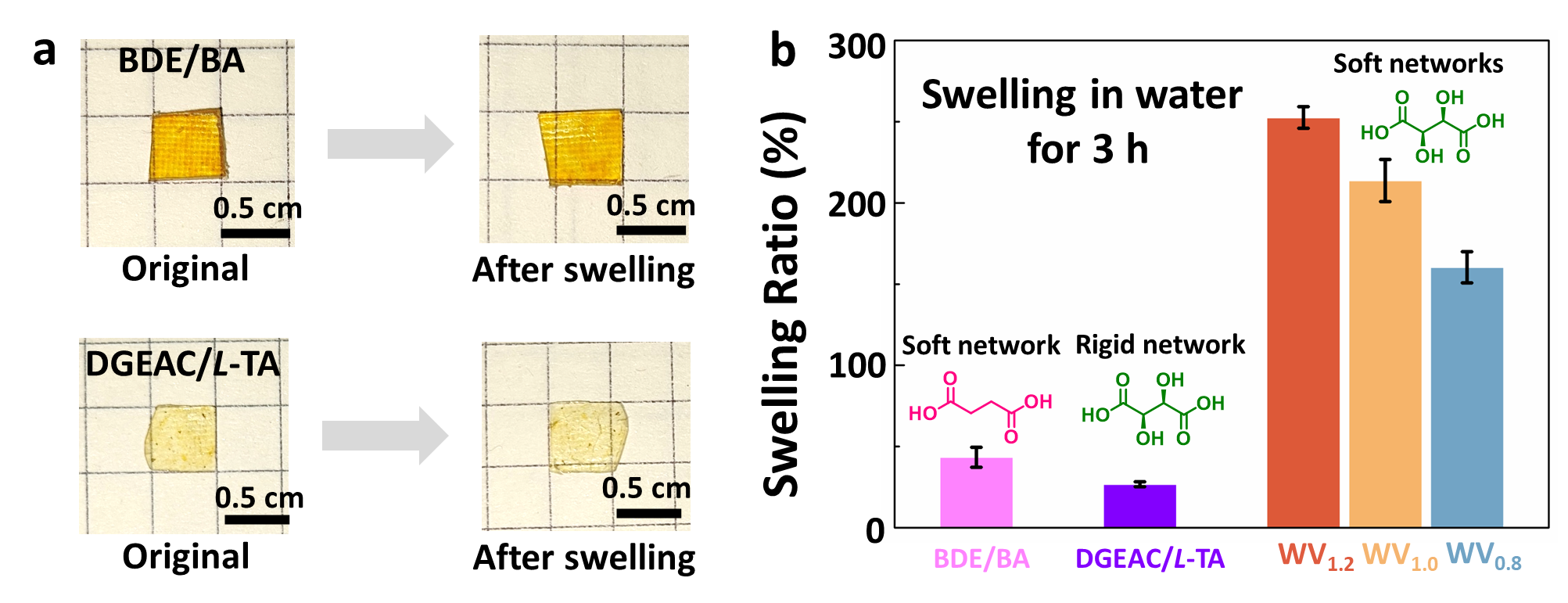


**Figure S31.** Comparison of swelling ratio of DGEAC/*L*-TA, BDE/BA and waterborne vitrimers in water at room temperature for 3 h. (a) Photographs of BDE/BA and DGEAC/*L*-TA materials before and after swelling. The results show that the volumn of samples changed a little after swelling. (b) Swelling ratio of BDE/BA, DGEAC/*L*-TA and waterborne vitrimers after immerging in water for 3 h. Error bars represent the SD derived from triplicate measurements.


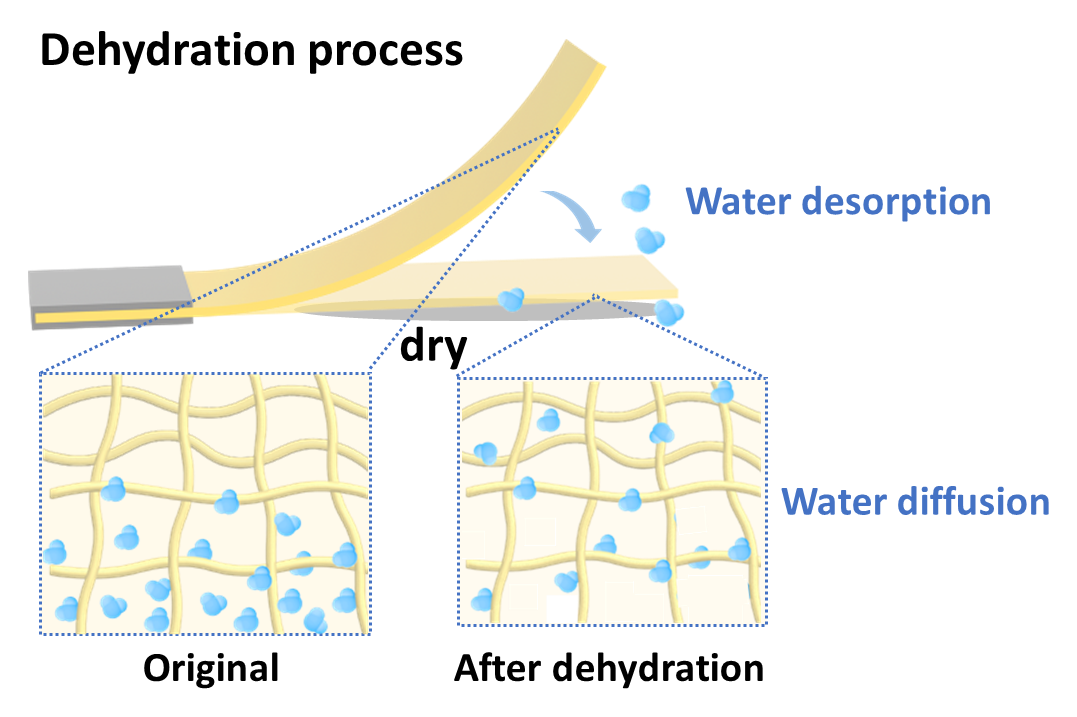


**Figure S32.** Schematic diagram of the reversible water-driven behavior of the WV_1.0_ film upon a dry filter paper.


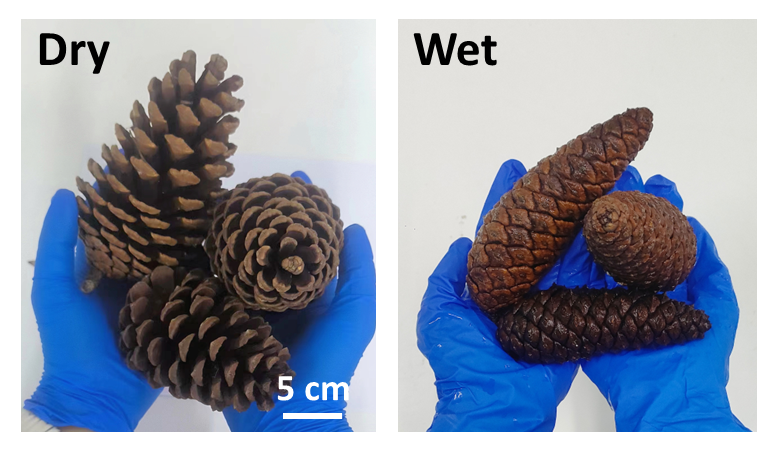


**Figure S33.** Pinecones placed in dry (left) and wet (right) environments. These pinecones closed their scales upon hydration and reopened them upon dehydration. For the hydration process, artificial watering was applied to simulate a rainy environment.


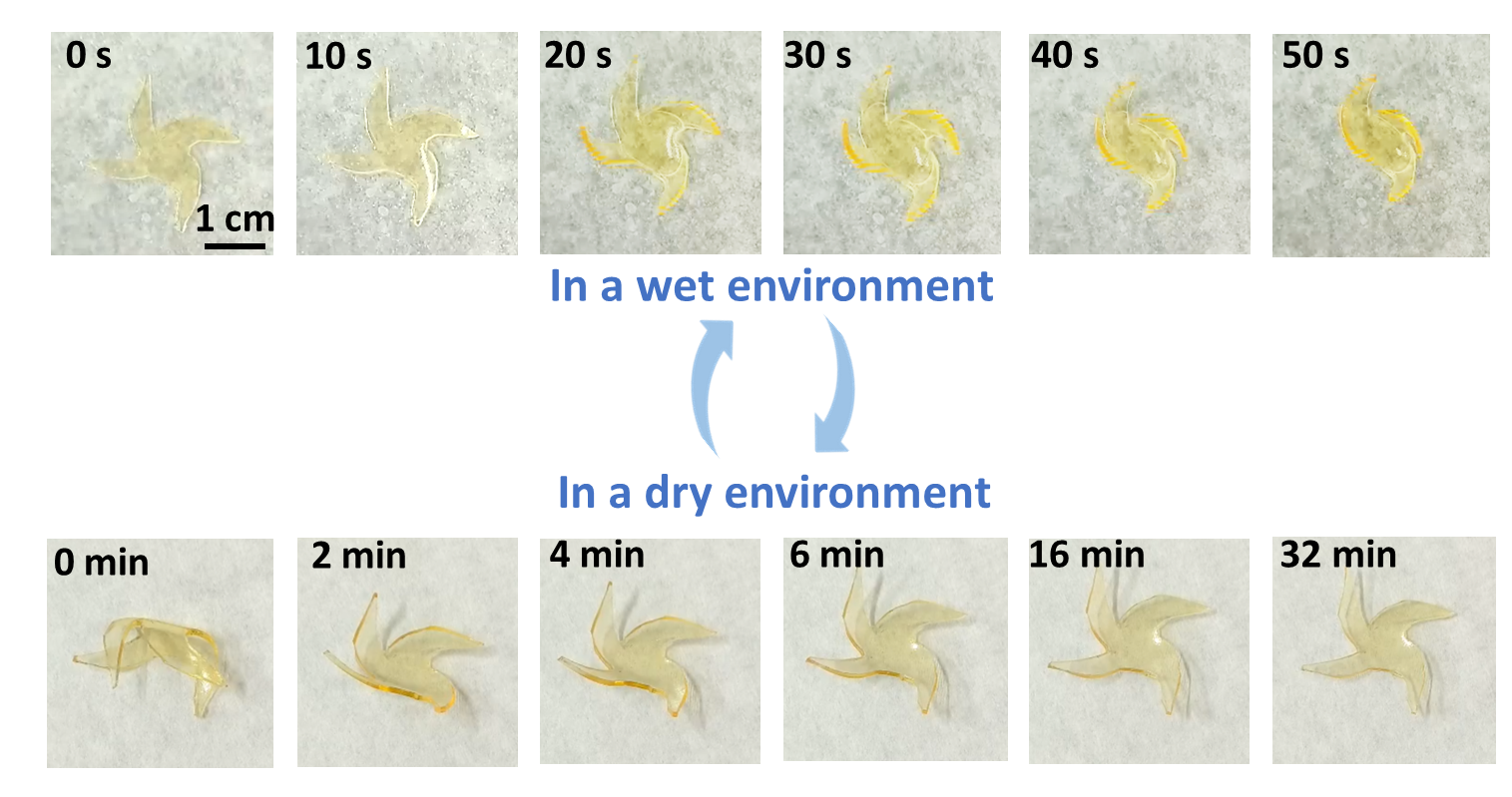


**Figure S34.** Photographs of WV_1.0_ film with a thickness of 1 mm upon a wet and dry filter paper from Movie S1.


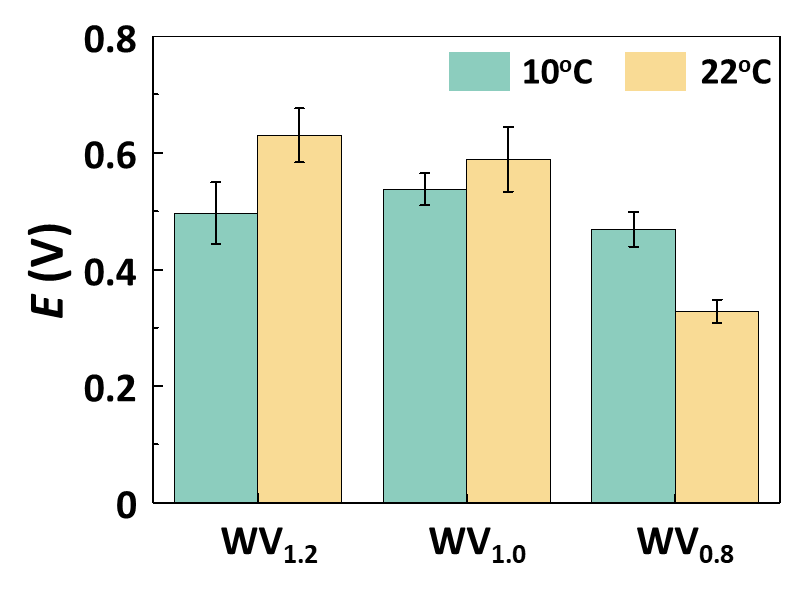


**Figure S35.** Voltage output of WV-MEG at 10℃ and 22℃ (RH = 60%). Error bars represent the SD derived from triplicate measurements.


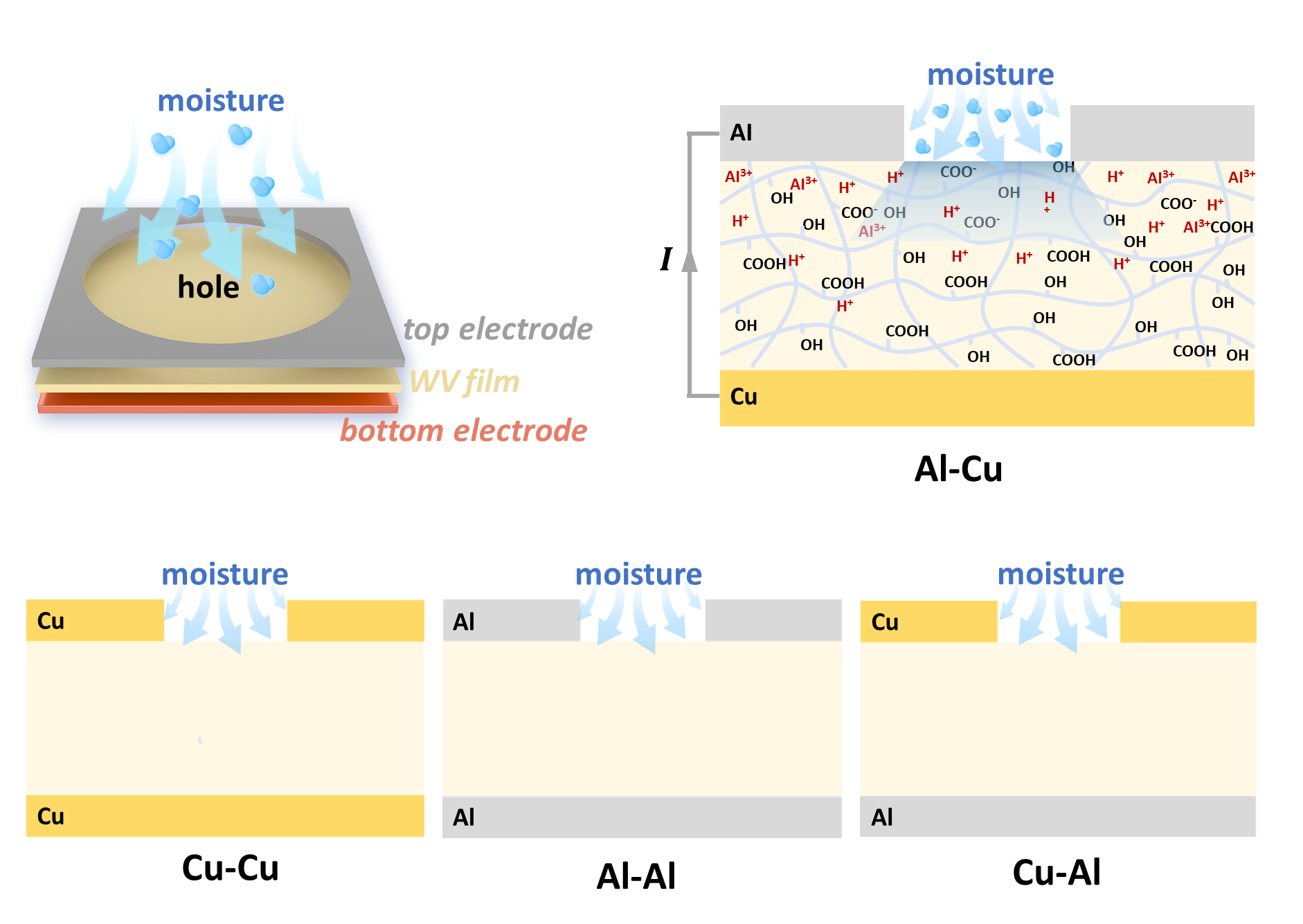


**Figure S36.** Schematic of vitrimer derived moist-electric generator (WV-MEG) by using different electrodes.


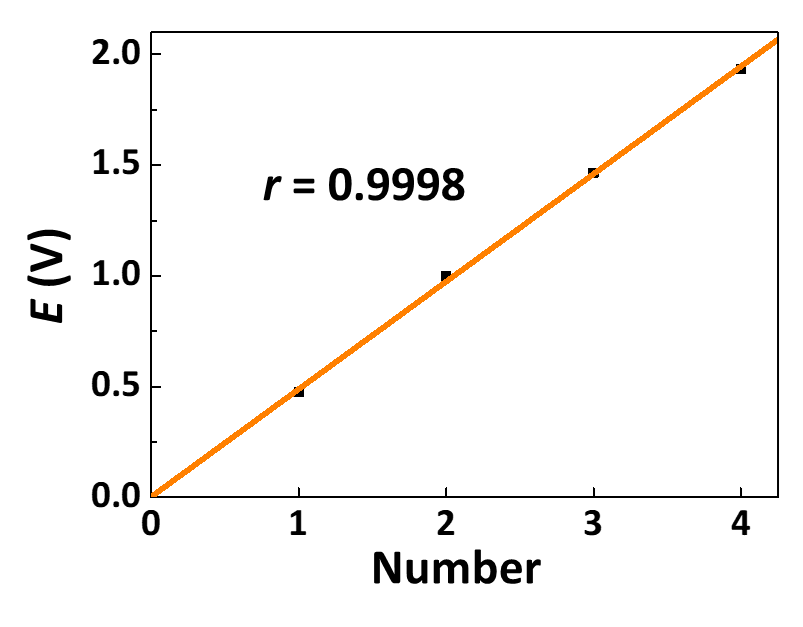


**Figure S37.** Linear fitting of the voltage output of WV_1.0_-MEG from Figure 5f versus number connected in series.


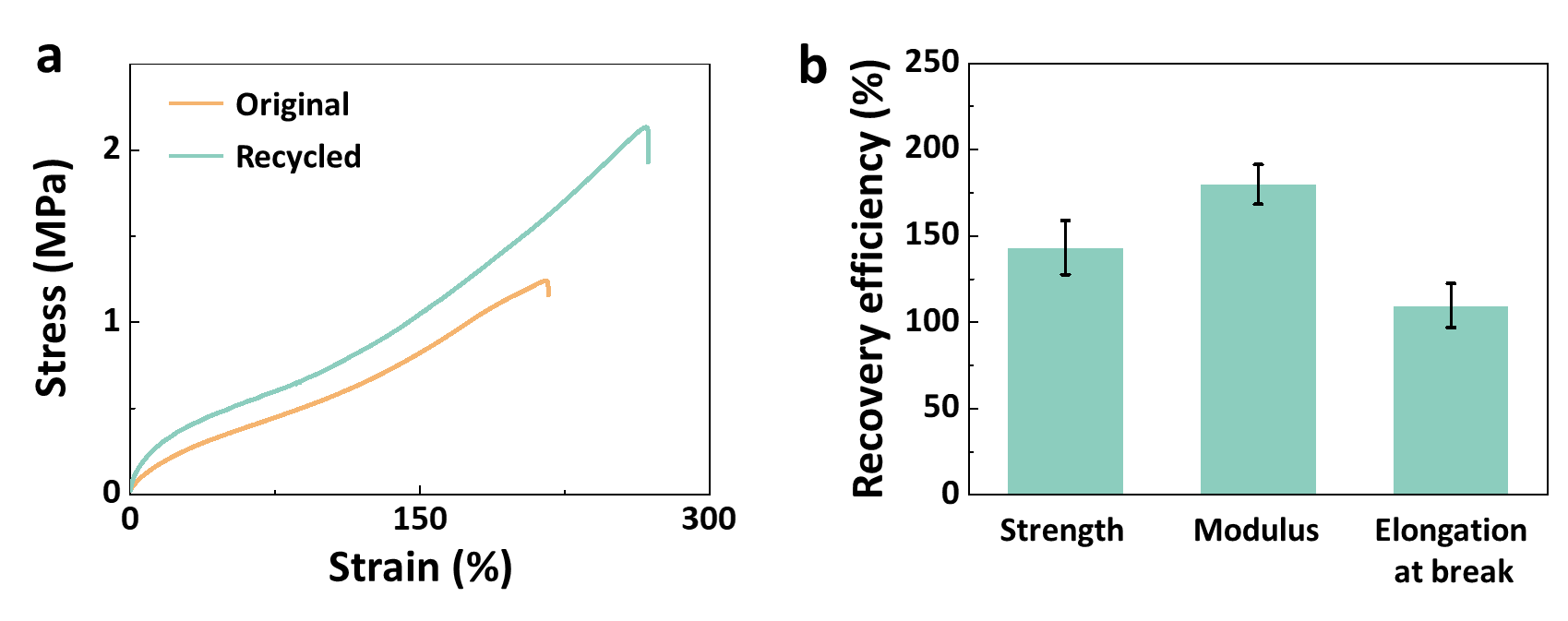


**Figure S38.** Comparison of mechanical properties between original and recycled waterborne vitrimers. (a) Stress-strain curves of recycled WV_1.0_ comparing with original one by tensile tests. (b) Recovery efficiency of tensile strength, modulus, and elongation at break determined by conducting tensile tests on the recycled vitrimers. Error bars represent the SD derived from quintuplicate measurements. The stretching rate of 5 mm min^-1^.


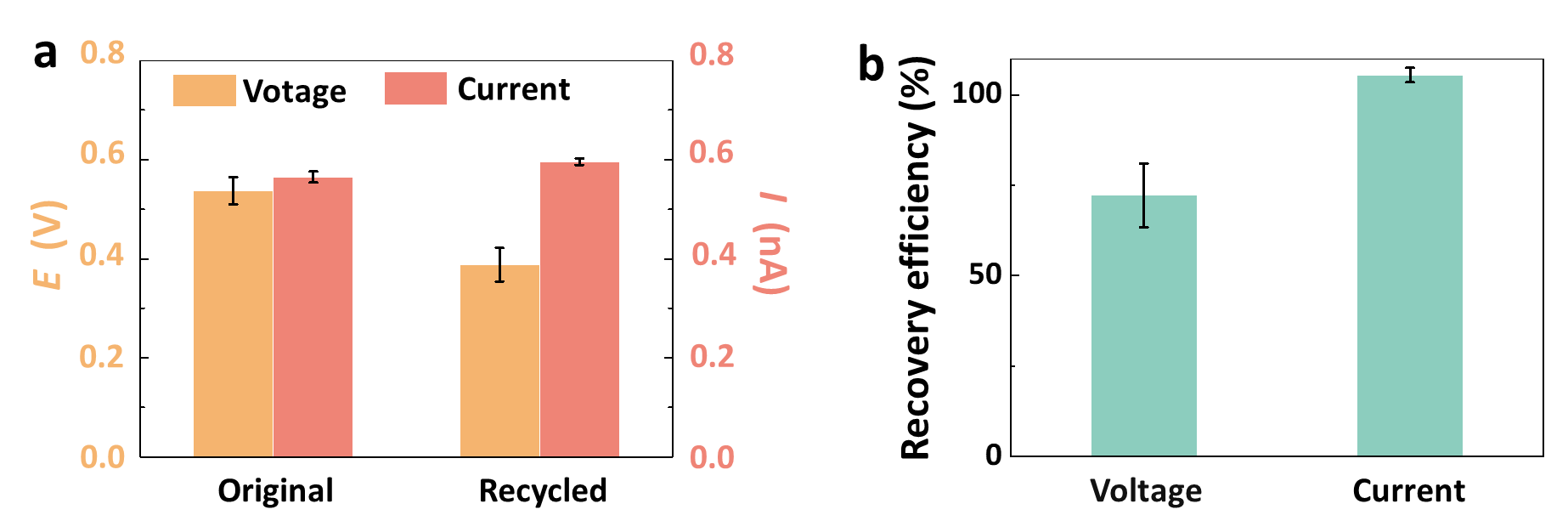


**Figure S39.** Comparison of moist-electric generation performance between original and recycled waterborne vitrimers. (a) Voltage output and current of moist-electric generators derived from original and recycled WV_1.0_. (b) Recovery efficiency of voltage output and current determined by moist-electric generators derived from WV_1.0_. Error bars in (a) and (b) of voltage output and current represent the SD derived from triplicate measurements.


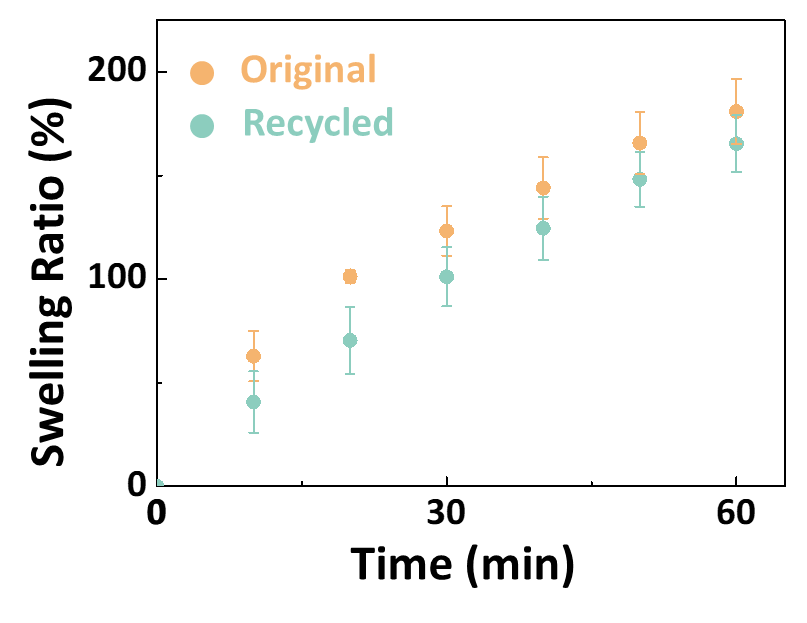


**Figure S40.** Swelling ratio of WV_1.0_ before and after recycling. Error bars represent the SD derived from triplicate measurements.

**S3. Supporting Tables**

**Table S1.** Constitution, thermal and mechanical properties of WV_1.2_, WV_1.0_ and WV_0.8_

| Waterborne vitrimer | Molar ratio  epoxy/  carboxyl | *T*_g_^a^  (^o^C) | *T*_d5_^b^  (^o^C) | *T*_α_^c^  (^o^C) | *ν*_e_^d^  (mol·m^-3^) | Tensile strength^e^  (MPa) | Young’s modulus^e^  (MPa) |
| --- | --- | --- | --- | --- | --- | --- | --- |
| WV_1.2_ | 1.2 | -9 | 250 | -6 | 23 | 0.60 ± 0.04 | 0.57 ± 0.11 |
| WV_1.0_ | 1.0 | 5 | 263 | 8 | 117 | 1.18 ± 0.08 | 1.41 ± 0.29 |
| WV_0.8_ | 0.8 | 9 | 258 | 27 | 84 | 1.68 ± 0.11 | 13.06 ± 1.50 |

^a^*T*_g_ values were decided from the point corresponding to the maximum negative slope (inflection point) in the decreasing segment of the DSC curves in Figure 2d; ^b^*T*_d5_ values were decided from Figure 2e; ^c^*T*_α_ values were decided from the point corresponding to the maximum negative slope (inflection point) in the decreasing segment of the storage modulus curves in Figure 2f; ^d^*ν*_e_ was decided from Figure 2f; ^e^Tensile strength and Young’s modulus were from stress-strain curves in Figure 2g.

**Table S2.** Gel content of WV_1.2_, WV_1.0_ and WV_0.8_ in different aprotic solvents

| Solvent | Gel content (%) | | |
| --- | --- | --- | --- |
|  | WV_1.2_ | WV_1.0_ | WV_0.8_ |
| DCM | 98.54 ± 0.77 | 98.97 ± 0.69 | 99.78 ± 0.23 |
| THF | 94.59 ± 2.35 | 98.01 ± 1.56 | 94.40 ± 3.20 |
| EtOAc | 97.82 ± 0.82 | 95.50 ± 1.41 | 97.59 ± 0.34 |
| Acetone | 88.56 ± 0.41 | 85.29 ± 3.73 | 92.79 ± 2.27 |
| Acetonitrile | 96.77 ± 2.21 | 99.07 ± 0.37 | 98.86 ± 0.66 |
| NVP | 96.74 ± 3.88 | 99.70 ± 0.42 | 94.75 ± 1.86 |
| DMSO | 87.71 ± 1.40 | 92.31 ± 2.23 | 90.00 ± 1.64 |

**Table S3.** The relaxation time of waterborne vitrimers and other TERs-based vitrimers

| Entry | Sample | Catalyst-free | Relaxation time | Regulation  strategy | Reference^a^ |
| --- | --- | --- | --- | --- | --- |
| 1 | DGEBA vitrimer  (zinc acetate) | × | 1200 s (190^o^C) | None | [1] |
| 2 | DGEBA vitrimer  (TBD at 5 mol %) | × | 4000 s (170^o^C) | Catalyst | [14] |
| 3 | Polyester vitrimer  (CL-PE-1) | × | 1153 s (160^o^C) | Cross-linking density | [16] |
| 4 | High-Lignin-derived vitrimer (PEG-epoxy/L-COOH *R* = 1:1) | × | 2400 s (160^o^C) | Multiple hydroxyl | [23] |
| 5 | Hyperbranched vitrimer  (HBE-2/SA-CAT) | × | 3540 s (180^o^C) | Multiple hydroxyl | [20] |
| 6 | Hyperbranched vitrimer  (HBE-1/SA) | √ | 10740 s (160^o^C) | Multiple hydroxyl | [20] |
| 7 | Poly(*β*-amino esters) (BE_1_/BG_2_) | √ | 31 s (160^o^C) | NGP effect  dual-dynamic covalent bonds | [19] |
| 8 | Ferulic acid-derived vitrimer  (CA/FEP/FEHBP) | √ | 22 s (160^o^C) | Multiple hydroxyl | [22] |
| 9 | Tertiary amine derived vitrimer  (EN.17-GA) | √ | 7620 s (160^o^C) | Multiple hydroxyl  NGP effect | [34] |
| 10 | Meldrum’s acid functionalized vitrimer (X_MAMA/BE188-V) | √ | 892 s (220^o^C) | NGP effect | [35] |
| 11 | Tannic acid-based vitrimer (S3) | √ | 5870 s (160^o^C) | Multiple hydroxyl | [24] |
| 12 | Itaconic acid-based vitrimer  (EMG-1.24) | √ | 6000 s (190^o^C) | Multiple hydroxyl | [36] |
| 13 | Acrylic vitrimer  (MG) | √ | 3000 s (160^o^C) | NGP effect | [37] |
| 14 | Alicyclic anhydride monoester based vitrimer (HTE-1.25) | √ | 4000 s (160^o^C) | NGP effect | [38] |
| 15 | Waterborne vitrimer | √ | 1828 s for WV_1.2_, 2365 s for WV_1.0_,  1132 s for WV_0.8_ at 160^o^C | Multiple hydroxyl  NGP effect | **This work** |

^a^Reference numbers are consistent with those in main text.

**Table S4.** Comparison of the chemical degradation of WV_1.0_ with other reported vitrimers

| Entry | Dynamic  covalent bond | Catalyst | Degradation  medium | Degradation condition | Reference^a^ |
| --- | --- | --- | --- | --- | --- |
| 1 | Ester bonds | √ | Pure water | 180^o^C for 5 h | [34] |
| 2 | Ester bonds | √ | Pure water | 200^o^C for 10 h | [39] |
| 3 | Ester bonds | √ | Methanol | 70^o^C for 48 h | [40] |
| 4 | Ester bonds | √ | 1 M NaOH | 50^o^C for 40 min | [36] |
| 5 | Ester bonds | √ | 30 wt% NaOH | 80^o^C for 30 min | [22] |
| 6 | Disulfide bonds | √ | 2-Mercaptoethanol in DMF | Room temperature for 24 h | [41] |
| 7 | Imine bonds | √ | Diethylenetriamine | Room temperature for 24 h | [42] |
| 8 | Urethanes | √ | Tetraethylene glycol | 180^o^C for 4 h | [43] |
| 9 | Oxime-urethanes | √ | DMAc | 110^o^C for 8 h | [44] |
| 10 | Knoevenagel adducts | √ | 0.5 M NaOH in acetonitrile | 60^o^C for 24 h | [45] |
| 11 | Ester bonds | √ | Pure water | 95^o^C for 6 h | **This work** |

^a^Reference numbers are consistent with those in main text.

**Table S5.** Proposed chemical structures of potential degradation products and their corresponding fragments in the mass spectrometry (MS) spectrum

| Molecular weight | Chemical formula | Chemical structure | Corresponding figure |
| --- | --- | --- | --- |
| **150** | C_4_H_6_O_6_ |  | Figure 3g_1_ |
| **370** | C_14_H_26_O_11_ |  | Figure 3g_2_ |
| **370** | C_14_H_26_O_11_ |  | Figure 3g_3_ |
| **590** | C_24_H_46_O_16_ |  | Figure 3g_4_ |
| **722** | C_28_H_50_O_21_ |  | Figure 3g_4_ |
| **854** | C_32_H_54_O_26_ |  | Figure 3g_4_ |
| **942** | C_38_H_70_O_26_ |  | Figure S29 |
| **1074** | C_42_H_74_O_31_ |  | Figure S29 |
| **1206** | C_46_H_78_O_36_ |  | Figure S29 |

**Table S6.** Gel content of WV_1.2_, WV_1.0_ and WV_0.8_ in water and other protic solvents

| Solvent | Gel content (%) | | |
| --- | --- | --- | --- |
|  | WV_1.2_ | WV_1.0_ | WV_0.8_ |
| Water | 70.42 ± 6.19 | 68.21 ± 3.27 | 78.73 ± 1.87 |
| MeOH | 70.70 ± 1.87 | 68.73 ± 1.13 | 77.79 ± 2.03 |
| HAc | 76.36 ± 1.08 | 76.86 ± 2.48 | 84.44 ± 2.20 |
| EtOH | 80.79 ± 0.99 | 81.25 ± 1.35 | 89.10 ± 1.54 |

**Table S7.** Comparison of the of WV_1.0_ with other traditional polymer moisture-driven actuators

| Entry | Materials | Thickness | Response rate | Additional  characteristics | Reference^a^ |
| --- | --- | --- | --- | --- | --- |
| 1 | Polyurethane | 17 μm | Curvature 2.2 cm^-1^/16 s | Heat and light triggered deformation | [S4] |
| 2 | Polyurethane | 40 μm | 43^o^/1.4 s | Water resistance | [S5] |
| 3 | CaCO_3_ oligomers/PVDF | 15 μm | Curvature 8.67 cm^-1^/1 s | Self-motivation on ice surface | [S6] |
| 4 | Tri-layer by  PAM hydrogel/  PET/PEA | 8 μm | 405^o^/8 s | Moisture-controlled electric switches | [S7] |
| 5 | Cross-linked CNF | 7 μm | Curvature 0.72 cm^-1^/3.4 s | Water resistance  Good mechanical properties | [S8] |
| 6 | Bilayer by Microfibrillated cellulose/CNF | 55 μm | 60^o^/12 s | Biodegradability | [S9] |
| 7 | Epoxy vitrimer | 40 μm | 360^o^/115 s | Recyclability and degradablity  Moist-electric generation | **This work** |

^a^Reference numbers are consistent with those in Supporting Information.

**4. Supporting References**

[S1] Treloar, L. R. G. *The Physics of Rubber Elasticity*; Oxford University Press: Oxford, **1975**.

[S2] M. Capelot, M. M. Unterlass, F. Tournilhac, L. Leibler. *ACS Macro Lett.* **2012**, *1*, 789-792.

[S3] J. Luo, Z. Demchuk, X. Zhao, T. Saito, M. Tian, A. P. Sokolov, P.- F. Cao. *Matter* **2022**, *5*, 1391-1422.

[S4] X. Yue, C. Dong, Y. Wang, Z. Cui, Z. Ren, Z. -H. Guan. *Chem. Eng. J.* **2023**, *457*, 141290.

[S5] W. Liu, X. Yang, G. Yan, L. Wang, J. Lai, Z. Li, C. Zhao, D. Xiang, H. Li, Y. Wu. *Small* **2024**, *20*, 2400482.

[S6] Y. He, K. Kong, Z. Guo, W. Fang, Z. Ma, H. Pan, R. Tang, Z. Liu. *Adv. Funct. Mater.* **2021**, *31*, 2101291.

[S7] J. Li, G. Zhang, Z. Cui, L. Bao, Z. Xia, Z. Liu, X. Zhou. *Small* **2023**, *19*, 2303228.

[S8] B. Li, X. Zhu, C. Xu, J. Yu, Y. Fan. *Carbohyd. Polym.* **2024**, *335*, 122108.

[S9] J. Zhu, P. Zhu, Y. Ye, Y. Zhang, X. Sun, P. Yang, P. Servati, F. Jiang, *Nano Lett.* **2024**, *24*, 14073-14081.
